# Extracellular Vesicles from iPSC-Derived Glial Progenitor Cells Prevent Glutamate-Induced Excitotoxicity by Stabilising Calcium Oscillations and Mitochondrial Depolarisation

**DOI:** 10.3390/cells14231915

**Published:** 2025-12-02

**Authors:** Margarita Shedenkova, Anastasiia Gurianova, Irina Krasilnikova, Anastasia Sudina, Evgeny Karpulevich, Yaroslav Maksimov, Marina Samburova, Egor Guguchkin, Zlata Nefedova, Valentina Babenko, Daniil Frolov, Kirill Savostyanov, Timur Fatkhudinov, Dmitry Goldshtein, Zanda Bakaeva, Diana Salikhova

**Affiliations:** 1 Research Institute of Molecular and Cellular Medicine, Medical Institute of RUDN University, Moscow 117198, Russiamarina_samburova33@mail.ru (M.S.);; 2 Research Centre for Medical Genetics, Moscow 115522, Russia; 3 Ivannikov Institute for System Programming of the Russian Academy of Sciences, Moscow 109004, Russia; 4 National Medical Research Center for Children’s Health, Moscow 119991, Russia; 5 FSAEI HE I.M. Sechenov First MSMU of MOH of Russia, Sechenovskiy University, Moscow 119991, Russia; zlanefedova@gmail.com; 6 Belozersky Institute of Physico-Chemical Biology, Lomonosov Moscow State University, Moscow 119991, Russia; 7 Kulakov National Medical Research Center of Obstetrics, Gynecology, and Perinatology, Moscow 117997, Russia; 8 Institute of Information Technology, Russian Technological University (RTU MIREA), Moscow 119454, Russia; 9 Federal State Budgetary Scientific Institution “Research Institute of Human Morphology”, Moscow 117418, Russia

**Keywords:** extracellular vesicles, neuroprotection, glutamate, proteome, transcriptome, glial progenitor cells

## Abstract

Neurodegenerative diseases pose a significant challenge to modern medicine. Despite significant advances in neurology, current therapeutic approaches often prove insufficient to treat such disorders. This study investigates the neuroprotective effect of extracellular vesicles derived from glial derivates of human-induced pluripotent stem cells. The extracellular vesicle’s cargo was characterised by proteomic analysis. The neuroprotective effect was assessed using a model of glutamate excitotoxicity performed on a primary culture of cortical neuroglial cells. The viability of cells was estimated using the MTT test and morphometric analyses. A comprehensive methodology was applied to investigate intracellular mechanisms, integrating assessments of intracellular calcium concentrations, mitochondrial membrane potential, and targeted inhibition of the PI3K-Akt pathway. Transcriptomic analysis of neuroglial cultures was used to validate the role of obtained mechanisms of extracellular vesicle’s neuroprotective effect. The obtaining results demonstrated the improvement of neuronal survival by reducing intracellular calcium levels and stabilising mitochondrial membrane potential under glutamate-induced excitotoxicity via PI3K-Akt signalling pathway activation. Moreover, the vesicles contained proteins that contribute to preventing apoptotic processes, activating regeneration of the nervous system, and modulating calcium ion transport and are associated with redox processes. Further transcriptomic analyses of neuroglial cultures treated with EVs showed an up-regulation of genes associated with regeneration, inhibition of calcium ion transport, regulation of membrane depolarisation, and negative regulation of apoptotic pathways.

## 1. Introduction

Neurological disorders are a large group of diseases of the nervous system characterised by a variety of clinical manifestations and developmental mechanisms. According to the World Health Organization, neurological disorders are one of the most common causes of disability in the population. The increase in diseases such as Alzheimer’s disease, cerebrovascular diseases including stroke, demyelinating diseases, traumatic brain injury, and others is a major medical and social problem.

One of the most important pathogenetic mechanisms of damage to the nervous system in various neurological diseases is excitotoxicity. Research into the mechanisms of glutamate excitotoxicity is of fundamental importance for understanding the pathogenesis of such diseases and opens up new perspectives for the development of effective methods of prevention and treatment. Glutamate, the most important excitatory neurotransmitter of the brain, plays a special role in this process, whereby an excess of glutamate can lead to irreversible consequences for the neurons. In transsynaptic transmission, glutamate then activates the glutamate receptors on the postsynaptic membrane after it releases. This leads to the activation of the receptors and entry of calcium ions into the neurons, then to depolarisation of the membrane, transmission of an electrical signal from the dendrite to the soma of the neuron, and the initiation of metabolic cascades that affect intracellular homeostasis [1]. However, a high concentration of glutamate molecules in the synaptic cleft has a hyperstimulatory effect on the receptors. This promotes a more active flow of calcium ions into the neurons. High calcium concentrations disrupt mitochondrial function, stimulate the production of reactive oxygen species, inhibit protein folding, and activate endoplasmic reticulum stress, leading to the initiation of apoptotic cascades and cell death [2].

Currently, there are many drugs that affect glutamatergic transmission by inhibiting glutamate receptors (memantine, tiglutik [3,4]), by reducing the synthesis of this neurotransmitter (tiglutik), by slowing the flow of calcium and sodium ions into cells (riluzone [5]), or by promoting the synthesis of glutamate inhibitors (cholinesterase inhibitors [6]). The Food and Drug Administration (FDA) approves these drugs to treat Alzheimer’s disease, amyotrophic lateral sclerosis, and stroke, but they only have a short-term effect. Cell therapy is a universal multitarget approach that has shown high efficacy in brain pathologies in many in vivo models [7]. The beneficial effects of multipotential mesenchymal stem cell (mMSC) treatment include anti-apoptotic activity, activation of neuro- and angiogenesis, and immunomodulation, which together may have a neuroprotective effect. However, the use of cell therapy has several disadvantages, including a low percentage of cell survival [8,9], the risk of oncogenicity [10,11,12], and a high immunogenicity of the therapy, which may neutralise the positive effect of the treatment [13]. There are no approved cell drugs to treat neurological diseases, and the development of a multitarget therapy that can act simultaneously on several targets remains an urgent task for modern medicine.

Numerous experimental data suggest that paracrine activity mediates the positive therapeutic effect of multipotential stem cell (MSC) transplantation [14], and not by the replacement of lost cells [15], which is confirmed by the presence of neuroprotective factors in their secretome [16,17]. It includes proteins, signalling molecules, nucleic acids, and extracellular vesicles (EVs) that may have therapeutic effects, including neuroprotection [18]. To date, extracellular vesicles are one of the most promising tools in regenerative medicine. They represent a new class of multitarget therapeutics and show a polyvalent effect by transporting a specific cargo complex of proteins, peptides, nucleic acids, and metabolic components. In addition, extracellular vesicles have a number of advantages. They retain their content in the blood, lymph, or tissue fluid because of the bilipid layer, bind specifically to target cells via specific receptors on the membrane surface, and can cross the blood–brain barrier [19]. The properties described above determine the high therapeutic potential of extracellular vesicles as a promising group of therapeutics for the pathogenetic correction of neurodegenerative diseases and the restoration of functional activity of the nervous system. Numerous studies are limited to analysing the therapeutic effect of EVs derived from MSCs of various origins in models of neurological diseases. The most suitable source for obtaining EVs are cells of the nervous tissue, for example, neuroglia, which are a series of auxiliary cells for neurons and fulfil metabolic, structural, homeostatic, and neuroprotective functions, such as the removal of excess neurotransmitters, the formation of synapses, the maintenance of neuronal homeostasis, and the stabilisation and regulation of the blood–brain barrier. The glial cells perform these functions through direct contact with the target cells and through paracrine influence by EVs, cytokines, and other signalling molecules [20]. In a traumatic brain injury model, the therapeutic effect of extracellular vesicles derived from human glial progenitor cells (EV-GPCs) has been demonstrated, but the neuroprotection mechanisms are not well understood [21]. Therefore, the aim of this study was to investigate the mechanisms of neuroprotective effects of extracellular vesicles derived from human GPCs in a model of glutamate excitotoxicity.

## 2. Materials and Methods

### 2.1. Isolation of Glial Progenitor Cell-Derived Extracellular Vesicles (EV-GPCs)

Cultures of glial progenitor cells (GPCs), used for the EV preparation, were previously derived from a healthy donor by differentiating induced pluripotent stem cells (iPSCs) towards a glial lineage [22]. Informed consent was obtained from the patient before taking a skin biopsy. The protocol was approved by the institutional ethics committee of Research Centre for Medical Genetics (Protocol No. 2019-2/3 dated 13 October 2020). GPCs were cultured to monolayer confluence in DMEM/F12 medium (PanEco, Moscow, Russia) supplemented with 1% N2 supplement (PanEco, Moscow, Russia), 1% fetal bovine serum (FBS, Gibco, Carlsbad, CA, USA), 1 mM glutamine (PanEco, Russia), 50 U/mL penicillin/streptomycin (PanEco, Russia), 20 ng/mL EGF (PeproTech, Cranbury, NJ, USA), and 20 ng/mL CNTF (PeproTech, Cranbury, NJ, USA). To obtain the conditioned medium (CM) containing EVs, cells were washed with Hank’s solution and incubated for 24 h in serum-free culture medium. The conditioned medium was centrifuged at 10,000× *g* for 30 min to separate apoptotic bodies and then at 108,000× *g* for 1.5 h twice: first to deposition extracellular vesicles, and then to wash them free of culture medium using an Avanti JXN-30 ultracentrifuge equipped with a JA-30.50 Ti rotor (Beckman Coulter Inc., Brea, CA, USA). The resulting vesicle pellet was validated according to previously described methodologies. The prepared EV aliquots were stored at −80 °C.

### 2.2. Characteristics of the EV-GPCs

#### 2.2.1. Nanoparticle Tracking Assay

Extracellular vesicle concentration was analysed using a Nanosight LM10 HS instrument (NanoSight Ltd., Amesbury, UK) to determine particle size and number. A 2 μL aliquot of the extracellular vesicle suspension was diluted with a phosphate buffer solution (PanEco, Moscow, Russia) to a total sample volume of 2 mL (1:1000). Samples were injected into the instrument using a sterile 5 mL syringe. The recording and subsequent analysis settings were set manually according to the protocol (camera level 13, shutter speed 1232, shutter gain 219, particle detection threshold 8). Recording was performed at room temperature. Extracellular vesicles were visualised using laser light scattering, and their Brownian motion was recorded on video. Video recordings were processed using Nanoparticle Tracking Analysis software version 3.3 (NanoSight Ltd., Amesbury, UK). At least five separate videos were recorded and processed for each sample, each 60 s long. Data from multiple videos were combined to create a particle size histogram.

#### 2.2.2. Transmission Electron Microscopy

EV-GPCs were dispensed in 10 µL aliquots on nitrocellulose carbon-coated PELCO^®^ Cu grids (Ted Pella Inc., Redding, CA, USA) and incubated for 1 min. Then 10 µL of 2% uranyl acetate was applied, the grids were incubated for 15 s, and excess liquid was removed with nitrocellulose paper. The samples were examined at 80 kV using a JEM-1011 transmission electron microscope (JEOL, Akishima, Japan) equipped with an Orius™ SC1000 W camera (Gatan, Inc., Pleasanton, CA, USA).

#### 2.2.3. Western Blot Analysis

The EV-GPC aliquot was mixed with 4× Laemmli Sample Buffer (Bio-Rad, Hercules, CA, USA). Prior to gel loading, all samples were heated at 99 °C for 10 min. The gels were prepared using TGX Stain-Free FastCast Acrylamide Starter Kit, 10% (Bio-Rad, Hercules, CA, USA) in accordance with the manual. The protein transfer to nitrocellulose membranes was performed electrically using a Trans-Blot Turbo Transfer System (Bio-Rad, Hercules, CA, USA). The membranes were blocked in PBS with 5% BSA overnight. Next day, the membranes were incubated with primary antibodies diluted in 0.1% Tween-20 and 1% BSA: CD63 (1:500, AF5117, Affinity Biosciences, Cincinnati, OH, USA) and CD9 (1:500, DF6565, Affinity Biosciences, Cincinnati, OH, USA) for EV-GPC characterisation. The membranes were washed from primary antibodies and exposed to secondary antibodies, anti-rabbit IgG conjugated with Alexa Fluor 488 (1:1000, A-11008), for 60 min in the dark. The immunoblots were documented in a BioRad Chemidoc Imaging System (Bio-Rad, Hercules, CA, USA).

### 2.3. Preparation of Neuroglial Culture

Newborn Wistar rats (P1-P2) were used to obtain primary neuroglial cultures, as described previously [23]. Animals were euthanised by inhalation of a lethal dose of isoflurane (Aerrane, Baxter HealthCare Corporation, Deerfield, IL, USA), decapitated, and the brains were extracted. The cerebral cortex was isolated and cleared of the vascular lining. A suspension of cortical neuroglial cells (10^6^ cells/mL) was obtained by treating the brain tissue with papain (10 U/mL) and dissociating by pipetting with subsequent deposition (200× *g*) in Neurobasal medium (Gibco, Carlsbad, CA, USA). The cells were seeded into 48-well flat-bottom plates (2.5 × 10^5^ cells/well) (Corning Inc., Corning, NY, USA) and into Petri dishes (Ø35 mm) with a glass-bottom insert (Ø14 mm) (MatTeck, Ashland, MA, USA). The plates and Petri dishes were pre-coated with polyethylenimine (0.05 mg/mL, 60 min). After one hour, 1.5 mL of Neurobasal medium (NBM) containing 2% B-27 supplement, 1% GlutaMAX, and 1% antibiotic/antimycotic solution (all reagents from Gibco, USA) was added. Cytosine arabinoside (1 µM) was added to the medium on day 3 in vitro (DIV) to prevent excessive glial cell proliferation. Cells were incubated for 10–14 days in vitro (DIV) at 37 °C in a humidified atmosphere of 5% CO_2_/95% air. A partial culture medium change was performed every three days.

### 2.4. Immunohistochemistry of Neuroglial Culture

To obtain the immunophenotypic characterisation of the resulting primary culture, an immunocytochemical test for the neuronal marker beta-III-tubulin (TUBB3) and the astrocytic marker of glial fibrillary acidic protein (GFAP) was carried out. For immunocytochemical analysis, cells were fixed with 4% formaldehyde solution (Merck KGaA, Darmstadt, Germany) for 10 min at room temperature and permeabilised with 0.25% Triton X-100 solution (Sigma-Aldrich, St. Louis, MO, USA) for 30 min. Then they were incubated overnight at +4 °C with primary anti-TUBB3 (1:500, ab78078 Abcam, Cambridge, UK) and anti-GFAP (1:600, ab7260 Abcam). Next day cells were incubated in the dark for 60 min with anti-mouse secondary antibodies Alexa Fluor 555 (1:600) or anti-rabbit secondary antibodies Alexa Fluor 488 (1:600) (Invitrogen, Waltham, MA, USA). Nuclei were stained with a solution of DAPI (4,6-diamidino-2-phenylindole dihydrochloride (Sigma-Aldrich, USA) in 1 mg/mL in phosphate-buffered solution. Images were obtained using an inverted fluorescence microscope Leica DMi8 (Leica Biosystems, Nussloch, Germany) with a 20×/0.35 dry objective, equipped with a Leica DFC7000 T camera and Leica Microsystems Imaging Software (version Leica LAS X 5.3.1).

### 2.5. Induction of Glutamate Excitotoxicity In Vitro

The primary neuroglial cultures at 9-10 DIV were used to establish the model of glutamate excitotoxicity. The culture was washed twice with a buffer solution of the following composition: 140 mM NaCl, 5 mM KCl, 2 mM CaCl_2_, 5 mM glucose, and 20 mM HEPES (pH ~7.4), followed by the addition of a solution containing 100 µM glutamate, 140 mM NaCl, 5 mM KCl, 10 mM glycine, 2 mM CaCl_2_, 5 mM glucose, and 20 mM HEPES (pH ~7.4) and incubation was continued for 1 h. Thereafter, cells were washed twice with the buffer solution and returned to the conditioned culture medium, which had been collected beforehand. The EV-GPC preparation was added 24 h prior to model induction at a concentration of 10 µg/mL. To assess the involvement of the PI3K-Akt signalling pathway, the selective PI3Kγ subunit inhibitor AS605240 (1 µg/mL) was used, which was added together with the EV-GPCs. Memantine (100 µM)—a low-affinity, voltage-dependent, non-competitive antagonist of N-methyl-D-aspartate (NMDA) glutamate receptors—was used as a positive control and was added together with the glutamate-containing solution. Viability assessment was performed on the day following the modelling of glutamate excitotoxicity.

### 2.6. Assessment of Cell Viability Using the MTT Test and Morphometric Evaluation of Neuronal Death

Cell survival was assessed using the MTT test—a colorimetric assay for which MTT reagent (3-[4,5dimethylthiazol-2-thiazolyl]-2,5-diphenyl-tetrazolium bromide dissolved in phosphate-buffered saline (pH = 7.4) (Merck, Germany) to a concentration of 5 mg/mL was used. The resulting stock solution was added to the cell culture medium (final concentration 0.5 mg/mL) and incubated for 1.5 h at 37 °C. The culture medium was then aspirated and DMSO (Thermo Fisher Scientific, Waltham, MA, USA) was added. The staining intensity was measured using a ClarioStars multimodal plate reader (BMG Labtech, Ortenberg, Germany) at a wavelength of 520 nm with a reference wavelength of 690 nm. The obtained data were normalised, with the result in control cultures taken as 100%.

Morphometric assessment of neuronal death included the evaluation of nuclear fragmentation (apoptosis) by live cell staining for 30 min with the fluorescent dye Hoechst 33342 Ready Flow™ Reagent (Invitrogen, Waltham, MA, USA) at a concentration of 5 µg/mL and the vital dye 0.5 µM Calcein AM (percentage of live cells). The number of necrotic cells was assessed using 1 µg/mL propidium iodide (PI) with a 10 min incubation, which penetrates cells with a compromised plasma membrane. Live cell imaging of cultures was then performed using a Leica DMi8 fluorescence microscope (Germany) with a 20×/0.35 dry objective, equipped with a Leica DFC7000 T camera and Leica Microsystems Imaging Software. Image processing and analysis were performed using ImageJ software ver. 1.54g.

### 2.7. Proteomic Analysis of Extracellular Vesicles

Extracellular vesicle samples were lysed in a buffer containing 4% SDS, 50 mM TRIS-HCl (pH = 8), and protease inhibitors (Sigma-Aldrich) for 30 min at 60 °C on a thermoshaker. The samples were then sonicated (3 cycles of 10 pulses at 30% amplitude). Subsequently, protein was precipitated using chilled acetone for 14 h (sample-to-acetone ratio 1:10). After precipitation, samples were centrifuged at 4 °C, 16,000× *g* for 10 min; the pellets were washed once with chilled acetone. After centrifugation (4 °C, 16,000× *g*, 10 min), the pellets were dried in a vacuum concentrator and dissolved in a buffer (8 M urea, 2 M thiourea, 10 mM Tris-HCl (pH = 7.5)) for 20 min at 24 °C. Protein concentration in the samples was measured by the Bradford method (Bio-Rad) according to the manufacturer’s protocol. An equal amount of protein from each sample was then aliquoted for proteomic analysis. For the reduction of disulfide bonds, samples were incubated in a 5 mM DTT solution at 24 °C for 30 min, followed by alkylation by incubating samples in a 10 mM iodoacetamide solution at room temperature for 20 min in the dark. Alkylated samples were diluted with a 50 mM ammonium bicarbonate solution at a 1:4 ratio; trypsin solution was then added to the samples (0.01 µg trypsin (Promega, Madison, WI, USA; LC-MS/MS grade) per 1 µg of protein), and they were incubated at 37 °C for 12 h. After hydrolysis, the reaction was stopped by adding formic acid to a final concentration of 5% in the solution. The resulting tryptic peptides were desalted using SDB-RPS microcolumns, dried in a vacuum concentrator, and stored at −80 °C until LC-MS/MS analysis.

Peptide fractions after trypsin digestion were injected in an aqueous solution containing 3% acetonitrile and 0.1% TFA onto a column (75 µm inner diameter, 25 cm length) packed with Aeris Peptide XB-C18 2.6 µm material (Phenomenex, Torrance, CA, USA). Peptide separation was performed on an Ultimate 3000 Nano LC System (Thermo Fisher Scientific, Waltham, MA, USA) coupled to a Q Exactive HF mass spectrometer (Thermo Fisher Scientific) via a nanoelectrospray ion source (Thermo Fisher Scientific). Peptides were loaded onto the column, thermostatted at 40 °C in buffer A (0.2% formic acid (FA) in water), and eluted with a linear (120 min) gradient of 4 > 55% buffer B (0.1% FA, 19.9% water, 80% acetonitrile) in buffer A at a flow rate of 350 nL/min. Before each new injection, the column was washed with 95% buffer B in A for 5 min and equilibrated with buffer A for 5 min. Mass spectrometric data were acquired in a data-dependent acquisition mode automatically switching between 1 MS1 scan and up to 15 MS/MS scans (topN method). The target value for MS1 scans was set to 3 × 10^6^ in the range of 300–1200 *m/z* with a maximum ion injection time of 60 ms and a resolution of 60,000. Isolation of precursor ions was performed with a 1.4 *m*/*z* window and a fixed first mass of 100.0 *m*/*z*. Precursor ions were fragmented by higher energy collisional dissociation (HCD) in the C-trap with a normalised collision energy of 28 eV. MS/MS scans were acquired with a resolution of 15,000 at *m*/*z* 400 and a target value of 1 × 10^5^ for ions in the range of 200–2000 *m/z* with a maximum ion injection time of 30 ms.

Raw mass spectrometric data from the instrument were converted into MGF (Mascot generic format) peak lists using MSConvert (ProteoWizard Software Foundation, version 3). The following parameters were used for this procedure: --mgf–filter peakPicking true. For thorough protein identification, the generated peak list was analysed using MASCOT (version 2.5.1) and X! Tandem (ALANINE (2017.02.01)) search engines against the UniProtKB database, taxon Homo sapiens. Statistical validation of identifications was performed based on a search against a decoy reversed database of protein sequences. Allowed mass deviations for the precursor and fragment ions were 20 ppm and 0.04 Da, respectively. Database search parameters were as follows: allowance for one missed cleavage site for trypsin, fixed modification—carbamidomethylation (C), and dynamic modification—oxidation (M). For X! Tandem, parameters allowing for the rapid checking of N-terminal protein residue acetylation, loss of ammonia from N-terminal glutamine residues of peptides, or loss of water from N-terminal glutamic acid residues of peptides were also selected. The resulting files were loaded into Scaffold 4 software (version 4.0.7) for validation and meta-analysis. Peptides and proteins falling within the identification array with a local FDR of 5% were considered reliably identified.

Quantitative analysis of proteins was performed based on the spectral count method (“total spectral count”) of peptide fragments identified for the corresponding proteins. The resulting protein list was analysed for cellular compartment and biological process affiliation using the String 11.0 database.

### 2.8. Measurement of [Ca^2+^]_i_ and Mitochondrial Potential (ΔΨm) in Cortical Neuroglial Cells

Fluorescence measurements were performed on an experimental setup comprising an Olympus IX-71 inverted fluorescence microscope with 20× and 40× fluorite objectives, a Sutter Lambda 10-2 illumination system with a 175 W xenon lamp (Sutter Instruments, Novato, CA, USA), and a CoolSNAP HQ2 CCD camera (Photometrics, Tucson, AZ, USA), controlled by MetaFluor software (version 1.0.93) (Universal Imaging Corp., Bedford Hills, NY, USA).

Measurements of intracellular free Ca^2+^ concentration ([Ca^2+^]ᵢ) and mitochondrial membrane potential (ΔΨm) were performed on cortical neuroglial cells (9-11 DIV) cultured on Ø35 mm culture dishes with a glass bottom (MatTeck, Ashland, MA, USA) at a density of 2.5 × 10^5^ cells/dish, as described above. EV-GPCs were added to neuronal cultures 24 h prior to fluorescence microscopy measurements at a final concentration of 10 µg/mL. [Ca^2+^]ᵢ measurements were performed using the fluorescent low-affinity Ca^2+^ indicator Fura2 in its acetoxymethyl ester (AM) form (Thermo Fisher Scientific, USA) at a concentration of 2 µM, by incubation in the culture medium for 60 min at 37 °C and 5% CO_2_. To facilitate Fura2 penetration into cells, it was added as a suspension with the non-ionic detergent Pluronic F-127 (0.02%. Sigma-Aldrich, Louis, MO, USA). Fura2 fluorescence was alternately excited at 340 ± 5 and 380 ± 5 nm and recorded at 525 ± 25 nm (500 nm dichroic mirror). For simultaneous monitoring of changes in [Ca^2+^]ᵢ and ΔΨm, cells were loaded with the potential-sensitive dye Rhodamine123 (Rh123, 2.5 µg/mL), (Thermo Fisher Scientific, USA) for 15 min at 37 °C, the fluorescence of which was excited and recorded at 485 ± 5 and 525 ± 25 nm, respectively. Measurements were performed at 24–26 °C sequentially in buffers of the following composition: normal buffer (130 mM NaCl, 5 mM KCl, 2 mM CaCl_2_, 1 mM MgCl_2_, 20 mM HEPES, and 5 mM glucose (pH 7.4))—to record the fluorescence of both dyes under standard conditions; glutamate buffer (130 mM NaCl, 5 mM KCl, 2 mM CaCl_2_, 20 mM HEPES, 5 mM glucose, 100 µM glutamate, and 10 µM glycine (pH 7.4))—for 15 min; nominally Ca^2+^-free buffer (130 mM NaCl, 5 mM KCl, 2 mM MgCl_2_, 20 mM HEPES, 5 mM glucose, 10 µM glycine, and 100 µM EGTA (pH 7.4))—for 30 min to record the recovery of [Ca^2+^]ᵢ and ΔΨm by the cells; nominally Ca^2+^-free buffer with the addition of 1 µM protonophore carbonyl cyanide 4-(trifluoromethoxy)phenylhydrazone (FCCP)—for 5 min to assess the maximum Rh123 signal during mitochondrial depolarisation and the amount of Ca^2+^ accumulated by mitochondria. At the end of the experiment, 1 µM ionomycin ionophore (in the presence of 5 mM Ca^2+^ and without added Mg^2+^) was used to determine the maximum cytoplasmic calcium capacity. Image processing was performed using MetaFluor Analysis software (Universal Imaging Corp., Bedford Hills, NY, USA).

### 2.9. Transcriptomic Analysis (mRNA Sequencing)

To investigate the intracellular pathways activated in the presence of the EV-GPC preparation (two experimental groups: IC—intact culture, EV—culture with EV-GPC addition), total RNA analysis was performed via transcriptomic analysis. For this, total RNA was collected from cortical neuron cultures 4 h after EV-GPC addition using the RNeasy Plus Mini Kit (Qiagen, Hilden, Germany) according to the manufacturer’s instructions. Samples were stored and transported at −80 °C.

The next stage of the experiment was the transcriptomic analysis of neuroglial cultures after glutamate exposure. For this, cultures were treated with EV-GPC preparations 24 h prior to model induction. The following day, glutamate and memantine were added to the cultures as indicated in the methodology above. Thus, the experiment included 4 groups: IC—intact culture without glutamate addition, GL—cultures incubated with glutamate, GL_EV—cultures pre-incubated with EV-GPCs and then incubated with glutamate 24 h later, GL_MEM—cultures incubated with glutamate and memantine. Upon completion of the experiment, cultures were incubated for 4 h in culture medium, after which total RNA was collected as described above.

The obtained total RNA samples were treated with the Turbo DNA-Free Kit (Thermo Fisher Scientific) in a 50 µL volume and then purified using Agencourt RNAClean XP (Beckman Coulter Inc., Brea, CA, USA) according to the manufacturer’s instructions. Total RNA quantity was measured using the Quant-iT Ribogreen RNA assay kit (Thermo Fisher Scientific), and the quality of the isolated RNA was checked on an Agilent Bioanalyzer using Agilent RNA 6000 Pico Chips (Agilent Technologies, Santa Clara, CA, USA).

For transcriptomic library preparation, 250 ng of total RNA was used as the starting material. RNA libraries were prepared using the NEBNext Poly(A) mRNA Magnetic Isolation Module and the KAPA RNA HyperPrep Kit (Roche, Basel, Switzerland) according to the manufacturer’s protocol. RNA was then purified using the RNA Clean XP kit (Beckman Coulter, Brea, CA, USA) and the libraries were given a final cleanup using Agencourt AMPure XP magnetic beads (Beckman Coulter, Brea, USA). Library size distribution and quality were assessed using the Agilent High Sensitivity DNA kit (Agilent Technologies, USA), and library concentration was determined using the Quant-iT DNA Assay Kit, High Sensitivity (Thermo Fisher Scientific, USA). Thereafter, libraries were pooled in equimolar amounts and diluted to a final concentration of 750 pM. Sequencing of the prepared libraries was performed on a NextSeq 1000 platform (Illumina, San Diego, CA, USA) using the NextSeq 1000/2000 P2 Reagents kit (200 Cycles) v3, supplemented with 2% Phix (Illumina, San Diego, CA, USA) as an internal control.

Primary quality control of reads was performed using the FastQC utility [24]. Removal of low-quality read regions and technical adapters was performed using the Trimmomatic utility [25]; reads were additionally processed with the Cutadapt utility with the parameter --nextseq-trim 20. For quantitative assessment of gene expression levels, the Salmon utility [26] (mapping-based mode) was used. The complete transcript set for the R. norvegicus genome version rn6 from the ENSEMBL database (version 106) was used as the reference transcriptome. When building the reference transcriptome index, the genome nucleotide sequence was used as a decoy sequence to avoid erroneous read assignment to transcripts. Gene expression levels were calculated based on the expression values of individual transcripts of these genes using the R package tximport (ver. 1.37.2) [27].

Visualisation of sample clustering by principal component analysis (PCA) was performed using the R package PCAtools (ver. 2.21.0). The R package edgeR (ver. 3.22) [28] was used to assess differential gene expression between groups, and the glmLRT was chosen as the statistical test. An FDR < 0.05 was considered the criterion for statistically significant changes in gene expression between groups.

### 2.10. Statistical Analysis

All experiments were performed in duplicate at least 4 times on cultures of unrelated cells. Results of the MTT test and morphometric analysis were processed using the Shapiro–Wilk test and the Kruskal–Wallis test with Dunn’s post hoc test, as the data deviated from a normal distribution. Results of calcium and ΔΨm imaging analysis were processed according to the methodology described previously, and statistical analysis was performed using the non-parametric Student’s *t*-test followed by the Mann–Whitney test [23]. Data are presented as violin and box plots indicating medians and interquartile ranges. Differences were considered statistically significant at a confidence level of *p* ≤ 0.05. Statistical data processing was performed using Excel and Prism 10.

## 3. Results

### 3.1. Neuroglial Culture Characteristics

**Figure 1:**
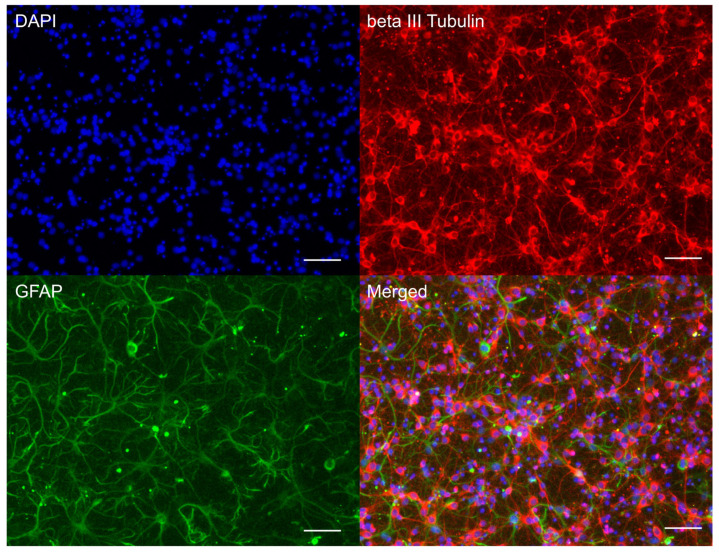
Characterisation of cultured cortical neuroglial cells. Immunophenotyping of neuroglial culture for neuronal beta-III-tubulin (red) and astroglial GFAP (green) markers. Cell nucleus stained by DAPI (blue). Scale bar: 50 µm.

On day 10 in vitro (DIV 10), a mature mixed neuroglial culture, predominantly composed of cortical neurons, had been established. Neurons were morphologically distinguishable from glial cells by their spherical soma and extensive neurites and were immunopositive for the neuronal marker beta-III-tubulin. Glial cells, immunopositive for the astrocytic marker GFAP, exhibited an irregular morphology with processes (Figure 1). This culture type was subsequently utilised for inducing glutamate excitotoxicity and for subsequent measurements of intracellular calcium levels ([Ca^2+^]_i_), mitochondrial membrane potential (Ψm), and transcriptomic analysis.

### 3.2. EV Characteristics

**Figure 2:**
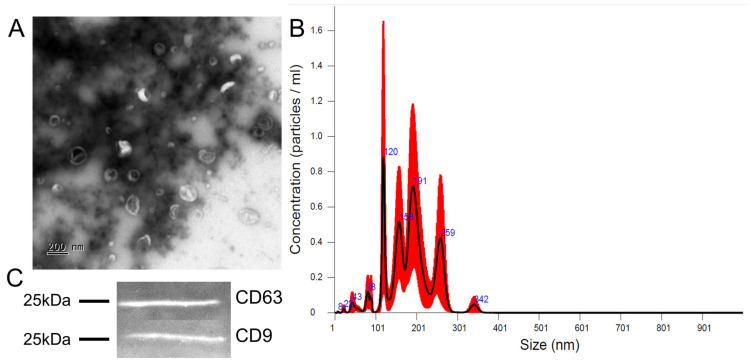
Characterisation of extracellular vesicles released by human GPCs. (**A**) Transmission electron microscopy images, scale bar 200 nm. (**B**) Size distribution chart, black line—mean, red zone—error bars indicate +/−1 standard error of mean (5 repeats), blue number—size of particle (nm) for peak. (**C**) Representative immunoblots showing the presence of tetraspanins CD9 and CD63.

The nanoparticle tracking assay for concentration and size distribution measurements, and transmission electron microscopy for morphological assessment of EV-GPCs were used. Most of the particles fell within a range of 80–300 nm, with four peaks at 120 nm, 158 nm, 191 nm, and 259 nm (Figure 2B). Concentrations of particles produced by GPCs varied slightly—from 4.51 × 10^11^ to 8.37 × 10^11^ particles per ml isolate. As revealed by transmission electron microscopy, most particles in the isolates had cup-shaped morphology characteristic of EVs, opposed to a minor portion of smooth-surfaced, smaller-sized objects of spherical shape (Figure 2A). EV-GPCs were stained for tetraspanins CD9 and CD63, which are specific for extracellular vesicles (Figure 2C).

### 3.3. Proteomic Analysis of EVs

To assess the protein composition of EVs of glial progenitor cells, a proteomic analysis was performed; 1357 EV-GPC proteins were identified and classified according to molecular function, intracellular compartments, biological process, and signalling pathways [29,30,31]. A complete list of detected proteins is available in Supplementary Materials.

#### 3.3.1. Belonging of Proteins to Intracellular Compartments

**Figure 3:**
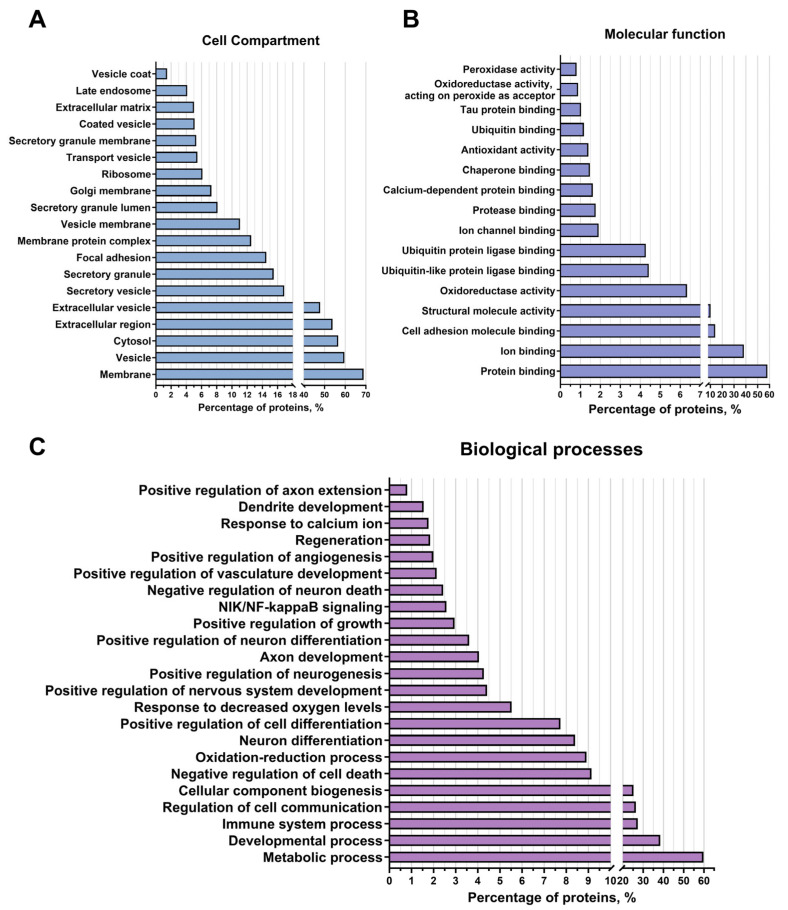
Proteomic analysis of extracellular vesicles from glial progenitor cells. Extracellular vesicle proteins were classified using String 11.8 into cellular component (**A**), molecular function (**B**), and biological processes (**C**).

After localisation (categories of cellular components), 60% of the identified proteins belong to the groups “vesicles” and “extracellular vesicles”, indicating that a preparation enriched with extracellular vesicles was obtained. In this category, proteins belonging to the “secretory granules” (17% of the identified proteins) and “secretory vesicles” (17%) compartments were identified, which may be associated with specialised proteins for extracellular export (Figure 3A). And 69%, 13%, and 3% of the identified proteins belonged to the groups “membrane”, “membrane protein complex”, and “vesicle coat”, respectively. The identified proteins were probably localised in the membranes of the vesicles themselves. Additionally, 56.5% of the proteins were found in the category “cytosol”, indicating that these proteins were localised in the vesicle lumina. In addition, 53%, 15%, and 5% of the identified proteins were assigned to the categories “extracellular region”, “focal adhesion”, and “extracellular matrix”, respectively. Most likely, the proteins of these groups were exposed on the outer surface of the vesicles and were therefore the first to bind to the receptors on the membranes and transmit a signal to the target cells. In addition, 8%, 6.5%, and 3% of the proteins were assigned to the categories “lumen of secretory granules”, “Golgi membrane”, and “late endosome”, respectively. “Late endosome” (especially various Rab family proteins such as Rab7, Rab6a, Rab5 [32], and others) regulated the formation and distribution of intracellular membrane compartments, suggesting pathways of extracellular vesicle formation and intracellular migration through the Golgi apparatus and the late endosomal compartment. It was also found that 7% of the identified proteins belonged to the category “Ribosome”, suggesting the presence of ribosomal proteins in the obtained preparation.

#### 3.3.2. Participation of Cargo Proteins in Biological Processes

According to the categories of biological processes, 60% of the analysed proteins were involved in metabolic processes, 38% in developmental processes, 27.5% in processes of the immune system, 25% in the formation of cellular components, and 9% in cellular communication. Among the identified proteins belonging to the “immune system processes” group, proteins related to lymphocyte migration were found, such as Serine/threonine-protein kinase 10, *STK10*, also known as LOK, and proteins [33] that have an anti-inflammatory effect, such as TOLLIP, an inhibitor of Toll-like receptors [34], CD59 Molecule, also known as MACIF, an inhibitor of the complement system [35], and MDA-9/syntenin, which also has an anti-inflammatory effect [36]. In addition, many proteins of the integrin family have been found in the vesicles, such as proteins of genes *ITGA3*, *ITGB1* [37], and others, which regulate the permeability of blood vessels and the attraction of leukocytes, which may indicate their involvement in the control of inflammatory processes by regulating the infiltration of blood cells into brain tissue. The protein MVP (major vault protein) has also been found to help reduce inflammatory processes by suppressing the proliferative activity of macrophages in models of atherosclerosis and osteoporosis [38]. In addition, a protein with anti-inflammatory properties, annexin A2 [39], has been found to be a modulator of autophagy in cells and to maintain the integrity of blood vessels, thereby reducing leukocyte infiltration (Figure 3C). Furthermore, the detected proteins were distributed among the BP groups “oxidation-reduction processes” (8% of the identified proteins) and “response to decreased oxygen levels” (6%). Among these proteins responsible for maintaining the redox balance of the intracellular environment and activating the response to hypoxia are Ferritin with all its subunits [40], various peroxiredoxins (peroxiredoxin 1, peroxiredoxin 6, peroxiredoxin 4, and others [41]), which are responsible for the oxidation/reduction of lipids and proteins, and the protein Hypoxia Up-Regulated 1 [42] (known as *ORP150*), which is the main activator of the response pathway to decrease oxygen levels. In addition, 9% of the identified proteins were assigned to the “negative regulation of cell death” group, while 4% of the proteins were assigned to the “negative regulation of neuron death” group. These included proteins that directly negatively regulate cell death (e.g., all subunits of the 14-3-3 protein, which is a direct inhibitor of the proapoptotic protein Bad [43]); and indirectly, e.g., the protein of gene *DDB1* [44], which is involved in DNA repair, endoplasmin; which is involved in the transfer of misfolded proteins to the ERAD complex [45]; the 60 kDa heat shock protein [46], which is responsible for the correct assembly of unfolded polypeptides formed under stress conditions in the mitochondrial matrix; and others. In addition, 2.5% of the identified proteins were classified in the BP category “NIK-Nf-kappaB signalling”. These included the following proteins: subunits of the Nf-kappaB complex and activators of NIK-Nf-kappaB signalling (e.g., DEAD-Box Helicase 1 [47]). These data suggest that extracellular vesicle proteins activate cell survival pathways and stabilise intracellular homeostasis.

In addition, 8% of the proteins were assigned to both the BP category “neuronal differentiation” and the category “positive regulation of cellular differentiation”. And 4% of the proteins were assigned to the groups “positive regulation of neuronal differentiation” and “positive regulation of nervous system development”, including, for example, the protein MMP-14 [48], which is involved in the migration of neural stem cells, as well as PEDF [49] and Copine 1 [50], which are involved in the proliferation and differentiation of neuronal progenitor cells. A small proportion of the proteins belonged to the BP “positive regulation of neurogenesis” (5%), including proteins such as Drebrin or the protein of gene *DOCK10* [51,52], which are responsible for the development and stability of the dendritic tree and dendritic spines, as well as proteins such as Attractin, which plays a crucial role in the myelination of the central nervous system [53], and Plexin-B2 or MANF [54,55], which are responsible for the regeneration and development of the nervous system. An additional 4% of the proteins were assigned to the BP “axon development” and 1% to the BP “axon elongation”, including the following proteins: Dynamin-2 [56], Neuropilin-1 [57], and others, which may indicate that extracellular vesicles also contribute to neuroplasticity development and neural tissue regeneration. Additionally, 3% of the proteins were categorised as “positive growth regulation”, and 2% of the proteins were identified as proteins in the “vascular development” and “positive regulation of angiogenesis” categories, including proteins such as Myeloid-derived growth factor or Endoglin [58,59], and Lactadherin or Heparan sulphate proteoglycan 2 proteins [60], which are involved in vascular growth, migration, and proliferation of endothelial cells, suggesting that vesicles may be involved in the activation and regulation of vascular growth in damaged tissue (Figure 3C).

#### 3.3.3. Molecular Functions of EV-GPC Cargo Proteins

The main category was “protein binding” (about 68% of identified vesicle proteins), which included categories such as “cell adhesion protein binding” (14%), “ubiquitin-like protein ligase binding” and “ubiquitin protein ligase binding” (4% both), “protease binding” and “chaperone binding” (1.5% both), “ubiquitin binding” and “tau protein binding” (1% both). Proteins with various activities were also distinguished, such as “structural molecule activity” (10%), “oxidoreductase activity” (6%) (e.g., Superoxide dismutase 2 [61], Glutathione peroxidase 4, and peroxidase 8 [62]), “antioxidant activity” (1.5%) (the already mentioned peroxiredoxins, as well as Thioredoxin), and “peroxidase activity” (1%), indicating the role of extracellular vesicles in cellular communication, signal transduction, proper protein compaction, and cell survival (Figure 3B).

#### 3.3.4. Activated Signalling Pathways by Protein Cargo of EV-GPCs

**Figure 4:**
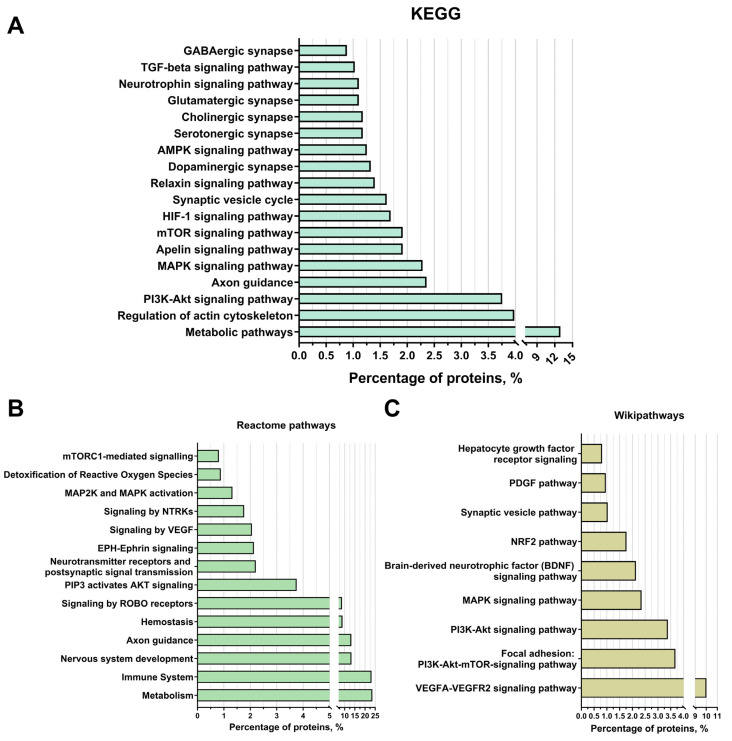
Proteomic analysis of extracellular vesicles from glial progenitor cells. Extracellular vesicle proteins were classified using String 11.8 into KEGG (**A**), Reactome pathways (**B**), and WikiPathways (**C**).

Among the established KEGG signalling pathways, the “PI3K-Akt pathway” is the most interesting as it is one of the most important signalling pathways promoting neuroglial cell survival in different areas of the brain under conditions of oxygen and glucose deprivation and neurotoxicity, as confirmed by numerous studies. In addition, this signalling pathway is involved in cell cycle control, promotes proliferation, and regulates angiogenesis. Among the proteins involved in this signalling pathway, subunits of proteins of the integrin-alpha and integrin-beta receptors have been identified that activate this pathway via focal contacts, for example, via the various laminins and thrombospondins (e.g., Laminin beta-1 and Thrombospondin 1). CDC37 proteins [63], as well as both subunits of HSP90 [64], which are part of the AKT activator complex, all subunits of the Ras protein, which also activates PI3K, the Jak protein [65], which indicates a cytokine signalling pathway for PI3K-Akt activation, and the beta/gamma subunit of the G protein (Gβγ), which activates PI3K by mediating chemokine signalling [66], were also identified. Furthermore, 14-3-3 protein subunits (YWHAB, YWHAE, YWHAH, YWHAG, YWHAQ, YWHAZ) were identified by proteomic analysis. The 14-3-3 protein family mediates signalling transduction by binding to phosphoserine-containing proteins. They are adaptor proteins involved in the regulation of a variety of general and specialised signalling pathways, in particular in the positive regulation of the cell cycle and cell survival by inhibiting proapoptotic proteins (Figure 4A). Based on the KEGG data, an “axon guidance” category was identified. Identified proteins included: Plexin-A2 and Reticulon-4, also known as Nogo, which stop axon–axon interactions and promote neurite branching [67,68], or Ephrin type-B receptor 2 and Ephrin type-B receptor 3 [69], which are also involved in axon guidance via ephrin signalling. Moreover, proteins associated with the ROBO signalling pathway (the ROBO1 gene protein) were found to regulate many functions, but also axonal cone growth and navigation as well as angiogenesis [70] (Figure 4A).

The signalling pathways “MAPK signalling” (2.3% of the proteins found, in particular both subunits of the Erk protein) and “mTOR signalling” (2% of the proteins, in particular the mTOR protein itself, as well as several subunits of the Ragulator protein complex, which is an activator of mTOR), “HIF-1 signalling” (2%), “Apelin signalling” (2%), “Relaxin signalling” (2%), “AMPK signalling” (2%), neurotrophin signalling (1%), and “TGF-beta signalling” (1%). In addition, according to the KEGG data, the following groups were identified: “synaptic vesicle cycle” (1.5% of the detected proteins, such as syntaxins and synaptotagmins) and categories related to different types of synapses (“dopaminergic synapse” (about 2%), “serotonergic synapse” (about 2%), “cholinergic synapse” (about 2%), “glutamatergic synapse” (1.5%), and “GABAergic synapse” (about 1%)). Among the proteins assigned to these categories, the proteins of the Gi/o family and the PP1-1 protein, which are responsible for synaptic plasticity, should be emphasised. In addition, for the categories “glutamatergic synapse” and “GABAergic synapse”, the protein SNAT8 was identified, which is responsible for the transport of glutamine from glial cells into the neuron and is thus involved in the metabolism of glutamate and GABA (Figure 4A).

The main categories of the Reactome pathways were as follows: “metabolism” (24% of the detected proteins), “immune system” (23%), “nervous system development” (13%), “axon navigation” (about 13% of proteins), “hemostasis” (9%), “ROBO receptor signalling” (about 9% of proteins), “PIP3-activated AKT signalling” (about 4% of proteins), “neurotransmitter receptors and postsynaptic signalling” (2%), “EPH-Ephrin signalling” (2%), “VEGF signalling” (2%), “NTRKs signalling” (1.5%), “MAP2K and MAPK activation” (1%), “reactive oxygen species detoxification” (1% of proteins), and “mTORC1-mediated signalling” (1% of proteins), which is consistent with the previously obtained data from other methods of analysis (Figure 4B).

Among the groups identified using WikiPathways were “VEGFA-VEGFR2 signalling pathway” (10% of proteins), “focal adhesion: PI3K-Akt-mTOR signalling pathway” (4% of identified proteins), “PI3K-Akt signalling pathway” (3%), “MAPK signalling pathway” and “brain-derived neurotrophic factor (BDNF) signalling pathway” (2% each), “NRF2 pathway” (2%), “synaptic vesicle pathway” (1%), “PDGF pathway” (1% of proteins, in particular the PDGFRB receptor subunit for this factor), and “hepatocyte growth factor receptor signalling pathway” (1%), which is also consistent with the data on the putative mechanisms of actions of extracellular vesicles associated with neuroprotection, nervous system regeneration, and angiogenesis (Figure 4C).

### 3.4. Modelling Glutamate Excitotoxicity and the Neuroprotective Effect of Vesicles

**Figure 5:**
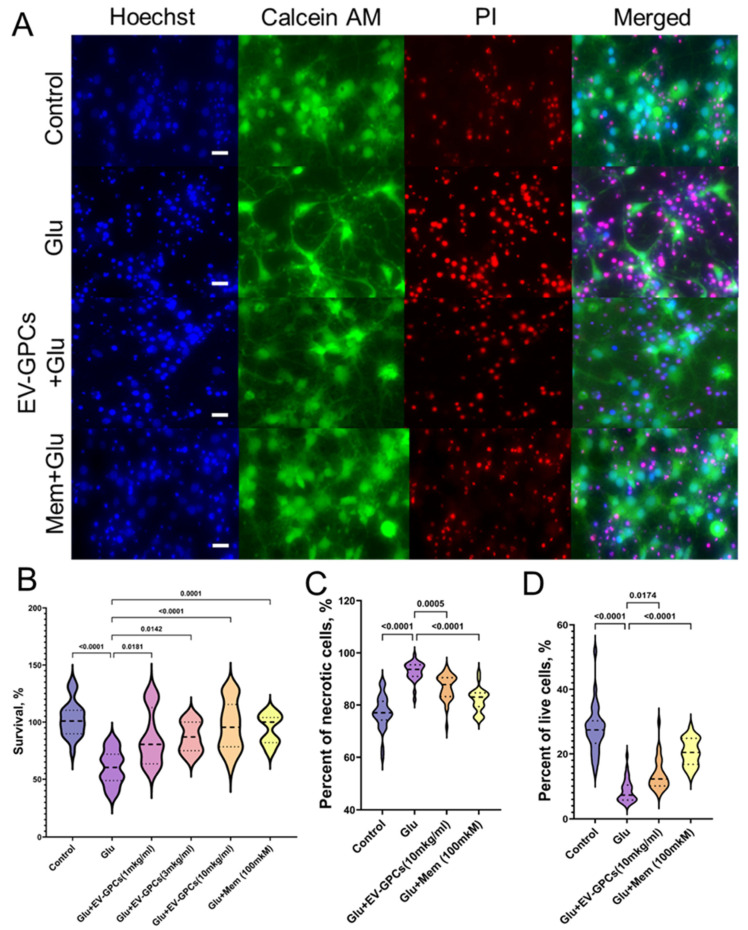
Neuroprotective properties of EV-GPCs in a model of glutamate excitotoxicity. Cultures were treated with 100 µM glutamate (Glu) to induce excitotoxicity. Experimental groups were co-treated with EV-GPCs or 100 µM memantine (Mem) as a positive control. (**A**) Representative fluorescence micrographs of cortical cultures stained with Hoechst 33342 (nuclei, blue), Calcein AM (viable cells, green), and propidium iodide (PI) (necrotic nuclei, red). Scale bar: 20 µm. (**B**) Viability of neuronal cultures assessed by MTT assay. Data are presented as a percentage of survival relative to the intact control group. (**C**) Percentage of necrotic cells. (**D**) Percentage of viable cells. All data were analysed by the Kruskal–Wallis test with Dunn’s post hoc test. Results are presented as violin plots showing medians and interquartile ranges.

The evaluation of neuroprotective efficacy involved survival analysis and morphometric assessment of karyonecrosis. The induction of glutamate excitotoxicity reduced the viability of cortical cells by 39.6 ± 5.93% compared with the intact control group (*p* < 0.0001). Treatment with EV-GPCs promoted a dose-dependent increase in neuronal survival. At a concentration of 1 μg/mL, the survival rate of neuroglial cells was significantly increased by 20.0 ± 2.91% (*p* = 0.0181). A dose of 3 μg/mL increased survival by 30.0 ± 2.98% (*p* = 0.0142), while a dose of 10 μg/mL restored survival to levels observed in the intact control (*p* < 0.0001). The efficacy at the highest dose was comparable to that of the positive control, memantine (*p* = 0.0001) (Figure 5B).

On morphometric analysis, the control group had 28% Calcein+ cells (Q25 = 23.28-Q75 = 30.27; n = 45,982, where n is the number of nuclei stained with Hoechst 33342), while the percentage of cells with karyonecrosis was 77% (Q25 = 74.25-Q75 = 81.45; n = 45,982), consistent with partial cell death during planting of the primary culture. Glutamate excitotoxicity increased the percentage of necrotic cells in the culture to 94% (Q25 = 90.95-Q75 = 95.41; n = 48,735; *p* < 0.0001) and decreased the percentage of viable cells to 6% (Q25 = 5.778-Q75 = 10.34; n = 48,735; *p* < 0.0001). The presence of memantine as a positive control increased the number of viable cells to 21% (Q25 = 16.76-Q75 = 24.81; n = 46,434; *p* < 0.0001) and reduced the percentage of necrotic cells to 83% (Q25 = 79.40-Q75 = 84.66; n = 46,434; *p* < 0.0001). The addition of 10 μg/mL EV-GPCs also increased the percentage of Calcein+ cells to 15% (Q25 = 10.08-Q75 = 16.19; n = 45,100; *p* = 0.0174) and decreased the percentage of necrosis in the culture to 85% (Q25 = 83.23-Q75 = 90.47; n = 45,100; *p* = 0.0005) (Figure 5A,C,D).

### 3.5. Measurement of Intracellular Ca^2+^ Concentration ([Ca^2+^]_i_) and Mitochondrial Transmembrane Potential (ΔΨm)

**Figure 6:**
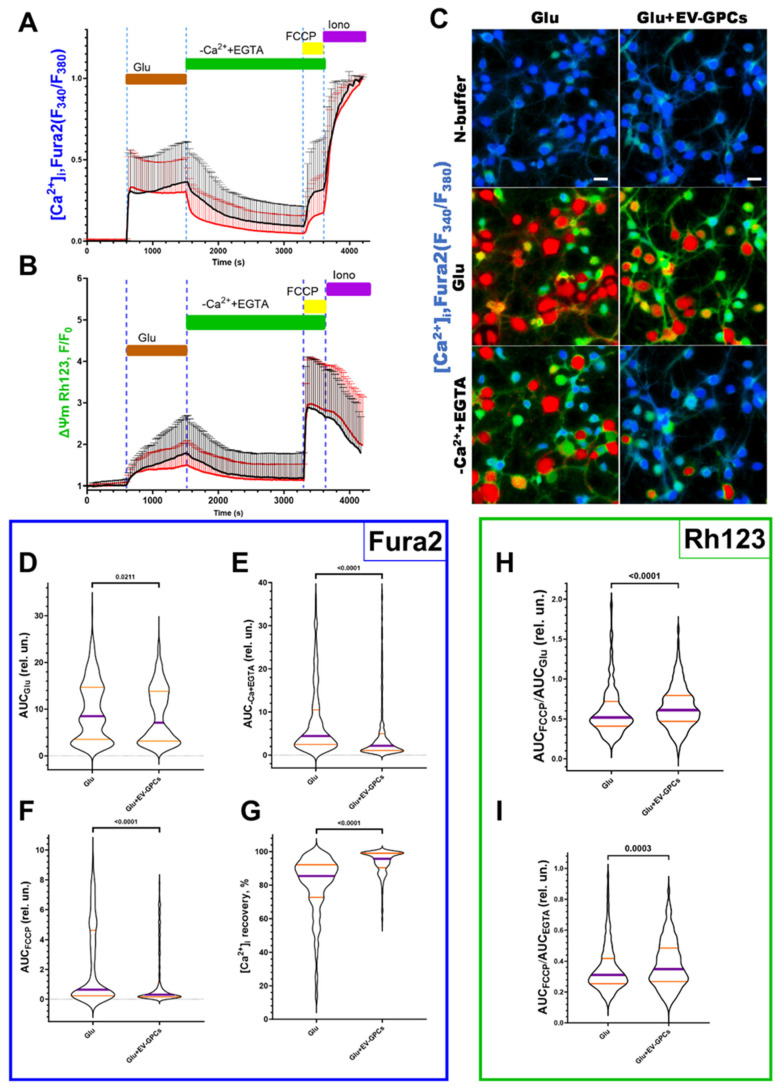
Comparison of the parameters of dynamic changes in calcium homeostasis and mitochondrial potential in the modelling of glutamate excitotoxicity in the glutamate group (Glu, 100 μM) and with the supplement of glutamate (100 μM) and EV-GPCs (10 μg/mL) 24 h before the experiment. [Ca^2+^]_i_ and Ψm were measured using Fura2 and Rh123, respectively. (**A**) average curves of [Ca^2+^]_i_ changes in the glutamate group (black line, n = 619 cells) and the group with the addition of glutamate and EV-GPCs (red line, n = 621 cells). (**B**) average curves of the dynamics of Ψm changes in the glutamate group and the group with the addition of glutamate and EV-GPCs; data are presented as means and standard deviations. (**C**) representative images of cortical neuron cultures before addition of glutamate, after 15 min of incubation with glutamate, and after removal of glutamate and replacement of the solution with a calcium-free solution; colour represents intensity of Fura2 fluorescence and ranges from blue (low-intensity) to red (high-intensity); scale bar, 10 µm. (**D**–**I**) Analysis of [Ca^2+^]_i_ and Ψm changes by calculating the areas under the curves (AUC) in different periods. (**D**) effect of EV-GPCs on AUC values during glutamate exposure (15 min) (AUC_Glu_), (**E**) after glutamate removal in nominally calcium-free buffer (20 min) (AUC-_Ca+EGTA_), (**F**) after adding of FCCP protonophore (5 min) (AUC_FCCP_), and (**G**) % recovery of baseline [Ca^2+^]_i_. (**H**) degree of Ψm reduction expressed as AUC_FCCP_/AUC_Glu_, (**I**) degree of Ψm recovery expressed as AUC_FCCP_/AUC_EGTA_. Data were analysed by nonparametric unpaired *t*-test followed by Mann–Whitney test and presented as median and interquartile range.

The addition of glutamate (Glu) at a concentration of 100 μM (15 min) significantly increased the cytoplasmic calcium level [Ca^2+^]_i_ and simultaneously caused a decrease in Ψm in all cells (619 cells for glutamate group (Glu), 621 cells for group with glutamate and EV-GPC addition) (Figure 6A,B). Figure 6C shows ratiometric fluorescence images of a Fura2-loaded cortical neuron culture demonstrating changes in Ca^2+^ levels after glutamate exposure and glutamate removal by buffer exchange with a calcium-free buffer containing EGTA (warmer colour corresponds to higher [Ca^2+^]_i_ levels). To evaluate the intracellular changes in the studied groups, various parameters were analysed as previously described [71]. Briefly, the value of changes in [Ca^2+^]_i_ and Ψm in individual cells was assessed by the change in the area under the curve (AUC) of fluorescence of the corresponding Fura2 and Rh123 dyes.

The intensity of calcium ion accumulation in the cytoplasm of the cells against the background of glutamate action, estimated by the AUC_Glu_ parameter, decreased significantly (*p* = 0.0211) from the glutamate group values of 8.515 relative units (Q25 = 3.531-Q75 = 14.66; n = 619) to 7.093 relative units (Q25 = 3.141-Q75 = 13.84; n = 621) against the background of pre-incubation of the cells with EV-GPCs (Figure 6D). The removal of glutamate in a nominally calcium-free buffer led to a decrease in [Ca^2+^]_i_ in the analysed groups. At the same time, the value of the parameter AUC_Ca2++EGTA_ decreased significantly to 2.155 relative units (Q25 = 1.038-Q75 = 4.956; n = 621) in the group with cell pre-incubation with EV-GPCs compared with the glutamate group’s 4.421 relative units (Q25 = 2.431-Q75 = 10.47; n = 619), indicating a more intense removal of Ca^2+^ ions from the cytoplasm of cortical neurons in the post-glutamate period (Figure 6E). This is consistent with a smaller increase in [Ca^2+^]_i_ after the addition of FCCP (Figure 6A). The percentage of recovery of [Ca^2+^]_i_ to baseline during this period increased significantly to 95.71% (Q25 = 90.38-Q75 = 99.05; n = 621) in the presence of EV-GPCs compared with 85.40% (Q25 = 72.67-Q75 = 92.13; n = 619) for the glutamate group (Figure 6G).

Mitochondria are the most important intracellular stores of calcium ions in neurons and astrocytes and protect the cytosol and nucleoplasm from Ca^2+^ overload [72]. Upon depolarisation of the inner mitochondrial membrane by addition of the protonophore FCCP (1 μM), electrophoretic retention of Ca^2+^ in the mitochondrial matrix ceases and Ca^2+^ is released into the cytoplasm, causing a rapid increase in [Ca^2+^]_i_ (Figure 6A). The addition of EV-GPCs resulted in less Ca^2+^ release from the mitochondria (Figure 6F). The AUC_FCCP_ value decreased significantly from 0.6466 relative units (Q25 = 0.2265-Q75 = 4.607; n = 619) in the glutamate group to 0.3072 relative units (Q25 = 0.1522-Q75 = 1.101; n = 621).

To evaluate the changes in Ψm before and after exposure, the signal intensity of the voltage-sensitive mitochondrial probe Rh123 was measured under the effect of the protonophore FCCP and glutamate. AUC_Glu_ and AUC_Ca2++EGTA_ were calculated from the fluorescence curves of the Rh123 probe for individual cells as described above. The addition of Glu (100 μM) resulted in an increase in the Rh123 signal, indicating mitochondrial depolarisation (Figure 6B). The addition of FCCP resulted in maximal signal amplification as Rh123 dye accumulated in the cytoplasm following a rapid decrease in Ψm. To determine the ability of the cells to maintain Ψm, the ratios AUC_FCCP_/AUC_Glu_ and AUC_FCCP_/AUC_Ca2++EGTA_ were measured. The higher the AUC_FCCP_ ratio in the investigated stages, the more Rh123 dye accumulated in the mitochondria, as these were less depolarised. The addition of EV-GPCs led to a significant and reliable increase in the AUC_FCCP_/AUC_Glu_ ratio to 0.6098 relative units (Q25 = 0.4687-Q75 = 0.7943; n = 621) compared with the glutamate group’s 0.5176 relative units (Q25 = 0.4091-Q75 = 0.7196; n = 619) (Figure 6H). The AUC_FCCP_/AUC_Ca2++EGTA_ ratio was also higher in the EV-GPC background: 0.419 relative units (Q25 = 0.309-Q75 = 0.587; n = 434) compared with the glutamate group’s 0.363 relative units (Q25 = 0.280-Q75 = 0.510; n = 485) (Figure 6I).

At the end of the experiment, ionomycin (Iono, 2 μM) with Ca^2+^ 5 mM was added to achieve the maximum concentration of Ca^2+^ in the cells.

### 3.6. The Role of the PI3K-Akt Pathway in Neuron Survival

**Figure 7:**
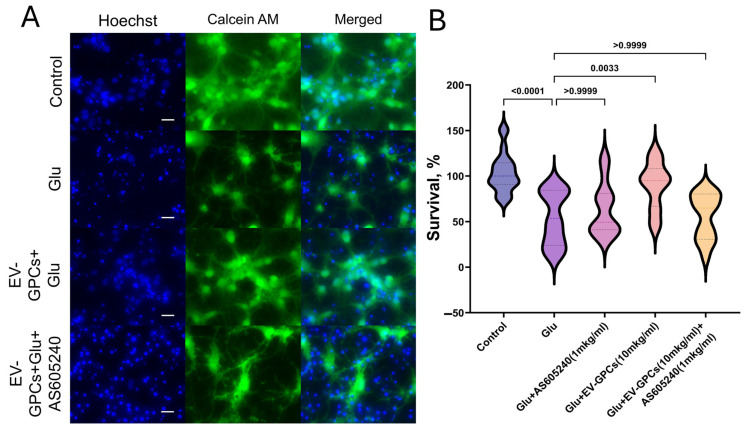
Pharmacological inhibition of the PI3K signalling pathway attenuates the neuroprotective efficacy of EV-GPCs. (**A**) Representative micrographs of cortical cultures stained with Hoechst 33342 (nuclei, blue) and Calcein AM (viable cells, green). Scale bar, 20 μm. (**B**) Assessment of neuronal culture viability by MTT assay. Data are normalised and presented as a percentage of survival relative to the untreated control group. Experimental conditions: glutamate (Glu, 100 μM), EV-GPCs (EV-GPCs, 10 μg/mL), and the PI3K inhibitor AS605240 (AS605240 1 μg/mL). Statistical analysis was performed using the Kruskal–Wallis test followed by Dunn’s post hoc test for multiple comparisons. Results are presented as violin plots depicting median values and interquartile ranges.

As in the previous experiment, the addition of glutamate reduced the survival rate of neuroglial cultures by 39.6 ± 5.93% compared with the control group, and the addition of 10 μg/mL EV-GPCs contributed to an increase in viable neuroglial cells to control levels (*p* < 0.001). The simultaneous administration of 1 μg/mL AS605240 and glutamate had no effect on cell survival. However, the simultaneous addition of the inhibitor and the EV-GPC drug (10 μg/mL) resulted in a reliable decrease in survival to values in the group with glutamate excitotoxicity. (Figure 7A,B).

### 3.7. The Effect of EV-GPCs on the Gene Expression Profile of Neuroglial Cultures. Comparison of Gene Expression in Intact Cells with Neuronal Cultures Pretreated by EV-GPCs (IC_vs._EV)

To investigate the changes in the gene expression profile of the neuroglia culture after addition of EV-GPCs, mRNA sequencing analysis was performed. In the IC_vs._EV comparison group, 52 differentially expressed genes (DEGs) were significantly up-regulated, while 181 DEGs were significantly down-regulated (*p* < 0.05 and |FC| < 1.5). The complete lists of DEGs, results of clustering (Supplementary Materials Figure S1), and the heatmap (Supplementary Materials Figure S2) can be found in Supplementary Materials. The volcano plot (Supplementary Materials Figure S3) demonstrates the differences in differential gene expression between groups and illustrates the molecular changes induced by the addition of EV-GPCs. The most striking DEGs are shown in Heatmap 8A.

**Figure 8:**
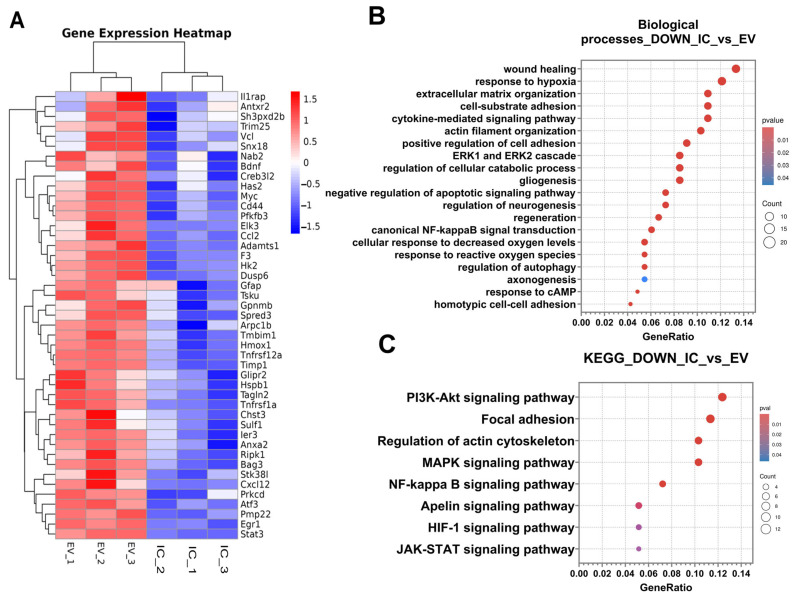
Transcriptomic profiling of neuroglial cultures following EV-GPC treatment. (**A**)—heatmaps of differentially expressed genes (DEGs) were obtained using pheatmap R package (version 4.5.2). Each row represents a gene and each column corresponds to an individual sample, sample groups indicated by column annotations. Log2-transformed gene expression values were row-scaled; hierarchical clustering was performed for both rows (genes) and columns (samples) using Euclidean distance and the complete clustering method. The colour scale represents scaled expression values and ranges from blue (down-regulated) to red (up-regulated); (**B**)—results of classification of down-regulated DEGs using the Gene Ontology Biological Processes database. The X-axis shows the ratio of pathway DEGs to the total number of DEGs, and the Y-axis shows the selected biological processes; (**C**)—results of classification of down-regulated DEGs using the KEGG database. The X-axis shows the ratio of pathway DEGs to the total number of DEGs, and the Y-axis shows the selected signalling pathways.

Analysis of differentially expressed genes (DEGs) using the Gene Ontology (GO) Biological Process database revealed a significant enrichment of down-regulated DEGs in key biological pathways. Supplementary Materials Table S1 presents the most significant DEGs belonging to the selected biological pathways (Supplementary Materials Table S1). The process categories containing the highest number of DEGs were wound healing, response to hypoxia, and the cytokine-mediated signalling pathway (Figure 8B).

It was found that down-regulated DEGs were reliably grouped into biological pathways responsible for changes in the cytoskeleton, extracellular matrix, and cell adhesion: extracellular matrix organisation; cell-substrate adhesion; actin filament organisation; positive regulation of cell adhesion; homotypic cell–cell adhesion, and axonogenesis (Figure 8B).

Down-regulated DEGs were also significantly associated with alterations in canonical signalling pathways, including the ERK1 and ERK2 cascade, canonical NF-κB signal transduction, response to cAMP, response to reactive oxygen species, and cellular response to decreased oxygen levels (Figure 8B). This pattern suggests the activation of sequential signal transduction events that promote adaptive cellular responses aimed at maintaining homeostasis and ensuring cell survival under exogenous stress.

Furthermore, several DEGs were classified into categories related to regeneration and neurogenesis, such as regulation of cellular catabolic processes, regulation of neurogenesis, gliogenesis, negative regulation of apoptotic signalling, regulation of autophagy, and regeneration (Figure 8B).

To further elucidate the underlying mechanisms and activated intracellular cascades, down-regulated DEGs were analysed for enrichment in signalling pathways using the KEGG database [23,24,25]. This analysis identified several key pathways: the PI3K-Akt signalling pathway, focal adhesion, regulation of actin cytoskeleton, MAPK signalling pathway, NF-κB signalling pathway, Apelin signalling pathway, HIF-1 signalling pathway, and JAK-STAT signalling pathway (Figure 8C). Most notable were the PI3K-Akt, NF-κB, MAPK, and HIF-1 signalling pathways, whose activation is directly implicated in pro-survival mechanisms and the maintenance of cellular homeostasis.

Gene set enrichment analysis (GSEA) was performed to assess coordinated variations in the expression of pre-defined gene sets. This method evaluates whether defined sets of genes exhibit statistically significant, concordant differences in expression between two biological states, rather than focusing on individual genes. Supplementary Materials Figure S4 presents the results obtained using the Hallmark gene set collection, illustrating gene sets that are activated in the control group (normalised enrichment score (NES) > 0) and in the group treated with extracellular vesicles (NES < 0), with a significance threshold of *p*-value < 0.05.

### 3.8. Assessment of Gene Expression in Neuroglial Cultures Under Glutamate-Induced Excitotoxicity

The subsequent phase of the experiment involved transcriptomic analysis of neuroglial cultures following glutamate excitotoxicity. Three comparison groups were established as follows: control group without glutamate addition versus cultures incubated with glutamate (IC_vs._GL), cultures incubated with glutamate and memantine versus cultures incubated with glutamate (GL_Mem_vs._GL), and control group without glutamate addition versus cultures incubated with glutamate and memantine (IC_vs._GL_Mem), in which subsequent differential gene expression (DEG) analysis and classification by biological processes and signalling pathways were performed.

DEG analysis in the IC_vs._GL comparison group revealed that 969 genes were significantly up-regulated, while 300 genes were significantly down-regulated (*p*-value < 0.05 and |FC| > 1.5). In the GL_Mem_vs._GL comparison group, 797 genes were identified as significantly up-regulated and 300 as down-regulated (*p*-value < 0.05 and |FC| > 1.5). No significantly differentially expressed genes were detected in the IC_vs._GL_Mem comparison group. Complete lists of DEGs and results of clustering (Supplementary Materials Figure S5) and corresponding heatmaps (Supplementary Materials Figures S6 and S7) are provided in Supplementary Materials. The volcano plots presented in Supplementary Materials Figure S8A,B illustrate the differences in gene expression between the groups, highlighting the distinct molecular alterations induced by glutamate and memantine treatment.

**Figure 9:**
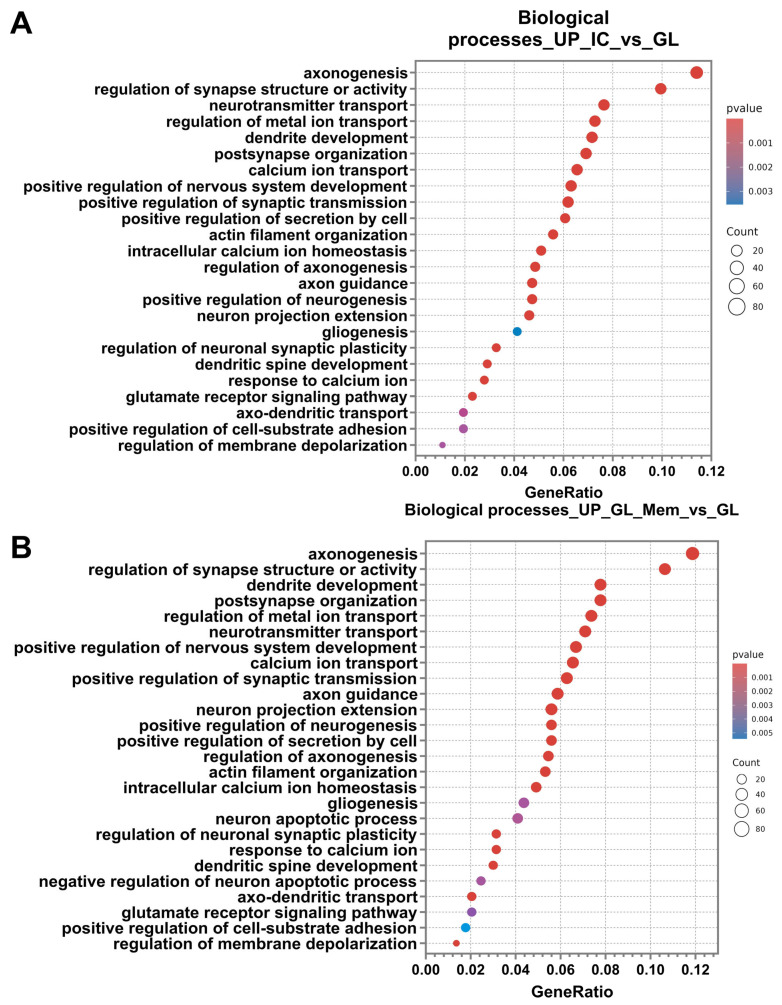
Differential gene expression analysis for the IC_vs._GL and GL_Mem_vs._GL comparison groups. Results of the classification of up-regulated DEGs of the comparison groups IC_vs._GL (**A**), GL_Mem_vs._GL (**B**) using the Gene Ontology Biological Processes database. The X-axis shows the ratio of pathway DEGs to the total number of DEGs, and the Y-axis shows the selected biological processes.

Analysis of differentially expressed genes (DEGs) using the Gene Ontology database revealed a significant enrichment of up-regulated genes in specific biological processes for both the “IC_vs._GL” and “GL_Mem_vs._GL” comparison groups. Subsequent analysis demonstrated that the categories of biological processes identified in the “IC_vs._GL” comparison group exhibited 80% similarity with the up-regulated pathway categories detected in the “GL_Mem_vs._GL” comparison group (Figure 9A,B). The most significant up-regulated DEGs associated with these selected biological pathways are presented in Supplementary Materials Table S2.

Among the most significantly enriched up-regulated categories, key biological pathways associated with synaptic function, axonal outgrowth, and dendritic arborisation were identified. These included axonogenesis, regulation of synapse structure or activity, dendrite development, and neurotransmitter transport.

The most highly activated biological processes can be categorised into several functional groups: regulation of neuronal synapse activity, including post-synapse organisation, positive regulation of synaptic transmission, regulation of neuronal synaptic plasticity, glutamate receptor signalling pathway, and regulation of membrane depolarisation; Maintenance of homeostasis and ion concentration regulation, encompasses regulation of metal ion transport, calcium ion transport, positive regulation of secretion by cell, intracellular calcium ion homeostasis, response to calcium ion, and positive regulation of cell-substrate adhesion; neural tissue development and neurogenesis, comprising positive regulation of nervous system development, positive regulation of neurogenesis, gliogenesis; axonal and dendritic growth pathways, including axon guidance, neuron projection extension, regulation of axonogenesis, actin filament organisation, dendritic spine development, and axo-dendritic transport.

However, DEG analysis in the GL_Mem_vs._GL comparison group revealed the activation of pathways associated with the regulation of apoptosis, which were not detected in the IC_vs._GL group (Figure 9B). Specifically, the “neuron apoptotic process” was identified, alongside genes negatively regulating apoptosis. This observation may be attributed to the partial toxic effect of glutamate supplementation and the subsequent activation of compensatory cellular mechanisms to protect against apoptotic cell death.

**Figure 10:**
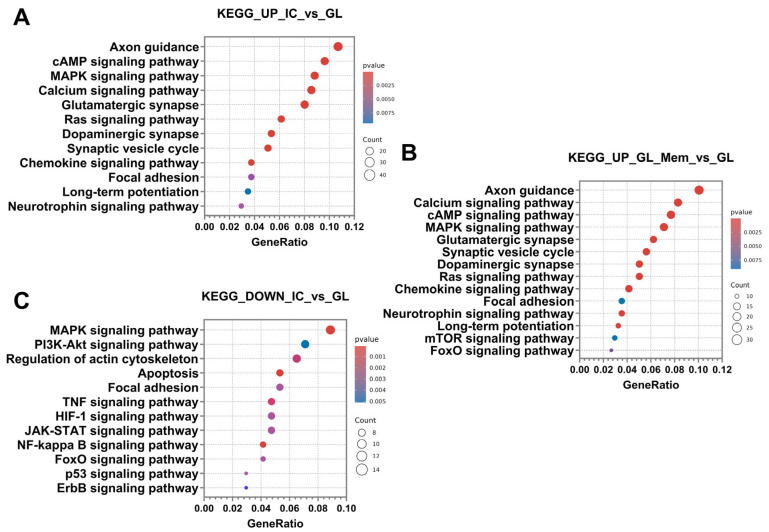
Results of the classification of up-regulated DEGs of comparison groups IC_vs._GL (**A**), GL_Mem_vs._GL (**B**) and down-regulated DEGs of comparison groups IC_vs._GL (**C**) using the KEGG database. The X-axis shows the ratio of pathway DEGs to the total number of DEGs, and the Y-axis shows the selected signalling pathways.

Furthermore, up-regulated DEGs were classified into signalling pathways using the KEGG database. Subsequent analysis revealed that the categories of signalling pathways identified in the “IC_vs._GL” comparison group exhibited 80% similarity with the pathway categories detected in the “GL_Mem_vs._GL” comparison group (Figure 10A,B). DEGs were significantly enriched in the following pathways: axon guidance, calcium signalling pathway, cAMP signalling pathway, MAPK signalling pathway, glutamatergic synapse, synaptic vesicle cycle, dopaminergic synapse, Ras signalling pathway, chemokine signalling pathway, focal adhesion, neurotrophin signalling pathway, and long-term potentiation.

Furthermore, analysis of up-regulated DEGs in the GL_Mem_vs._GL comparison group revealed significant enrichment of pathways associated with the induction of autophagy. This finding may again be attributed to the partial intracellular influx of glutamate and its subsequent toxic impact on neuronal function. Specifically, the mTOR signalling pathway and FoxO signalling pathway were identified as significantly activated (Figure 10B).

**Figure 11:**
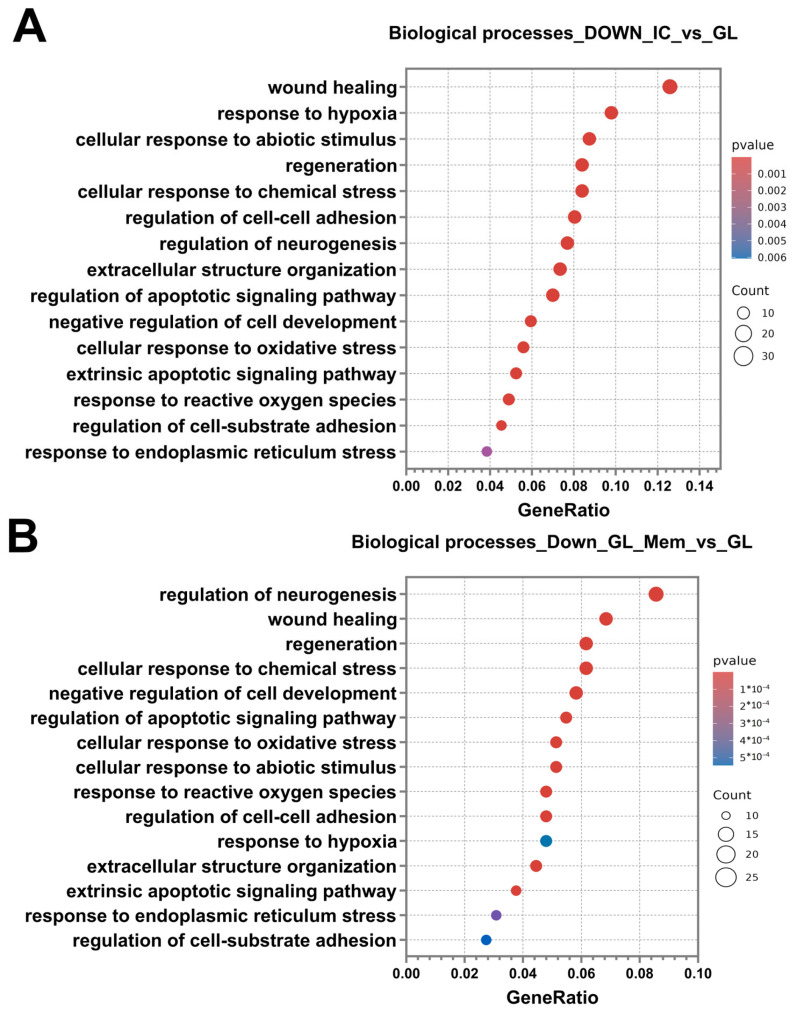
Differential gene expression analysis for the IC_vs._GL and GL_Mem_vs._GL comparison groups. Results of the classification of down-regulated DEGs of the comparison groups IC_vs._GL (**A**), GL_Mem_vs._GL (**B**) using the Gene Ontology Biological Processes database. The X-axis shows the ratio of pathway DEGs to the total number of DEGs, and the Y-axis shows the selected biological processes.

Following the analysis and classification of up-regulated DEGs in the IC_vs._GL and GL_Mem_vs._GL groups, the down-regulated DEGs were analysed. The number of down-regulated DEGs was 300 in both the IC_vs._GL and GL_Mem_vs._GL comparison groups. Subsequent analysis revealed that the categories of biological processes identified in the IC_vs._GL group exhibited 76% similarity with the pathway categories detected in the GL_Mem_vs._GL group (Figure 11A,B). Furthermore, down-regulated DEGs in the GL_Mem_vs._GL group did not show significant enrichment for any specific signalling pathways in the KEGG database analysis. The most significant down-regulated DEGs associated with the selected biological processes are presented in Supplementary Materials Table S2.

Classification of genes by biological processes using the Gene Ontology database revealed categories associated with the activation of protective cellular pathways against glutamate toxicity. These included response to hypoxia, cellular response to abiotic stimulus, cellular response to chemical stress, negative regulation of cell development, cellular response to oxidative stress, response to reactive oxygen species, and response to endoplasmic reticulum stress. The enrichment of these pathways indicates the activation of intrinsic neuronal mechanisms responsible for mounting an intracellular response to stress and the disruption of neuronal homeostasis. Additionally, pathways associated with programmed cell death were identified, including regulation of the apoptotic signalling pathway and extrinsic apoptotic signalling pathway. Furthermore, categories related to regeneration and repair were significantly enriched, such as wound healing, regeneration, and regulation of neurogenesis. The presence of these processes suggests that, under glutamate-induced toxicity, cells activate programs aimed at recovery and survival. Concurrent with the identification of wound healing processes, pathways governing cellular adhesion and reorganisation of the extracellular space in the neuroglial culture were also identified. These included regulation of cell–cell adhesion, extracellular structure organisation, and regulation of cell–substrate adhesion (Figure 11A,B). Analysis of the IC_vs._GL comparison group using the KEGG database confirmed activation of key signalling pathways, revealing a coordinated cellular response to glutamate-induced excitotoxicity. This response encompasses pathways mediating programmed cell death (apoptosis), pro-survival signalling (MAPK, PI3K-Akt, NF-kappa B, and JAK-STAT pathways), and integrated stress responses (TNF, HIF-1, FoxO, and p53 pathways). Additionally, pathways regulating cellular architecture and adhesion, including regulation of actin cytoskeleton, focal adhesion, and ErbB signalling, were significantly enriched (Figure 10C). Complementing these findings, gene set enrichment analysis (GSEA) was performed to identify functionally coordinated gene expression changes. Hallmark gene set analysis defined signatures significantly activated in the IC_vs._GL (Supplementary Materials Figure S9A) and GL_Mem_vs._GL (Supplementary Materials Figure S9B) using a significance threshold of *p*-value < 0.05 and normalised enrichment score (NES) directionality. 

### 3.9. The Effect of EV-GPCs on the Gene Expression Profile of Neuroglial Cultures Under Glutamate Exitotoxicity

Differential gene expression analysis identified 190 significantly up-regulated and 309 significantly down-regulated genes in the GL_EV_vs._GL comparison group (*p*-value < 0.05 and |FC| > 1.5). Complete DEG lists and corresponding heatmaps (Supplementary Materials Figure S10) are provided in Supplementary Materials. Supplementary Materials Figure S11 presents a volcano plot visualising the genome-wide expression differences between groups, highlighting the distinct molecular changes induced by extracellular vesicle supplementation following glutamate exposure. 

Analysis of up-regulated differentially expressed genes (DEGs) using the Gene Ontology database revealed significant enrichment in specific biological process categories. The most significant DEGs associated with these selected biological pathways are presented in Supplementary Materials Table S3. The predominant enriched categories included processes related to regeneration, extracellular matrix and cytoskeletal reorganisation, homeostasis maintenance, and cellular stress response. Regeneration-associated pathways encompassed wound healing, phosphatidylinositol 3-kinase/protein kinase B signal transduction, regeneration, tissue regeneration, regulation of wound healing, and regulation of axonogenesis. Extracellular matrix and cytoskeleton-related categories featured subprocesses, including cell–substrate adhesion, extracellular matrix organisation, actin filament organisation, and positive regulation of cell–substrate adhesion.

Furthermore, the stress response category demonstrated significant enrichment for processes, including gliogenesis, response to reactive oxygen species, cellular response to chemical stress, cellular response to oxidative stress, reactive oxygen species metabolic process, response to hydrogen peroxide, cellular response to reactive oxygen species, and response to axon injury.

**Figure 12:**
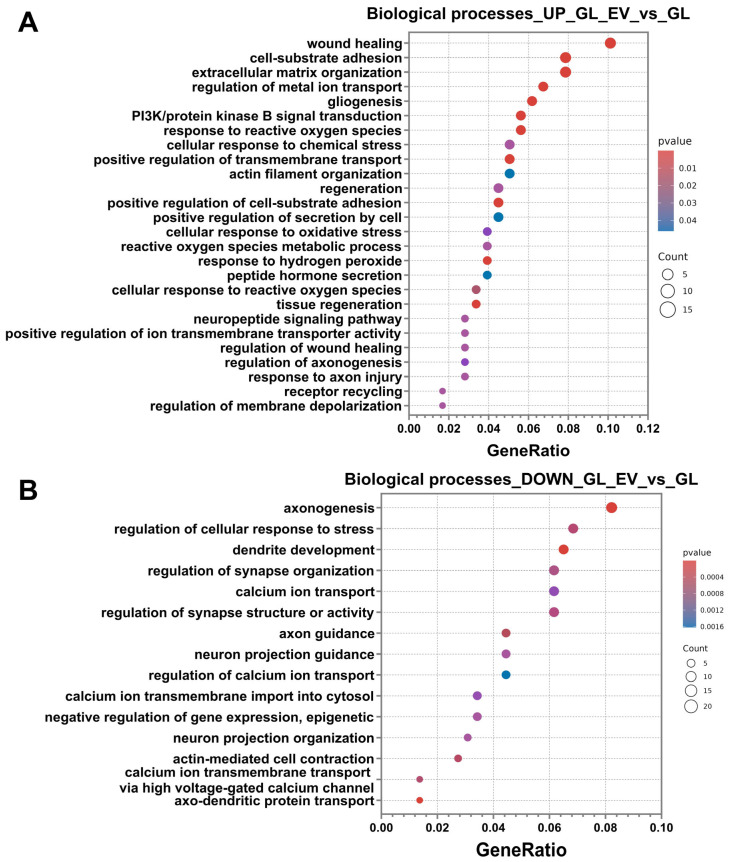
RNA-sequencing results of the glutamate-added group (GL) and the EV-GPC-added group (GL_EV). (**A**) the results of up-regulated DEG classification using the Gene Ontology Biological Processes database. (**B**) the results of down-regulated DEG classification using the Gene Ontology Biological Processes database. The X-axis shows the ratio of pathway DEGs to the total number of DEGs, and the Y-axis shows the selected biological processes.

The final category of identified processes involved the maintenance of intracellular homeostasis, which encompassed the following pathways: regulation of metal ion transport, positive regulation of transmembrane transport, positive regulation of secretion by cell, peptide hormone secretion, neuropeptide signalling pathway, positive regulation of ion transmembrane transporter activity, receptor recycling, and regulation of membrane depolarisation (Figure 12A).

Analysis of down-regulated pathways revealed significant enrichment of processes associated with neuronal physiology, including axonogenesis, regulation of cellular response to stress, dendrite development, regulation of synapse organisation, calcium ion transport, regulation of synapse structure or activity, axon guidance, neuron projection guidance, regulation of calcium ion transport, calcium ion transmembrane import into cytosol, negative regulation of epigenetic gene expression, neuron projection organisation, actin-mediated cell contraction, regulation of calcium ion transmembrane transport via high voltage-gated calcium channel, and axo-dendritic protein transport (Figure 12B).

**Figure 13:**
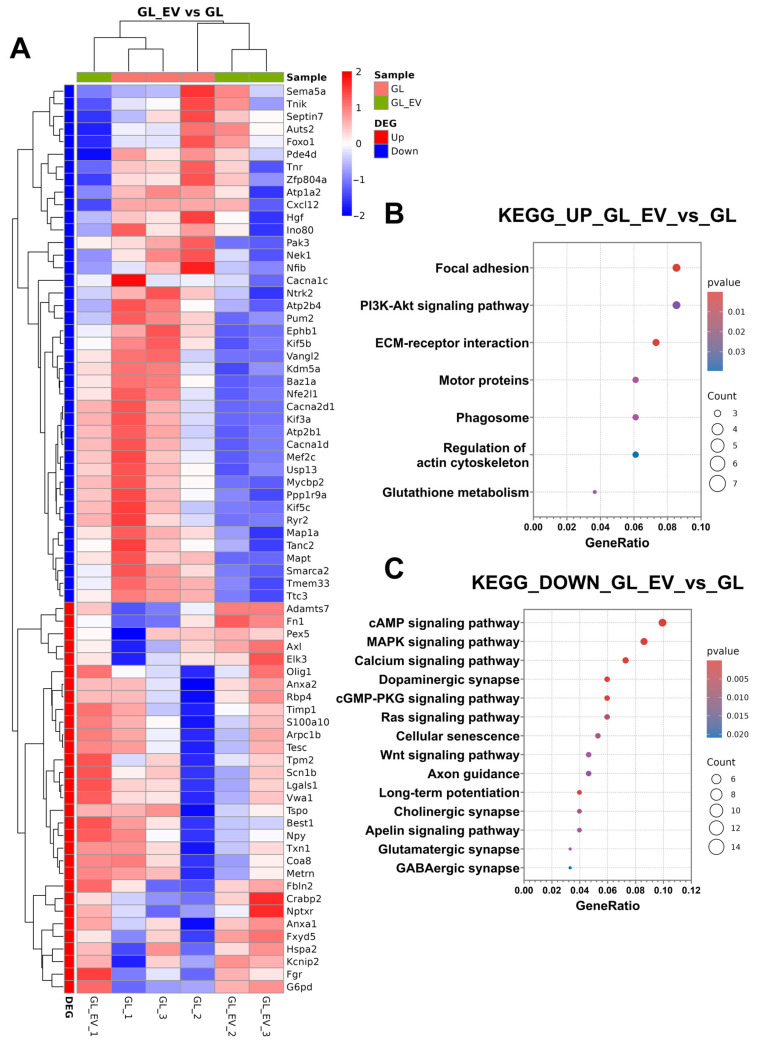
Transcriptomic profiling of neuroglial cultures following EV-GPC treatment in modelling glutamate excitotoxicity. (**A**) heatmaps of differentially expressed genes (DEGs) were obtained using pheatmap R package. Each row represents a gene and each column corresponds to an individual sample; sample groups indicated by column annotations. Log2-transformed gene expression values were row-scaled, and hierarchical clustering was performed for both rows (genes) and columns (samples) using Euclidean distance and the complete clustering method. The colour scale represents scaled expression values and ranges from blue (down-regulated) to red (up-regulated).; (**B**) results of classification of up-regulated DEGs using the KEGG database. The ratio of DEGs of the pathway to the total number of DEGs is shown on the X axis, and the selected signalling pathways are shown on the Y axis. (**C**) results of classification of down-regulated DEGs using the KEGG database. The ratio of DEGs of the pathway to the total number of DEGs is shown on the X axis, and the selected signalling pathways are shown on the Y axis.

For deeper functional insights, we performed KEGG pathway enrichment analysis, consistent with our previous experimental approach. Additionally, Figure 13A displays a heatmap of selected differentially expressed genes, illustrating specific expression patterns across experimental conditions. Up-regulated DEGs showed significant enrichment in several signalling pathways, including focal adhesion, PI3K-Akt signalling pathway, ECM–receptor interaction, motor proteins, phagosome, regulation of actin cytoskeleton, and glutathione metabolism (Figure 13A). Down-regulated DEGs were significantly associated with pathways, including cAMP signalling pathway, MAPK signalling pathway, calcium signalling pathway, dopaminergic synapse, cGMP-PKG signalling pathway, Ras signalling pathway, cellular senescence, Wnt signalling pathway, axon guidance, long-term potentiation, cholinergic synapse, Apelin signalling pathway, glutamatergic synapse, and GABAergic synapse (Figure 13B).

Gene set enrichment analysis (GSEA) identified coordinated gene expression patterns distinguishing the glutamate (GL) and extracellular vesicle-treated (GL_EV) groups. Supplementary Materials Figure S12 presents Hallmark gene set results, demonstrating signatures significantly activated in the extracellular vesicle-treated group (GL_EV) (NES > 0) compared with the glutamate group (GL) (NES < 0) at *p*-value < 0.05.

## 4. Discussion

Dysregulation of the glutamatergic system is a fundamental pathogenetic mechanism underlying neural tissue damage in a variety of neurological disorders. Disruption of the glutamate balance in the nervous system leads to the development of excitotoxicity and irreversible damage to neurons. Current approaches to the treatment of glutamate excitotoxicity have limited efficacy because of the complexity of the pathogenetic mechanisms of this process. To date, therapy based on extracellular vesicles (EVs) is a promising tool for regenerative medicine as it has several advantages over cell therapeutic strategies and conventional pharmacological drugs [73]. EVs are spherical membrane structures formed by invagination of the cytoplasmic membrane during exocytotic processes. The structural organisation of EVs, including a bilipid layer, ensures their long-term stability in tissue fluid and systemic blood flow. The presence of receptor proteins on the vesicle surface ensures their specific interaction with target cells, and the multicomponent composition helps to achieve a complex effect on cellular structures [74]. This work investigated the neuroprotective effect of EVs derived from glial progenitor cells in a model of glutamate excitotoxicity. EV-GPCs neutralised the effect of glutamate excitotoxicity and increased the survival of cortical neuroglial cells. This effect is probably due to the presence of proteins in EV-GPCs that inhibit the apoptotic cascade, such as the 14-3-3 protein, which is an inhibitor of the proapoptotic protein Bad, which may indicate the ability of EVs to reduce cell death. Proteins related to the development of the nervous system and regeneration have also been found to have a protective effect against toxic effects, such as the proteins PEDF and Copine 1 [75].

As a predominant in vitro model of nervous tissue, primary neuroglial cultures derived from the cortex recapitulate the interplay between neurons and glial cells. This complexity permits the assessment of integrated tissue responses to toxic stimuli, such as glutamate-induced excitotoxicity [76]. It is well-established that astrocytes are markedly less susceptible to high-dose glutamate exposure compared with neurons. Consequently, a 100 μM glutamate challenge typically results in extensive neuronal death. The toxic effect is mediated primarily by the interaction of glutamate with its cognate receptors on neuronal surfaces and, to a lesser extent, on astrocytes. During synaptic transmission, glutamate is released into the synaptic cleft and binds to specific receptors on the postsynaptic membrane. The activation of these receptors leads to an increased influx of calcium and sodium ions into the cytosol, depolarisation of the membrane, and electrical signal transmission. These processes stimulate the activation of intracellular signalling cascades that cause changes in intracellular homeostasis. Pathological accumulation of glutamate causes hyperstimulation of receptors (the phenomenon of excitotoxicity), which leads to a critical increase in intracellular Ca^2+^ and eventually to neuronal death [77]. Calcium imaging showed that pre-incubation of neuroglial cells with EV-GPCs leads to a less efficient accumulation of free calcium in the cytoplasm. Such an effect could presumably be related to a more efficient operation of the calcium exchangers that remove excess calcium from the cells into the intercellular space, as well as to a more efficient operation of the ATP-dependent pumps at the membrane of the endoplasmic reticulum and the outer membrane of the mitochondria, as both organelles can serve as calcium depots [78,79]. Next, the ability of cortical cells to restore their original level of intracellular calcium after cytoplasmic overload was analysed. It was found that neuroglial cells pre-incubated with EV-GPCs restored the original level of cytosolic calcium more effectively than the control culture, which could also be related to changes in the functioning of the calcium exchangers. According to transcriptome analysis, the addition of EV-GPCs after exposure to glutamate decreased the expression of genes related to the development of the glutamatergic synapse, such as *Plcb1*, *Prkacb*, *Cacna1c*, and *Cacna1d* [80], as well as with calcium transport in cells, reducing the expression of calcium transmembrane channel genes such as *Atp2b4*, *Atp2b1*, *Plcb1*, and *Ryr2*, which contributes to a less efficient entry of calcium ions into the cytosol of cells upon calcium overload. In addition, the incubation of cortical neuroglial cells with EV-GPCs reduced the expression of genes responsible for dendrite growth and branching, which may also contribute to the protective effect, as a reduction in the number of dendritic spines reduces the total number of synapses and thus reduces the total number of receptors that are channels for calcium entry. This is consistent with existing data showing that neuroglial cells can suppress the formation of processes and synapses under certain conditions, particularly during processes that are essential for survival. This suppression is not a general suppression of dendritic and axonal development, but a specific mechanism that priorities survival under conditions of limited resources or stress [81,82]. One of the most important consequences of cytosolic overload is the secondary accumulation of Ca^2+^ in the mitochondria, which acts as a trigger for the impairment of the electron transport chain and promotes oxidative stress, the synthesis of reactive oxygen species (ROS), and the disruption of cellular respiration. Furthermore, the increased permeability of the mitochondrial membrane contributes to the activation of cell death pathways [83]. These findings are consistent with the data obtained in the present study, as rhodamine-based imaging showed that a 15 min exposure to glutamate caused a collapse of the mitochondrial membrane potential (ΔΨm) in cortical cells. At the same time, the addition of EV-GPCs stabilised mitochondrial function during glutamate toxicity, which contributed to the neuroprotection of neuroglial cells and more effectively restored the mitochondrial potential to baseline after glutamate exposure. Proteomic analysis revealed that EV-GPCs contain proteins associated with redox processes, the response to hypoxia, and the response to reactive oxygen species. These include various peroxiredoxins involved in the Hif-1-alpha signalling pathway and the protein Hypoxia Up-Regulated 1, which responds to reduced oxygen levels in cells. These data were confirmed by transcriptomic analysis, as the addition of extracellular vesicles increased the expression of genes related to the above signalling pathways. Moreover, several proteins central to the glutathione system were detected, such as glutathione synthetase and subunits of glutathione peroxidase and reductase, which collectively facilitate glutathione synthesis and its antioxidant function in neutralising reactive oxygen species. Consistent with these findings, transcriptome analysis confirmed that vesicles stimulate the up-regulation of genes related to this antioxidant’s metabolic pathway. It is well known that reactive oxygen species, including hydrogen peroxide, are the main cause of membrane permeability disruption, leading to the breakdown of mitochondrial membrane potential [84]. Their neutralisation therefore helps to the maintenance of proper mitochondrial function. These data suggest that EVs exert their neuroprotective effect by stabilising mitochondrial function during glutamate hyperstimulation through the attenuation of oxidative stress and the removal of reactive oxygen species.

Memantine was selected as the positive control in this experiment. Memantine is an FDA-approved drug for the treatment of various neurological disorders. Its simultaneous use with glutamate increased the survival rate of neuroglial cells to the control level. The mechanism of action of memantine is based on its interaction with NMDA receptors and inhibition of calcium ion influx into the cells, which promotes neuron survival and protects them from calcium overload [85]. Comparison of EV-GPCs and memantine with the MTT assay showed that both agents increased neuronal survival to control levels during glutamate exposure. Morphometric analysis of cell death showed that both compounds also significantly increased the proportion of viable cells and significantly decreased the proportion of necrotic cells.

The mechanism of action of EVs is based on the primary interaction with the target cells and begins with attachment to the cell membrane by special receptor proteins that recognise the corresponding molecules on the vesicle surface [19]. After attachment, EVs are internalised by phagocytosis, which is characterised by a latency period of several hours, or by membrane fusion with a time interval of 1–3 h, depending on the initial concentration of extracellular vesicles in the medium, and complete accumulation after 10–12 h [86]. After internalisation, the vesicle content is released into the cytoplasm or intercellular space and signalling pathways are initiated. The content of extracellular vesicles assessed by proteomic analysis revealed proteins that can initiate signalling pathways NIK-Nf-kappaB, PI3K-Akt, MAPK, HIF-1, and Apelin, which was confirmed by transcriptomic analyses during the incubation of EV-GPCs with intact cortical neuroglial cells.

Upon glutamate excitotoxicity, the addition of EV-GPCs triggered the activation of the following signalling pathways: PI3K-Akt, focal adhesion, phagosome, and regulation of the actin cytoskeleton regulation. At the same time, the signalling pathways responsible for the formation of dopaminergic, glutamatergic, cholinergic, and GABAergic synapses, long-term potentiation, axonal growth direction, and calcium signalling were inhibited. Despite the similar efficacy of the neuroprotective effects of EV-GPCs and memantine, the signalling pathways they activated were different. The addition of memantine led to increased expression of genes associated with the following signalling pathways: axonogenesis, dendritic tree expansion, control of neurotransmitter synthesis, regulation of membrane depolarisation, and glutamatergic synapse formation, as well as MAPK signalling, neurotrophin signalling, and synaptic vesicle synthesis cycle. At the same time, memantine did not activate signalling pathways related to PI3K-Akt, regeneration, or oxidative stress response, but on the contrary stimulated the development of different types of synapses and supported neurotransmitter transmission. These processes indicate that the two drugs follow different pathways of neuroprotection. While memantine inhibits the influx of calcium ions into the cell under the influence of excess glutamate by binding to calcium transporters, EV-GPCs stimulate the pathways within cells responsible for the protective effect of glutamate by decreasing the expression of genes related to neurotransmission and increasing the expression of survival signalling pathway genes, such as PI3K-Akt. Thus, the therapeutic action of memantine is rooted in its non-competitive antagonism of NMDA receptors, which reduces pathological glutamate excitotoxicity. While effective, this approach of receptor blockade is associated with clinical side effects and may impose a physiological trade-off by impeding the essential roles of glutamate in neurotransmission. This can perturb the excitation/inhibition balance and, at high concentrations, has been linked to axonal degeneration [87,88]. EV-GPCs suggest an alternative strategy: rather than blocking synaptic receptors, the extracellular vesicle preparation we investigated activates endogenous protective signalling, including the PI3K-Akt pathway. This intracellular activation is hypothesised to stabilise neurons against glutamate toxicity while preserving synaptic homeostasis, thereby promoting neuronal longevity through a fundamentally different mechanism. Consequently, we posit that the principal advantage of the extracellular vesicle preparation over memantine lies in its ability to concurrently activate several protective pathways while preserving physiological glutamate signalling, thereby avoiding the disruption of excitation/inhibition balance.

One of the main pathways involved in the neuroprotection of cortical neurons and astrocytes is PI3K-Akt. This is one of the most extensive signalling pathways responsible for the maintenance of homeostasis, control of intracellular calcium levels, survival, growth, and development of the dendrite tree, and negative regulation of apoptosis [89,90]. There are also hypotheses that this pathway may be triggered by the binding of glutamate to its receptors on the postsynaptic membrane. Ionotropic glutamate receptors, specifically NMDA receptors, are heterotetramers typically composed of obligatory GluN1 and modulatory GluN2 (A-D) or GluN3 (A-B) subunits. Their function is critically determined by this subunit composition [91]. According to the prevailing subunit composition hypothesis, receptors containing GluN2A subunits predominantly activate neuroprotective cascades, such as those involving CREB and the PI3K-Akt pathway [92]. In contrast, those containing GluN2B subunits are linked to excitotoxicity and pro-death signalling, including PTEN-mediated dephosphorylation of Akt and Bad, a finding corroborated by C-terminal domain swap studies in mouse models [93,94]. If this hypothesis holds, the glutamate challenge in our study would be expected to simultaneously engage both pro-death (via GluN2B) and pro-survival (via GluN2A, including PI3K-Akt) pathways, a duality that aligns with our observed results. However, although activation of PI3K-Akt is a stimulus for neuron survival; nevertheless, under the severe stress of excitotoxicity, this defence is likely insufficient to suppress the dominant apoptotic program, resulting in the observed cell death.

Proteomic analysis revealed proteins that are activators of this signalling pathway: HSP90 protein subunits, Jak kinase subunits, and Cdc37 protein. Its activation during glutamate excitotoxicity may play a key role in the mechanisms of neuroprotection. In the glutamate excitotoxicity model, the addition of a selective PI3Kᵧ inhibitor—AS605240—completely neutralised the neuroprotective effect of EV-GPCs. Transcriptomic analyses also confirmed that EV-GPCs increase the expression of genes that activate this signalling cascade in both intact cells and cells exposed to glutamate. Previously, the involvement of PI3K-Akt in the neuroprotective effect of extracellular vesicles isolated from MSCs and NSCs has been demonstrated in models of glucose deprivation, oxidative stress, and amyloid and glutamate toxicity, which is consistent with the data obtained in this study [95,96,97,98,99,100,101,102,103]. Based on converging evidence from proteomic, inhibitory, and transcriptome analyses, we propose that extracellular vesicles enhance neuronal survival by activating the PI3K-Akt pathway, an effect likely driven by specific proteins in the EV cargo. The neuroprotective role of EV-derived proteins, however, may be part of a broader mechanism. Given the diverse cargo of EVs—including proteins, peptides, and microRNAs—it is plausible that other signalling pathways are co-activated, as suggested by our proteomic data and prior work on the microRNA-dependent effects of EV-GPCs [21].

Thus, this study demonstrates that extracellular vesicles from human glial progenitor cells have a neuroprotective effect related to the regulation of intracellular homeostasis, calcium ion transport, and maintenance of mitochondrial function. Presumably, the mechanism of the neuroprotective effect is linked to the activation of the PI3K-Akt signalling pathway. However, further studies are needed to investigate the mechanisms of the neuroprotective effect of extracellular vesicles in more detail.

## 5. Conclusions

The results suggest that extracellular vesicles secreted by human GPCs may exert a neuroprotective effect in glutamate excitotoxicity. Mechanisms likely include a reduction in free cytoplasmic calcium levels and stabilisation of mitochondrial potential against a background of glutamate action due to the activation of various signalling pathways, including PI3K-Akt. The observed multitarget effect appears to be associated with EV-GPC multicomponent cargo and may be mediated by proteins involved in negative regulation of apoptotic processes, regeneration activation, calcium ion transport modulation, and regulation of membrane depolarisation.

## Data Availability

The data generated or analysed during the current study that are relevant to the results presented are included in this article and its Supplementary Materials files. Other data that were not relevant to the results presented here are available from the corresponding authors on reasonable request. “The mass spectrometry proteomics data have been deposited to the ProteomeXchange Consortium via the PRIDE [http://www.ebi.ac.uk/pride (accessed on 19 August 2025)] partner repository with the dataset identifier PXD067360 and 10.6019/PXD067360”. Transcriptomic data have been deposited to the Gene Expression Omnibus, Series numbers for publication GSE282138 and GSE307721.

## Supplementary Materials

The following supporting information can be downloaded at: https://www.mdpi.com/article/10.3390/cells14231915/s1, Figure S1: Results of clustering using principal component analysis of DeSeq number of genes from IC (n = 3) and EV (n = 3); Figure S2: Transcriptomic profiling of IC_vs._EV comparison group; Figure S3: Volcano plot showing the level of gene expression depending on the fold change and *p*-value (*p* < 0.05 and |FC| < 1.5); Figure S4: Gene set enrichment analysis (GO-base) in the comparison group IC_vs._EV; Figure S5: Results of clustering of DeSeq number of genes from IC (n = 3), GL, GL_Mem (n = 3) and GL_EV (n = 3); Figure S6: Transcriptomic profiling of IC_vs._GL comparison group; Figure S7: Transcriptomic profiling of GL_Mem_vs._GL comparison group; Figure S8: Volcano plot showing the level of gene expression depending on the fold change and *p*-value (*p* < 0.05 and |FC| < 1.5); Figure S9:: Gene set enrichment analysis (GO-base) in the comparison group IC_vs._GL (A) and GL_Mem_vs._GL (B); Figure S10: Transcriptomic profiling of GL_EV_vs._GL comparison group; Figure S11: Volcano plot showing the level of gene expression depending on the fold change and *p*-value (*p* < 0.05 and |FC| < 1.5); Figure S12: Gene set enrichment analysis (GO-base) in the comparison group GL_EV_vs._GL; Table S1: Lists of the most significant down-regulated differentially expressed genes (DEGs) associated with enriched biological pathways in the IC_vs._EV comparison group; Table S2: Lists of the most significant differentially expressed genes (DEGs) associated with enriched biological pathways in the IC_vs._GL and GL_Mem_vs._GL comparison groups; Table S3: Lists of the most significant differentially expressed genes (DEGs) associated with enriched biological pathways in the GL_EV_vs._GL comparison group. References [104,105,106,107,108,109,110,111,112,113,114,115,116,117,118,119,120,121,122,123,124,125,126,127,128,129,130,131,132,133,134,135,136,137,138,139,140,141,142,143,144,145,146,147,148,149,150,151,152,153,154,155,156,157,158,159,160,161,162,163,164,165,166,167,168,169,170,171,172,173,174,175,176,177,178,179,180,181,182,183,184,185,186,187,188,189,190,191,192,193,194,195,196,197,198,199,200,201,202,203,204,205,206,207,208,209,210,211,212,213,214,215,216,217,218,219,220,221,222,223,224,225,226,227,228,229,230,231,232,233,234,235,236,237,238,239,240,241,242,243,244,245,246,247,248,249,250,251,252,253,254,255,256,257,258,259,260,261,262,263,264,265,266,267,268,269,270,271,272,273,274,275,276,277,278,279,280,281,282,283,284,285,286,287,288,289,290,291,292,293,294,295,296,297,298,299,300,301,302,303] are cited in the supplementary materials.
