# Supplementary material for: Extracellular Vesicles from iPSC-Derived Glial Progenitor Cells Prevent Glutamate-Induced Excitotoxicity by Stabilising Calcium Oscillations and Mitochondrial Depolarisation
Source: Cells. 2025 Dec 2;14(23):1915. doi: 10.3390/cells14231915 (PMC12691032; doi:10.3390/cells14231915)
Supplement: Supplementary file 1 [file cells-14-01915-s001.zip › Supplementary Materials.pdf]

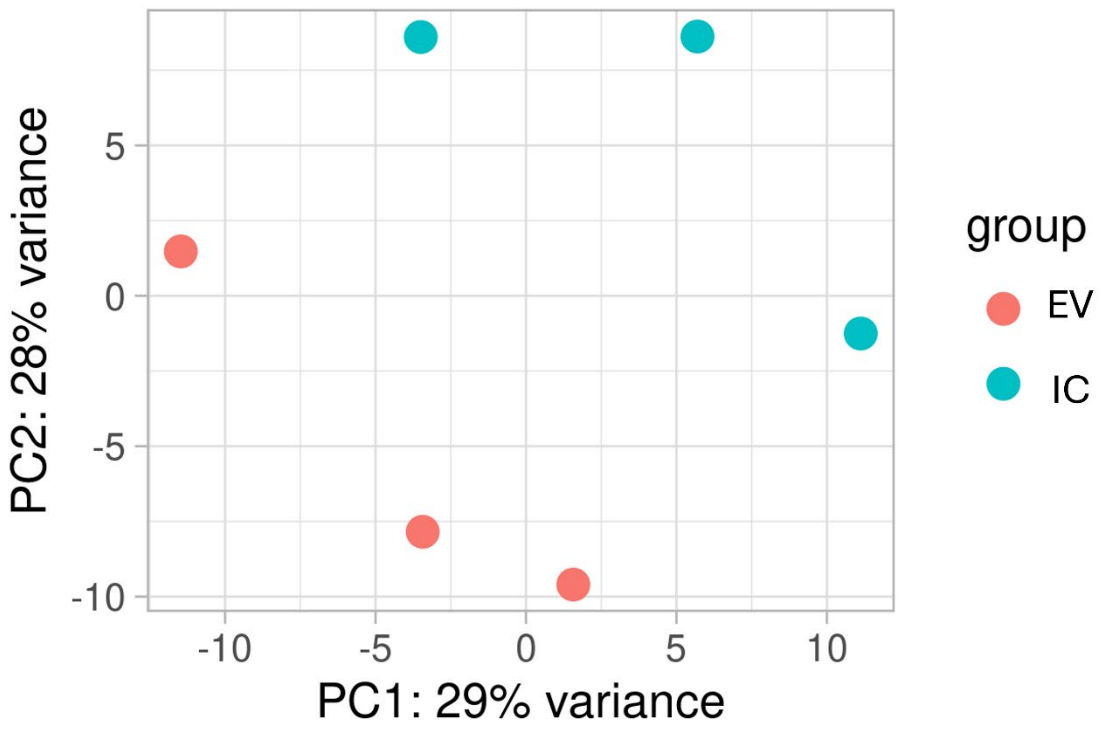

Figure S1. Results of clustering using principal component analysis of DeSeq number of genes from IC (n=3) and EV (n=3). The numbers on the axes represent the percentage of total variance explained by the first and second PC. Principal Component Analysis (PCA) was performed on the variance-stabilised and batch-corrected counts using the DESeq2 package. The removeBatchEffect limma function was applied to remove variation attributable to the 'day' covariate. The resulting data was projected onto its first two principal components using the top 200 most variable genes, and visualised while annotating for 'condition'.

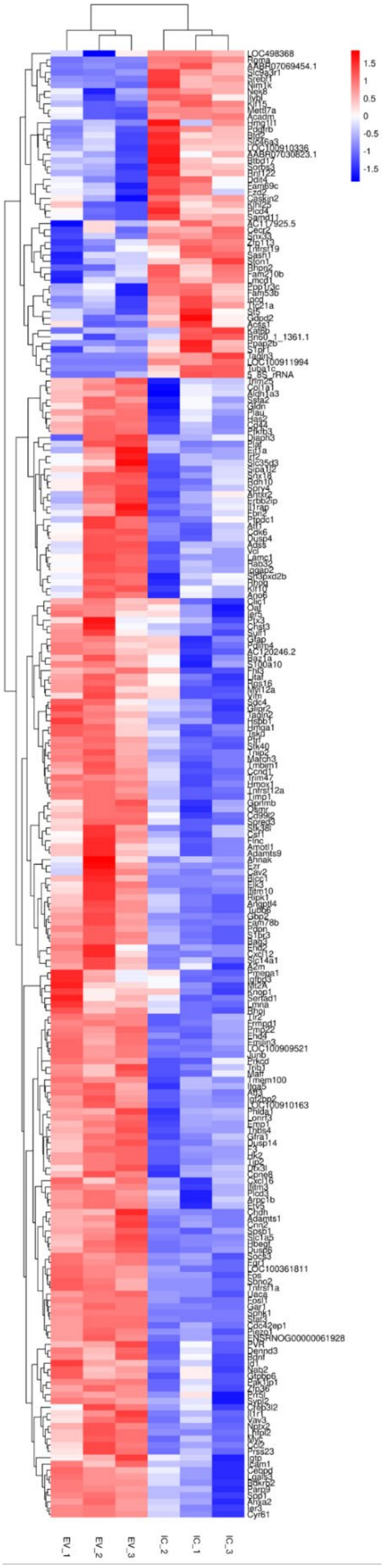

1.5
1
0.5
0
-0.5
-1
-1.5
EV_1
EV_2
EV_3
IC_2
IC_1
IC_3

Figure S2. Transcriptomic profiling of IC_vs_EV comparison group. Heatmaps of differentially expressed genes (DEGs) were obtained using pheatmap R package. Each row represents a gene and each column corresponds to an individual sample, sample groups indicated by column annotations. Gene expression values were row-scaled, hierarchical clustering was performed for both rows (genes) and columns (samples) using Euclidean distance and the complete clustering method. The color scale represents scaled expression values and ranges from blue (down-regulated) to red (up-regulated).

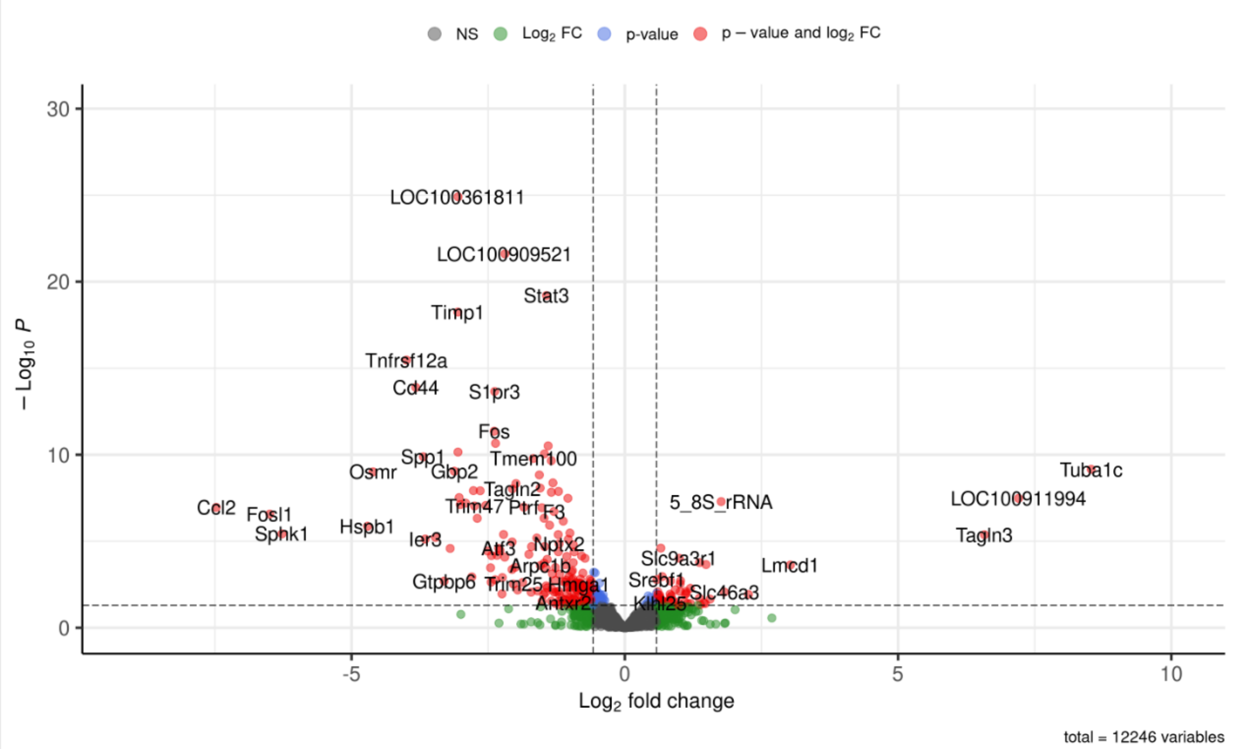

Figure S3. Volcano plot showing the level of gene expression depending on the fold change and p-value (p<0.05 and |FC|<1.5), the expression of genes in the control group is shown relative to the ex-perimental group, FC<1.5 – down-regulated genes, FC>1.5 – up-regulated genes in the comparison group IC_vs_EV.

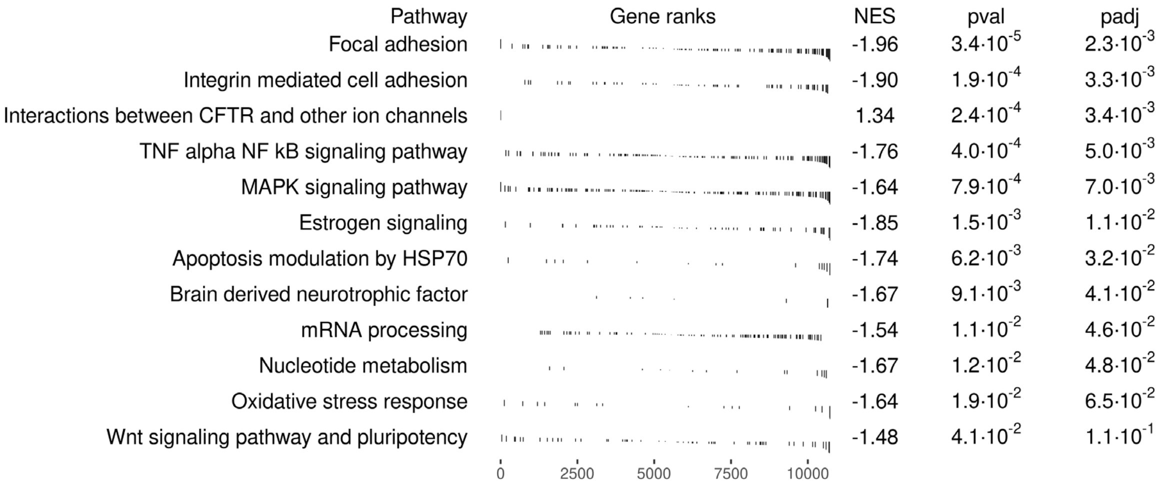

Figure S4. Gene set enrichment analysis (GO-base) in the comparison group IC_vs_EV.

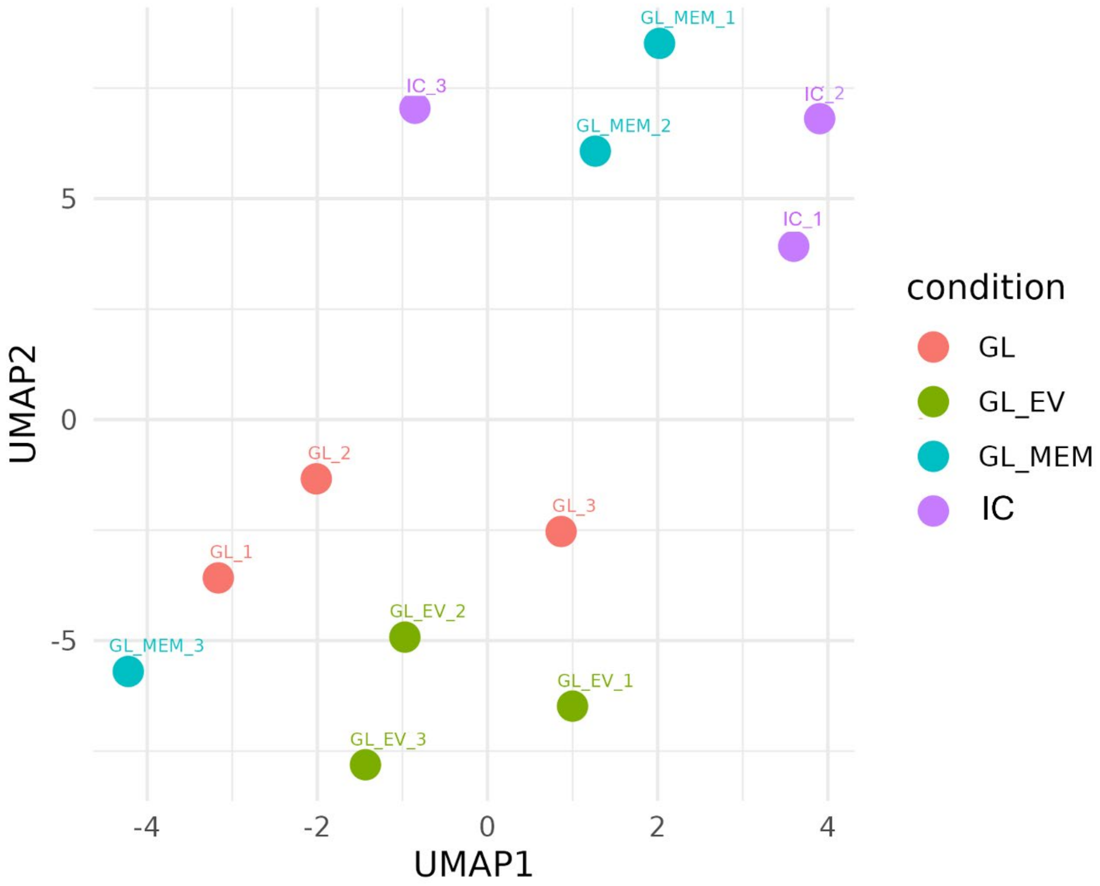

Figure S5. Results of clustering of DeSeq number of genes from IC (n=3), GL, GL_Mem (n=3) and GL_EV (n=3). Two-dimensional UMAP was obtained with umap R package using the top 200 most variable genes identified from the variance-stabilized and batch-corrected counts, with UMAP parameters specifically configured to a neighborhood size of 6, a minimum distance of 1, a spread of 3, and a cosine distance metric.

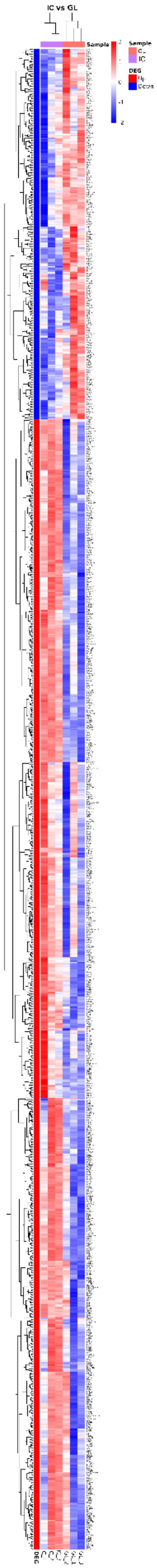

IC vs GL
Sample
Sample
IC
DEG
Down
0
-1
2
DEG

Figure S6. Transcriptomic profiling of IC_vs_GL comparison group. Heatmaps of differentially expressed genes (DEGs) were obtained using pheatmap R package. Each row represents a gene and each column corresponds to an individual sample, sample groups indicated by column annotations. Gene expression values were row-scaled, hierarchical clustering was performed for both rows (genes) and columns (samples) using Euclidean distance and the complete clustering method. The color scale represents scaled expression values and ranges from blue (down-regulated) to red (up-regulated).

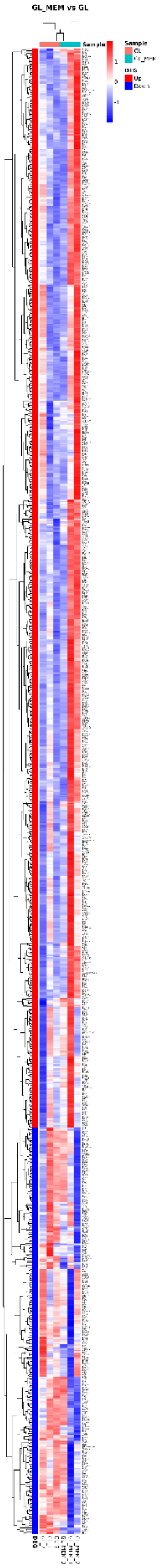
GL_MEM vs GL
Sample
GL
1
0
-1
DEG
Up
DEG

Figure S7. Transcriptomic profiling of GL_Mem_vs_GL comparison group. Heatmaps of differentially expressed genes (DEGs) were obtained using pheatmap R package. Each row represents a gene and each column corresponds to an individual sample, sample groups indicated by column annotations. Gene expression values were row-scaled, hierarchical clustering was performed for both rows (genes) and columns (samples) using Euclidean distance and the complete clustering method. The color scale represents scaled expression values and ranges from blue (down-regulated) to red (up-regulated).

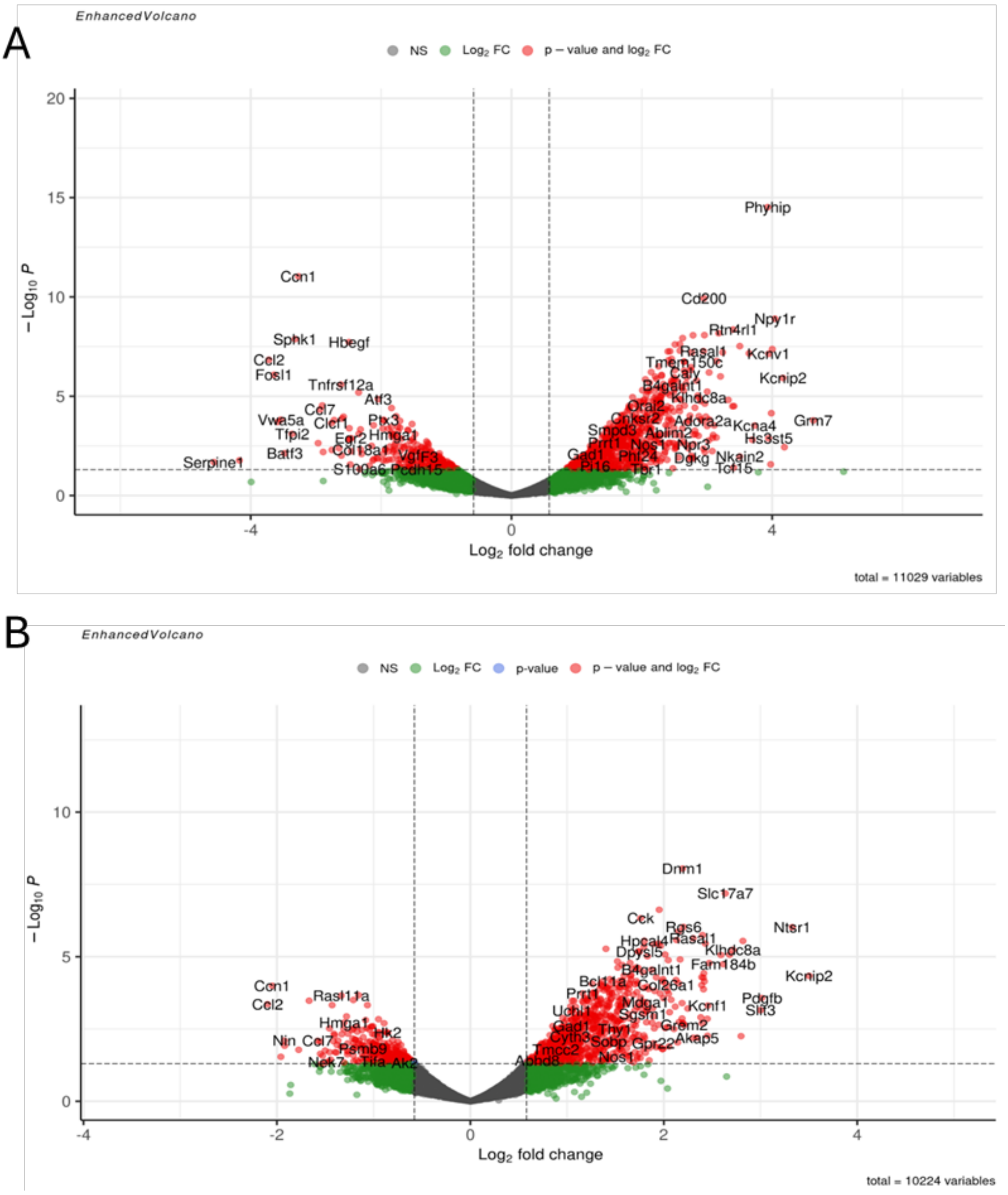

Figure S8. Volcano plot showing the level of gene expression depending on the fold change and p-value ($p<0.05$ and $|FC|<1.5$), A, B – Volcano plots showing the gene expression level depending on the fold change and p-value (p-value <0.05 and $|FC|<1.5$) in the comparison groups IC_vs_GL (A), GL_Mem_vs_GL (B).

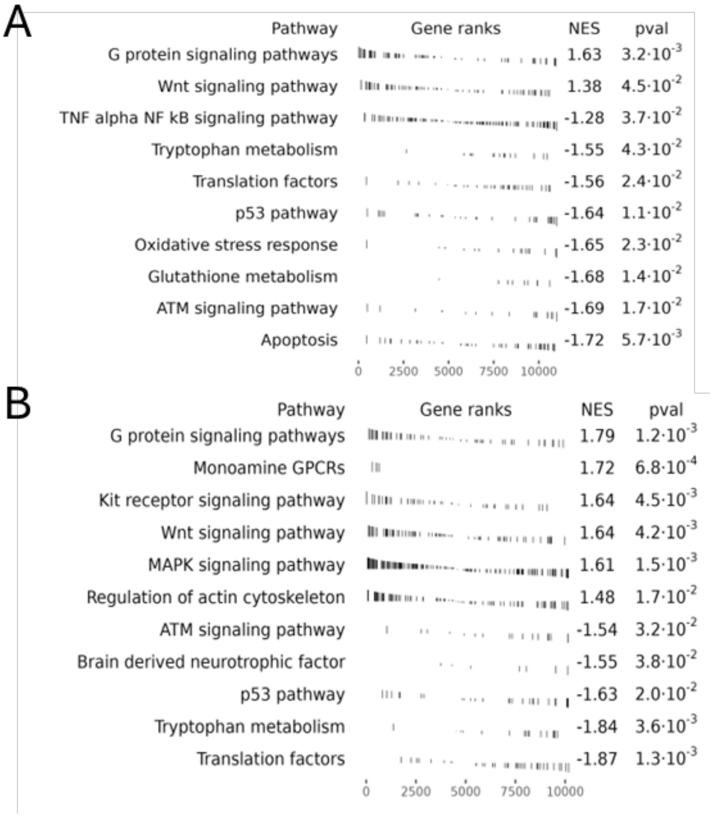

Figure S9. Gene set enrichment analysis (GO-base) in the comparison group IC_vs_GL (A) and GL_Mem_vs_GL (B).

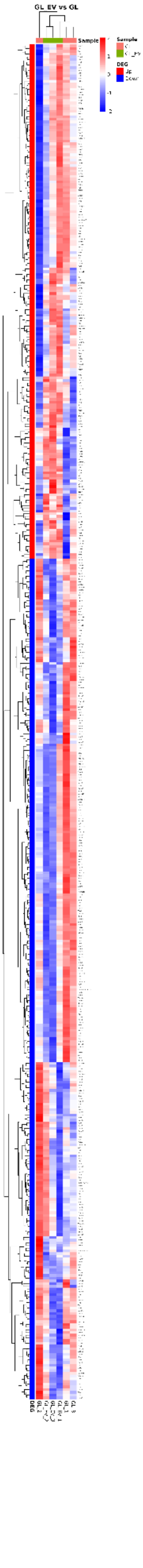
GL_EV vs GL
Sample
1
0
-1
2
Sample
DEG

Figure S10. Transcriptomic profiling of GL_EV_vs_GL comparison group. Heatmaps of differentially expressed genes (DEGs) were obtained using pheatmap R package. Each row represents a gene and each column corresponds to an individual sample, sample groups indicated by column annotations. Gene expression values were row-scaled, hierarchical clustering was performed for both rows (genes) and columns (samples) using Euclidean distance and the complete clustering method. The color scale represents scaled expression values and ranges from blue (down-regulated) to red (up-regulated).

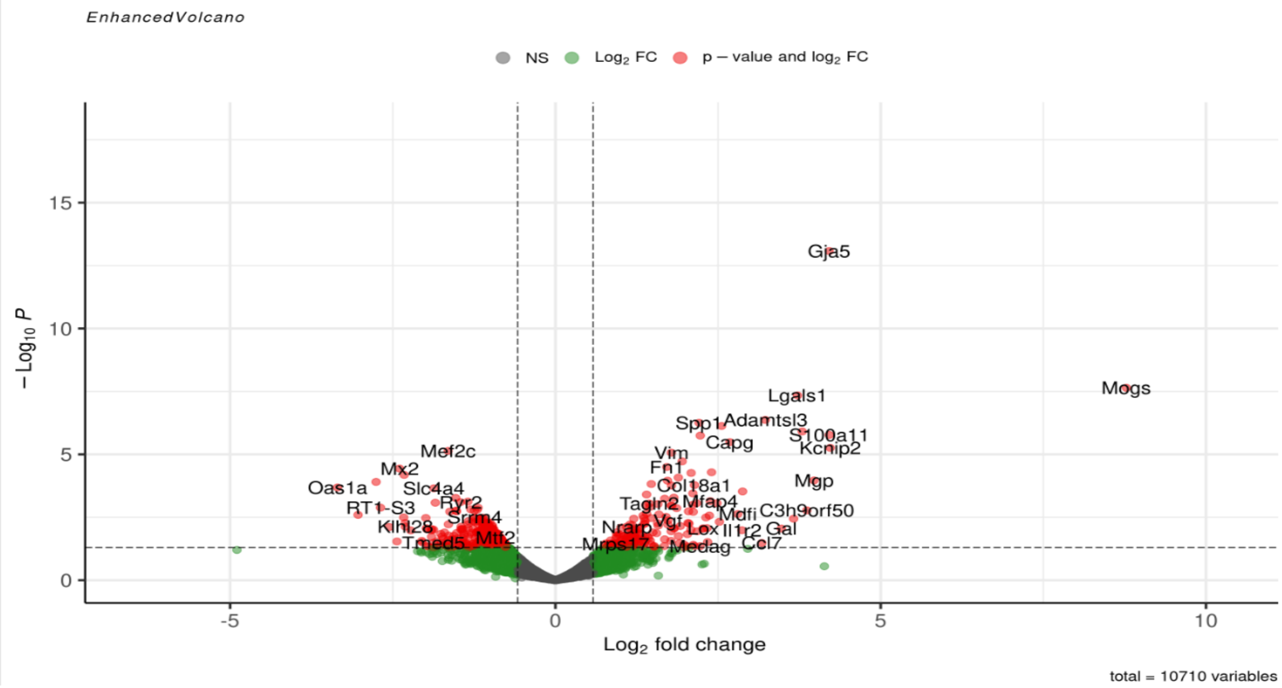

Figure S11. Volcano plot showing the level of gene expression depending on the fold change and p-value ($p<0.05$ and $|FC|<1.5$), the expression of genes in the control group is shown relative to the ex-perimental group, FC<1.5 – down-regulated genes, FC>1.5 – up-regulated genes in the comparison group GL_EV_vs_GL.

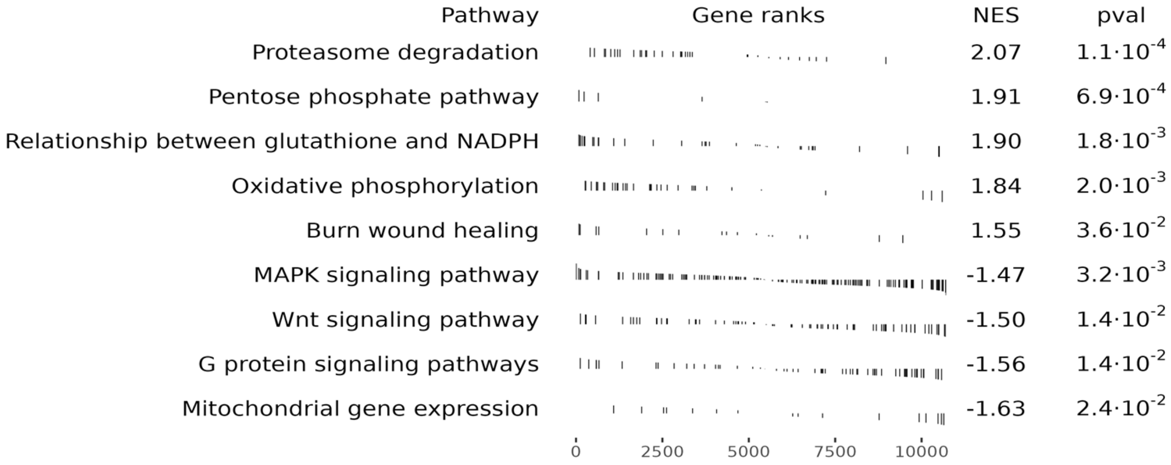

Figure S12. Gene set enrichment analysis (GO-base) in the comparison group GL_EV_vs_GL.

Up/Down-regulated	Name of the gene	Description	GO_Biological processes

**Up-regulated**	*Elk3*	transcription factor of cell cycle, cell growth and neural tissue development [1]	wound healing
	Tnfrsf12a	receptor that activates various pathways in neurons[2]	
	Timp1	inhibitor of metalloproteinases responsible for the survival and neuroprotection of neurons [3]	
	Anxa2	gene is critical for axonal growth and cytoskeletal stabilization in neurites [4]	
	Vcl	gene controls axon growth through interaction with the extracellular matrix [5]	
	F3	gene controls axon growth through interaction with the extracellular matrix [6]	
	Tsku	gene interacts with molecules involved in Wnt signaling and TGF-beta signaling [7]	
	Hk2	gene controls glucose metabolism and energy production [8]	response to hypoxia
	Hmox1	gene that controls redox balance and is involved in various cellular processes, including antioxidant defense and antiapoptosis [9]	
	Stat3	gene stimulates the synthesis of genes responsible for reducing ROS [10]	
	Il1rap	interleukin-1 receptor, which in neurons is involved in the development and maintenance of synapses [11]	cytokine-mediated signaling pathway
	Cd44	receptor that binds to hyaluronic acid and is responsible for axonal growth [12]	
	Ccl2	gene is necessary for synapse function and is also involved in neuropeptide transduction [13]	
	Cxcl12	chemokine that controls migration processes, the direction of axon growth, and interactions with other cells [14]	
	Tnfrsf1a	TNF receptor, the activation of which leads to the induction of many processes associated with inflammation, neuroprotection and cell homeostasis [15]	
	Egr1	gene responsible for signals in the synaptic cleft and synaptic plasticity [16]	
	Antxr2	gene remodels extracellular matrix [17]	extracellular matrix organization
	Adamts1	gene remodels extracellular matrix [18]	
	Pmp22	gene helps maintain the myelin sheath around the processes [19]	
	Has2	hyaluronic acid synthetase [20]	
	Sulf1	gene modifies heparan sulfate, thereby being able to modify various signaling pathways [21]	
	Sh3pxd2b	gene controls the actin cytoskeleton and cell adhesion [22]	cell-substrate adhesion

	Arpc1b	gene that controls actin polymerization and cell migration [23]	actin filament organization
	Tagln2	control the growth of dendritic spines and the elongation of axons [24]	
	Spred3	Ras/MAPK cascade inhibitors [25]	ERK1 and ERK2 cascade
	Dusp6	Ras/MAPK cascade inhibitors [26]	
	Glipr2	activators of the pathway that stimulates the development and survival of neurons [27]	
	Gpnmb	activators of the pathway that stimulates the development and survival of neurons [28]	
	Atf3	gene activated by the ERK1 and ERK2 cascade and stimulating cell proliferation [29]	
	Trim25	activator of NF-kappa B signaling pathway [30]	canonical NF-kappaB signal transduction
	Ier3	activator of NF-kappa B signaling pathway [31]	
	Hspb1	gene protectes against reactive oxygen species and the elimination of proteins damaged by it [32]	response to reactive oxygen species
	Prkcd	kinase that controls the balance between cell death and survival [33]	
	Stk38l	gene controles microtubule reorganization and neurite growth [34]	regulation of cellular catabolic process
	Snx18	gene regulates the development of the axonal cone [35]	regulation of neurogenesis
	Nab2	gene controls axon growth and dendrite branching [36]	
	Gfap	gene controles maintaining the structure and function of astrocytes [37]	gliogenesis
	Tmbim1	transmembrane subunit of the BAX inhibitor - regulates the level of intracellular calcium, and also blocks the action of the proapoptotic protein Bax [38]	
	Bag3	gene controls the formation of autophagosomes and stabilizes the functioning of mitochondria [39]	negative regulation of apoptotic signaling pathway
	Bdnf	gene plays a vital role in supporting the growth and survival of neurons in the brain	regulation of autophagy
	Mycn	gene acts as a transcription factor, influencing gene expression related to cell growth, proliferation, and differentiation [40]	regeneration

	Chst3	the chondroitin 6-O-sulfotransferase 1 gene, which is responsible for modifications of chondroitin sulfate proteoglycans needed for the regeneration of neuronal processes [41]	
	Creb3l2	transcription factor involved in neuroregeneration, particularly by promoting neurite outgrowth and neuronal differentiation [42]	

Table S1. Lists of the most significant down-regulated differentially expressed genes (DEGs) associated with enriched biological pathways in the IC_vs_EV comparison group.

Up/Down-regulated	Name of the gene	Description	GO_Biological processes
Up-regulated	*Trpv2, Nell2, Thy1, Kiaa0319*	gene's proteins are found in the axonal cone and provide guidance for axon growth [43–46]	axonogenesis
	Rtn4rl1	gene is an active participant in axon regeneration [47]	
	Slit2	gene is responsible for axon navigation, inhibiting its growth in the wrong direction [48]	
	Ptpro	gene controls axon elongation and branching in response to BDNF [49]	
	Robo2	Robo-signaling protein responsible for axon elongation [50]	
	Epb41l3, Epha4, Ephb6, Ephx4	the ephrin family genes are responsible for axon elongation [51]	
	Snap25	synaptovesicle protein [52]	regulation of synapse structure or activity
	Shank2, Shank1	synaptic scaffold proteins, to control receptor positioning [53]	
	Lrfn1, Dlg4, Arhgap33	genes control synaptic plasticity and structure control [54–56]	
	Kidins220, Celsr2, Bcl11a	dendrite formation genes [57–59]	dendrite development
	Nos1	nitric oxide (NO) synthetase, which acts as a signaling messenger in various neural processes [60]	neurotransmitter transport
	Adora2a	receptor that influence the release of various neurotransmitters, including glutamate, dopamine and GABA [61]	
	Slc16a2	thyroid hormone transporter [62]	
	Slc17a6 , Slc17a7	glutamate transporters [63]	

	Slc2a3, Slc2a6	glucose transporters [64]	
	Stxbp5	neurotransmitter exocytosis regulator [65]	
	Iqsec3	gene participates in the regulation of the postsynaptic membrane in GABAergic synapses [66]	postsynapse organization
	Itpka	one of the subunits of postsynaptic kinases that respond to activation of receptors on the postsynaptic membrane [67]	
	Grip2	the major protein of scaffolds on the postsynaptic membrane that controls the location of AMPA-kainate receptors [68]	
	Ppfia2, Ppfia3, Ppfia4, Rims1, Unc13a	proteins form presynaptic terminals by regulating the fusion of synaptic vesicles with the membrane [69]	positive regulation of synaptic transmission
	LRRTM1, Cnksr2, Dact1	genes responsible for synapse structuring and formation [70,71]	
	Syp	gene modulates the function of the synapse, which controls vesicle fusion and neurotransmitter exocytosis [72]	
	Rasgrf1	a protein in the Ras signaling pathway that is responsible for the response to calcium influx at the postsynaptic membrane [73]	
	Gria4, Gria1	glutamate receptor subunits [74]	glutamate receptor signaling pathway
	Grin1, Grin2d	NMDA receptor subunit genes that respond to glutamate [75]	
	Camk1g, Camk2a, Camk2b, Camk2d, Camkk1	Ca2+/calmodulin-dependent protein kinase genes, which are responsible for the membrane depolarization response [76]	regulation of membrane depolarization
	Tspoap1	gene influences the localization of voltage-gated channels [77]	regulation of metal ion transport
	Kcnip1 , Kcnip2, Kcnip4	Potassium ion voltage-gated channel proteins [78]	
	Fxyd6, Fxyd7	proteins that control the work of Na+/K+-ATPase [79]	
	Atp1a3, Atp1b1	Na+/K+-ATPase subunit proteins [80]	
	Atp2b2, Atp2b3, Atp2b4	Plasma membrane Ca2+ transporting ATPase (PMCA2) plays a critical role in neurons by regulating calcium ion concentrations [81]	calcium ion transport

	Rell2	gene for a transmembrane protein that activates Rell signaling [82]	positive regulation of cell-substrate adhesion
	Thy1	CD90 protein is a membrane protein that controls axonal growth by anchoring it to the extracellular matrix [83]	
	Foxg1, Rnf112, Parp6	genes involved in neuronal development [84–86]	positive regulation of nervous system development
	Pdgfb	astrocyte proliferation stimulating protein [87]	gliogenesis
	Bmerb1, Carmil2, Carmil3	proteins that regulate actin polymerization and signaling pathways in neurons [88,89]	actin filament organization
	mTOR	autophagy regulating protein [90]	negative regulation of neuron apoptotic process
	Agap2	gene stimulates neuronal survival processes by activating the PI3K/Akt pathway [91]	
**Down-regulated**	*Hmox1, Serpine1*	hypoxia response protein [92]	response to hypoxia
	Pmaip1	proapoptotic protein, inhibitory anti-apoptotic protein Mlc-1 [93]	
	Limd1	Hif-1alpha inhibitory gene [94]	
	Lif, Vrk2, Sphk1, Klf6	activators of cell survival pathways [95–98]	cellular response to chemical stress
	Fos, Sesn2, Gdf15	a marker of cellular response to stress [99–101]	
	Hsp90b1	heat shock protein that responds to the accumulation of misfolded proteins [102]	
	Rest, Bmp4	protein activated by oxidative stress [103,104]	
	Bdkrb2	bradykinin receptor that triggers neuroinflammation processes [105]	
	Trib1, Fosl1	genes that are activated by different types of stress, such as DNA damage from reactive oxygen species [106,107]	cellular response to oxidative stress
	Nfe2l2, Prdx1, Pex2	genes for proteins that neutralizes reactive oxygen species, thereby protecting cells [108–110]	
	Dnajc3, Pdia4	genes are activated by ER stress [111,112]	response to endoplasmic reticulum stress
	Anxa1, Hells	their expression is increased during apoptosis [113,114]	regulation of apoptotic signaling pathway
	Ier3, Fas, Tnfrsf12a	genes that are activated by any type of stress, stimulating apoptosis [115,116]	

	Tpt1	a gene that controls both cell survival and cell death pathways [117]	
	Lgals3	galectin-3 protein, which is activated by glutamate excitotoxicity and triggers autophagy and apoptosis processes [118]	
	Spry2	gene reacts to a decrease in oxygen by activating cell death processes [119]	
	Klf4	a gene for a protein that enhances the response to oxidative stress, thereby stimulating apoptosis [120]	
	Cdkn1a, Csrp1	a gene that is activated in response to cell damage and stimulates its repair [121,122]	wound healing
	Nrg1	gene actively participates in the development of neurons and in the restoration of synapse function [123]	
	Tfpi2, Rhoc	genes activated by axonal injury [124,125]	
	Fgf2	cell division stimulating growth factor [126]	
	Egr2, Etv5, Nin, Sox10, Sox8	proteins that control neurite elongation [127–130]	regulation of neurogenesis
	Nkx2-2, Notch1	proteins stimulate regeneration [131,132]	
	Sdc4, Cxcl12	genes responsible for intercellular adhesion and migration [133,134]	regulation of cell-cell adhesion
	Has2	gene controls synthesis of hyaluronic threads [20]	extracellular structure organization
	Piezo1	mechanotransduction gene activated by neuronal adhesion [135]	
	Gadd45b, Gadd45g, Gadd45a	genes for subunits of phosphatase that inhibits the MAPK kinase cascade [136]	negative regulation of phosphorylation
	Ppp1r15a	a gene that is activated in response to various types of cellular stress, including oxidative stress [137]	
	Cdkn2c, Cdkn2b	inhibitors of CDK4 and CDK6, thereby inhibiting the cell cycle [138]	

Table S2. Lists of the most significant differentially expressed genes (DEGs) associated with enriched biological pathways in the IC_vs_GL and GL_Mem_vs_GL comparison groups.

Up/Down-regulated	Name of the gene	Description	GO_Biological processes
Up-regulated	*Elk3, Timp1, Anxa2*	genes stimulate wound healing by enhancing proliferation, elongating processes and inhibiting apoptosis [139–141]	wound healing
	Fgr	tyrosine kinase that activates NF-κB and ERK1/2 pathways [142]	phosphatidylinositol 3-kinase/protein kinase B signal transduction
	Txn1	thioredoxin, which acts as an antioxidant, thereby protecting against oxidative stress [143]	
	Axl	Gas6 receptor, activation of which also promotes survival [144]	
	Metrn	gene responsible for the elongation of axons [145]	regulation of axonogenesis
	Crabp2	gene participates in axon regeneration [146]	regulation of axonogenesis<br>cell-substrate adhesion
	Fbln2	a gene encoding an extracellular matrix protein that is involved in the outgrowth of the dendritic tree [147]	
	Lgals1	galectin, which is actively expressed in neurons and is responsible for the interaction between cells of the nervous tissue [148]	cell-substrate adhesion<br>extracellular matrix organisation
	S100a10	it is involved in the regulation of serotonin receptor traffic, glutamatergic transmission and calcium signaling in neurons [149]	
	Fn1	fibronectin, a key component of the brain's extracellular matrix	
	Adamts7	a metalloproteinase that can degrade components of the extracellular matrix [150]	extracellular matrix organisation<br>actin filament organisation
	Vwa1	gene is actively expressed at the ends of neurites and interacts with the extracellular matrix [151]	
	Arpc1b	a protein responsible for the branching of the actin cytoskeleton and thus for the branching of neurites [152]	
	Tpm2	gene controls the formation of axons and dendrites, the elongation of nerve cell processes [153]	actin filament organisation<br>gliogenesis
	Tspo	gene responsible for astrocyte proliferation [154]	
	Olig1	gene activates oligodendrocytes and stimulates the protection of neuronal processes [155]	gliogenesis<br>response to reactive oxygen species
	Nptxr	pentraxin, which controls synapse formation and the flow of ions into neurons [156]	
	Pex5	gene involved in the formation of peroxisomes [157]	response to reactive oxygen species<br>cellular response to oxidative stress
	G6pd	gene involved in cellular respiration and synthesis	
	Coa8	electron transport chain gene cytochrome c oxidase-binding [158]	

	Lcn2	gene participates in the control of neuronal responses to external stimuli [159]	cellular response to oxidative stress<br>reactive oxygen species metabolic process
	Spp1	gene is associated with cell survival and axon reorganization upon injury[144]	response to axon injury
	Tesc	gene controls the entry of metal ions into neurons, especially calcium ions, by binding to it [160,161]	regulation of metal ion transport
	Hspa2	heat shock protein gene, which is involved in ion transport by binding to the CatSper ion channel [161]	regulation of metal ion transport<br>peptide hormone secretion
	Kcnip2	Potassium ion voltage-gated channel [78]	
	Best1	calcium-activated anion channel, meaning it allows chloride ions to pass through cell membranes in response to changes in intracellular calcium levels [162]	
	Fxyd5	gene regulates the work of Na+/K+-ATPase [163]	
	Rbp4	gene participates in the transport of retinoic acid [164]	
	Npy	a neuropeptide essential for the survival of neurons [165]	peptide hormone secretion<br>regulation of membrane depolarisation
	Scn1b	voltage-gated sodium channel beta-1 subunit (VGSC) [166]	
	Auts2, Tnr, Septin7	genes regulate of axon and neurite growth [167–169]	axonogenesis
**Down-regulated**	*Ttc3 , Vangl2 , Nfib*	genes inhibit the emergence of neurites and the elongation of axons [170–172]	axonogenesis<br>regulation of cellular response to stress
	Kif5c , Kif5b, Mapt	genes responsible for transport in axons and dendrites [173,174]	
	Tmem33	an ER stress-induced molecule that modulates the unfolded protein response signaling cascade leading to apoptosis [175]	
	Pum2	gene responsible for axon regeneration in response to stress [176]	regulation of cellular response to stress<br>dendrite development
	Hgf	gene stimulates neuronal survival in response to injury [177]	
	Nfe2l1	gene activated by ER stress [178]	
	Nek1	gene plays a key role in the response to DNA damage [179]	
	Foxo1	one of the key genes in the response to oxidative stress [180]	
	Ino80	gene responsible for the repair of double-strand DNA breaks [181]	
	USP13	gene plays a role in various cellular processes in neurons, including regulation of protein degradation and axonal degeneration [182]	

	Mef2c	a gene encoding a protein that regulates the formation of dendritic spines, which stimulates the elimination of spines during excitotoxicity [183]	
	Pak3	gene participates in the formation of dendritic spines [184]	dendrite development
	Ppp1r9a	gene responsible for the creation of protein scaffolds in the synapse [185]	regulation of synapse organization
	Tnik , *Zfp804a* , *Tanc2*	genes responsible for regulating the structure and function of synapses [186–188]	regulation of synapse organization
	Ntrk2 , *Ephb1* , *Sema5a* , *Cxcl12*	genes are necessary for the correct direction of axon growth [189–191]	axon guidance
	Mycbp2 [192]	gene is involved in axon degradation	axon guidance
	Atp2b4, *Atp2b1*, *Atp1a2*, *Ryr2*, *Camk4*	genes of ion transporters that control the transport of calcium ions into the cytoplasm of neurons	regulation of calcium ion transport
	Baz1a , *Kdm5a* , *Smarca2*	genes that control DNA remodeling [193–195]	negative regulation of gene expression, epigenetic
	Limch1, *Pde4d*	gene play a role in regulating cell motility [196,197]	actin-mediated cell contraction
	Cacna1c, *Cacna1d*, *Cacna2d1*	subunits of voltage-dependent calcium channel [198]	calcium ion transmembrane transport via high voltage-gated calcium channel
	Kif3a, *Map1a*	genes of motor proteins responsible for cargo transport from axons and dendrites [199,200]	axo-dendritic protein transport

Table S3. Lists of the most significant differentially expressed genes (DEGs) associated with enriched biological pathways in the GL_EV_vs_GL comparison group.

References:

1. Saganich, M.J.; Machado, E.; Rudy, B. Differential expression of genes encoding subthreshold-operating voltage-gated K+ channels in brain. *J. Neurosci.* **2001**, *21*, 4609–4624. https://doi.org/10.1523/jneurosci.21-13-04609.2001.
2. Xu, Q.; Fan, G.; Shao, S. Role of TNFRSF12A in cell proliferation, apoptosis, and proinflammatory cytokine expression by regulating the MAPK and NF-κB pathways in thyroid cancer cells. *Cytokine* **2025**, *186*, 156841. https://doi.org/10.1016/j.cyto.2024.156841.
3. Ashutosh Chao, C.; Borgmann, K.; Brew, K.; Ghorpade, A. Tissue inhibitor of metalloproteinases-1 protects human neurons from staurosporine and HIV-1-induced apoptosis: Mechanisms and relevance to HIV-1-associated dementia. *Cell Death Dis.* **2012**, *3*, e332–e332.
4. Gauthier-Kemper, A.; Alonso, M.S.; Sündermann, F.; Niewidok, B.; Fernandez, M.-P.; Bakota, L.; Heinisch, J.J.; Brandt, R. Annexins A2 and A6 interact with the extreme N terminus of tau and thereby

contribute to tau's axonal localization. *J. Biol. Chem.* **2018**, *293*, 8065–8076. https://doi.org/10.1074/jbc.ra117.000490.
5. Mandal, P.; Belapurkar, V.; Nair, D.; Ramanan, N. Vinculin-mediated axon growth requires interaction with actin but not talin in mouse neocortical neurons. *Cell. Mol. Life Sci.* **2021**, *78*, 5807–5826. https://doi.org/10.1007/s00018-021-03879-7.
6. Buttiglione, M.; Revest, J.-M.; Rougon, G.; Faivre-Sarrailh, C. F3 neuronal adhesion molecule controls outgrowth and fasciculation of cerebellar granule cell neurites: A cell-type-specific effect mediated by the Ig-like domains. *Mol. Cell. Neurosci.* **1996**, *8*, 53–69. https://doi.org/10.1006/mcne.1996.0043.
7. Istiaq, A.; Ohta, K. A review on Tsukushi: Mammalian development, disorders, and therapy. *J. Cell Commun. Signal.* **2022**, *16*, 505–513. https://doi.org/10.1007/s12079-022-00669-z.
8. Gimenez-Cassina, A.; Lim, F.; Cerrato, T.; Palomo, G.M.; Diaz-Nido, J. Mitochondrial hexokinase II promotes neuronal survival and acts downstream of glycogen synthase kinase-3. *J. Biol. Chem.* **2009**, *284*, 3001–3011. https://doi.org/10.1074/jbc.m808698200.
9. Nitti, M.; Piras, S.; Brondolo, L.; Marinari, U.M.; Pronzato, M.A.; Furfaro, A.L. Heme oxygenase 1 in the nervous system: Does it favor neuronal cell survival or induce neurodegeneration? *Int. J. Mol. Sci.* **2018**, *19*, 2260. https://doi.org/10.3390/ijms19082260.
10. Dziennis, S.; Alkayed, N.J. Role of signal transducer and activator of transcription 3 in neuronal survival and regeneration. *Rev. Neurobiol.* **2008**, *19*, 341–362. https://doi.org/10.1515/revneuro.2008.19.4-5.341.
11. Yoshida, T.; Shiroshima, T.; Lee, S.-J.; Yasumura, M.; Uemura, T.; Chen, X.; Iwakura, Y.; Mishina, M. Interleukin-1 receptor accessory protein organizes neuronal synaptogenesis as a cell adhesion molecule. *J. Neurosci.* **2012**, *32*, 2588–2600. https://doi.org/10.1523/jneurosci.4637-11.2012.
12. Dzwonek, J.; Wilczyński, G.M. CD44: Molecular interactions, signaling and functions in the nervous system. *Front. Cell. Neurosci.* **2015**, *9*, 175.
13. Ji, E.; Zhang, Y.; Li, Z.; Wei, L.; Wu, Z.; Li, Y.; Yu, X.; Song, T.-J. The Chemokine CCL2 Promotes Excitatory Synaptic Transmission in Hippocampal Neurons via GluA1 Subunit Trafficking. *Neurosci. Bull.* **2024**, *40*, 1649–1666. https://doi.org/10.1007/s12264-024-01236-9.
14. Lieberam, I.; Agalliu, D.; Nagasawa, T.; Ericson, J.; Jessell, T.M. A Cxcl12-Cxcr4 Chemokine signaling pathway defines the initial trajectory of mammalian motor axons. *Neuron* **2005**, *47*, 667–679. https://doi.org/10.1016/j.neuron.2005.08.011.
15. Caminero, A.; Comabella, M.; Montalban, X. Role of tumour necrosis factor (TNF)-α and *TNFRSF1A* R92Q mutation in the pathogenesis of TNF receptor-associated periodic syndrome and multiple sclerosis. *Clin. Exp. Immunol.* **2011**, *166*, 338–345. https://doi.org/10.1111/j.1365-2249.2011.04484.x.
16. Sun, Z.; Xu, X.; He, J.; Murray, A.; Sun, M.-A.; Wei, X.; Wang, X.; McCoig, E.; Xie, E.; Jiang, X.; et al. EGR1 recruits TET1 to shape the brain methylome during development and upon neuronal activity. *Nat. Commun.* **2019**, *10*, 3892. https://doi.org/10.1038/s41467-019-11905-3.
17. Yang, N.J.; Isensee, J.; Neel, D.; Liu, S.M.; Zhang, H.X.B.; Belu, A.; Palan, S.; Kennedy-Curran, A.; Röderer, P.; Nitzsche, A.; et al. Anthrax Toxin as a Molecular Platform to Target Nociceptive Neurons and Modulate Pain. *bioRxiv* **2020**, https://doi.org/10.1101/2020.03.28.004150.
18. Mohamedi, Y.; Fontanil, T.; Cobo, T.; Cal, S.; Obaya, A.J. New insights into adamts metalloproteases in the central nervous system. *Biomolecules* **2020**, *10*, 403. https://doi.org/10.3390/biom10030403.
19. Li, J.; Parker, B.; Martyn, C.; Natarajan, C.; Guo, J. The PMP22 gene and its related diseases. *Mol. Neurobiol.* **2013**, *47*, 673–698. https://doi.org/10.1007/s12035-012-8370-x.
20. Camenisch, T.D.; Spicer, A.P.; Brehm-Gibson, T.; Biesterfeldt, J.; Augustine, M.L.; Calabro, A.; Kubalak, S.; Klewer, S.E.; McDonald, J.A. Disruption of hyaluronan synthase-2 abrogates normal cardiac morphogenesis and hyaluronan-mediated transformation of epithelium to mesenchyme. *J. Clin. Investig.* **2000**, *106*, 349–360. https://doi.org/10.1172/jci10272.
21. Joy, M.T.; Vrbova, G.; Dhoot, G.K.; Anderson, P.N. Sulf1 and Sulf2 expression in the nervous system and its role in limiting neurite outgrowth in vitro. *Exp. Neurol.* **2015**, *263*, 150–160. https://doi.org/10.1016/j.expneurol.2014.10.011.

22. Cao, Y.; Jin, H.-G.; Ma, H.-H.; Zhao, Z.-H. Comparative analysis on genome-wide DNA methylation in longissimus dorsi muscle between Small Tailed Han and DorperSmall Tailed Han crossbred sheep. *Asian-Australas. J. Anim. Sci.* **2017**, *30*, 1529–1539. https://doi.org/10.5713/ajas.17.0154.
23. Leung, G.; Zhou, Y.; Ostrowski, P.; Mylvaganam, S.; Boroumand, P.; Mulder, D.J.; Guo, C.; Muise, A.M.; Freeman, S.A. ARPC1B binds WASP to control actin polymerization and curtail tonic signaling in B cells. *J. Clin. Investig.* **2021**, *6*, e149376. https://doi.org/10.1172/jci.insight.149376.
24. Kim, H.-R.; Kwon, M.-S.; Lee, S.; Mun, Y.; Lee, K.-S.; Kim, C.-H.; Na, B.-R.; Kim, B.N.R.; Piragyte, I.; Lee, H.-S.; et al. TAGLN2 polymerizes G-actin in a low ionic state but blocks Arp2/3-nucleated actin branching in physiological conditions. *Sci. Rep.* **2018**, *8*, 5503. https://doi.org/10.1038/s41598-018-23816-2.
25. Chen, Z.; Wang, C.; Li, M.; Cai, S.; Liu, X. SPRED3 regulates the NF-κB signaling pathway in thyroid cancer and promotes the proliferation. *Sci. Rep.* **2024**, *14*, 20506. https://doi.org/10.1038/s41598-024-61075-6.
26. Pan, A.L.; Audrain, M.; Sakakibara, E.; Joshi, R.; Zhu, X.; Wang, Q.; Wang, M.; Beckmann, N.D.; Schadt, E.E.; Gandy, S.; et al. Dual-specificity protein phosphatase 6 (DUSP6) overexpression reduces amyloid load and improves memory deficits in male 5xFAD mice. *Front. Aging Neurosci.* **2024**, *16*, 1400447. https://doi.org/10.3389/fnagi.2024.1400447.
27. Huang, S.; Liu, F.; Niu, Q.; Li, Y.; Liu, C.; Zhang, L.; Ni, D.; Pu, X. GLIPR-2 Overexpression in HK-2 Cells Promotes Cell EMT and Migration through ERK1/2 Activation. *PLoS ONE* **2013**, *8*, e58574. https://doi.org/10.1371/journal.pone.0058574.
28. Satoh, J.-I.; Kino, Y.; Yanaizu, M.; Ishida, T.; Saito, Y. Microglia express GPNMB in the brains of Alzheimer's disease and Nasu-Hakola disease. *Intractable Rare Dis. Res.* **2019**, *8*, 120–128. https://doi.org/10.5582/irdr.2019.01049.
29. Seijffers, R.; Zhang, J.; Matthews, J.C.; Chen, A.; Tamrazian, E.; Babaniyi, O.; Selig, M.; Hynynen, M.; Woolf, C.J.; Brown, R.H. ATF3 expression improves motor function in the ALS mouse model by promoting motor neuron survival and retaining muscle innervation. *Proc. Natl. Acad. Sci. USA* **2014**, *111*, 1622–1627. https://doi.org/10.1073/pnas.1314826111.
30. Liu, Y.; Liu, K.; Huang, Y.; Sun, M.; Tian, Q.; Zhang, S.; Qin, Y. TRIM25 Promotes TNF-α–Induced NF-κB Activation through Potentiating the K63-Linked Ubiquitination of TRAF2. *J. Immunol.* **2020**, *204*, 1499–1507.
31. Ye, J.; Zhang, Y.; Cai, Z.; Jiang, M.; Li, B.; Chen, G.; Zeng, Y.; Liang, Y.; Wu, S.; Wang, Z.; et al. Increased expression of immediate early response gene 3 protein promotes aggressive progression and predicts poor prognosis in human bladder cancer. *BMC Urol.* **2018**, *18*, 82. https://doi.org/10.1186/s12894-018-0388-6.
32. Srivastava, A.K.; Renusch, S.R.; Naiman, N.E.; Gu, S.; Sneh, A.; Arnold, W.D.; Sahenk, Z.; Kolb, S.J. Mutant HSPB1 overexpression in neurons is sufficient to cause age-related motor neuronopathy in mice. *Neurobiol. Dis.* **2012**, *47*, 163–173. https://doi.org/10.1016/j.nbd.2012.03.035.
33. Jin, H.; Kanthasamy, A.; Anantharam, V.; Rana, A.; Kanthasamy, A.G. Transcriptional regulation of pro-apoptotic protein kinase Cδ: Implications for oxidative stress-induced neuronal cell death. *J. Biol. Chem.* **2011**, *286*, 19840–19859.
34. Léger, H.; Santana, E.; Leu, N.A.; Smith, E.T.; Beltran, W.A.; Aguirre, G.D.; Luca, F.C. Ndr kinases regulate retinal interneuron proliferation and homeostasis. *Sci. Rep.* **2018**, *8*, 12544. https://doi.org/10.1038/s41598-018-30492-9.
35. Nakazawa, S.; Gotoh, N.; Matsumoto, H.; Murayama, C.; Suzuki, T.; Yamamoto, T. Expression of sorting Nexin 18 (SNX18) is dynamically regulated in developing Spinal Motor Neurons. *J. Histochem. Cytochem.* **2011**, *59*, 202–213. https://doi.org/10.1369/0022155410392231.
36. Corgiat, E.B.; List, S.M.; Rounds, J.C.; Corbett, A.H.; Moberg, K.H. The RNA-binding protein Nab2 regulates the proteome of the developing Drosophila brain. *J. Biol. Chem.* **2021**, *297*, 100877. https://doi.org/10.1016/j.jbc.2021.100877.
37. Yang, Z.; Wang, K.K. Glial fibrillary acidic protein: From intermediate filament assembly and gliosis to neurobiomarker. *Trends Neurosci.* **2015**, *38*, 364–374. https://doi.org/10.1016/j.tins.2015.04.003.

38. Cai, J.; Gao, L.; Wang, Y.; Li, Y.; Ye, Z.; Tong, S.; Yan, T.; Sun, Q.; Xu, Y.; Jiang, H.; et al. TMBIM1 promotes proliferation and attenuates apoptosis in glioblastoma cells by targeting the p38 MAPK signalling pathway. *Transl. Oncol.* **2022**, *19*, 101391. https://doi.org/10.1016/j.tranon.2022.101391.
39. Santoro, A.; Nicolin, V.; Florenzano, F.; Rosati, A.; Capunzo, M.; Nori, S.L. BAG3 is involved in neuronal differentiation and migration. *Cell Tissue Res.* **2017**, *368*, 249–258. https://doi.org/10.1007/s00441-017-2570-7.
40. Kerosuo, L.; Neppala, P.; Hsin, J.; Mohlin, S.; Vieceli, F.M.; Török, Z.; Laine, A.; Westermarck, J.; Bronner, M.E. Enhanced expression of MycN/CIP2A drives neural crest toward a neural stem cell-like fate: Implications for priming of neuroblastoma. *Proc. Natl. Acad. Sci. USA* **2018**, *115*, E7351–E7360. https://doi.org/10.1073/pnas.1800039115.
41. Pantazopoulos, H.; Woo, T.-U.W.; Lim, M.P.; Lange, N.; Berretta, S. Extracellular matrix-glial abnormalities in the amygdala and entorhinal cortex of subjects diagnosed with schizophrenia. *Arch. Gen. Psychiatry* **2010**, *67*, 155–166. https://doi.org/10.1001/archgenpsychiatry.2009.196.
42. Sampieri, L.; Funes Chabán, M.; Di Giusto, P.; Rozés-Salvador, V.; Alvarez, C. CREB3L2 Modulates Nerve Growth Fac-tor-Induced Cell Differentiation. *Front. Mol. Neurosci.* **2021**, *14*, 650338.
43. Shibasaki, K.; Murayama, N.; Ono, K.; Ishizaki, Y.; Tominaga, M. TRPV2 Enhances axon outgrowth through its activation by membrane stretch in developing sensory and motor neurons. *J. Neurosci.* **2010**, *30*, 4601–4612. https://doi.org/10.1523/jneurosci.5830-09.2010.
44. Franquinho, F.; Nogueira-Rodrigues, J.; Duarte, J.M.; Esteves, S.S.; Carter-Su, C.; Monaco, A.P.; Molnár, Z.; Velayos-Baeza, A.; Brites, P.; Sousa, M.M. The dyslexia-susceptibility protein kiaa0319 inhibits axon growth through smad2 signaling. *Cereb. Cortex* **2017**, *27*, 1732–1747. https://doi.org/10.1093/cercor/bhx023.
45. Jósvay, K.; Winter, Z.; Katona, R.L.; Pecze, L.; Marton, A.; Buhala, A.; Szakonyi, G.; Oláh, Z.; Vizler, C. Besides neuro-imaging, the Thy1-YFP mouse could serve for visualizing experimental tumours, inflammation and wound-healing. *Sci. Rep.* **2014**, *4*, 6776. https://doi.org/10.1038/srep06776.
46. Kim, H.R.; Kim, D.H.; An, J.Y.; Kang, D.; Park, J.W.; Hwang, E.M.; Seo, E.J.; Jang, I.H.; Ha, C.M.; Lee, B.J. NELL2 function in axon development of hippocampal neurons. *Mol. Cells* **2020**, *43*, 581–589. https://doi.org/10.14348/molcells.2020.0032.
47. Wang, J.; Miao, Y.; Wicklein, R.; Sun, Z.; Wang, J.; Jude, K.M.; Fernandes, R.A.; Merrill, S.A.; Wernig, M.; Garcia, K.C.; et al. RTN4/NoGo-receptor binding to BAI adhesion-GPCRs regulates neuronal development. *Cell* **2021**, *184*, 5869–5885.
48. Sherchan, P.; Travis, Z.D.; Tang, J.; Zhang, J.H. The potential of Slit2 as a therapeutic target for central nervous system disorders. *Expert Opin. Ther. Targets* **2020**, *24*, 805–818. https://doi.org/10.1080/14728222.2020.1766445.
49. Gatto, G.; Dudanova, I.; Suetterlin, P.; Davies, A.M.; Drescher, U.; Bixby, J.L.; Klein, R. Protein tyrosine phosphatase receptor type O inhibits trigeminal axon growth and branching by repressing TrkB and ret signaling. *J. Neurosci.* **2013**, *33*, 5399–5410. https://doi.org/10.1523/jneurosci.4707-12.2013.
50. Kim, M.; Roesener, A.P.; Mendonca, P.R.; Mastick, G.S. Robo1 and Robo2 have distinct roles in pioneer longitudinal axon guidance. *Dev. Biol.* **2011**, *358*, 181–188. https://doi.org/10.1016/j.ydbio.2011.07.025.
51. Orioli, D.; Klein, R. The eph receptor family: Axonal guidance by contact repulsion. *Trends Genet.* **1997**, *13*, 354–359. https://doi.org/10.1016/s0168-9525(97)01220-1.
52. Corradini, I.; Verderio, C.; Sala, M.; Wilson, M.C.; Matteoli, M. SNAP-25 in neuropsychiatric disorders. *Ann. N.Y. Acad. Sci.* **2009**, *1152*, 93–99.
53. Ha, S.; Lee, D.; Cho, Y.S.; Chung, C.; Yoo, Y.-E.; Kim, J.; Lee, J.; Kim, W.; Kim, H.; Bae, Y.C.; et al. Cerebellar shank2 regulates excitatory synapse density, motor coordination, and specific repetitive and anxiety-like behaviors. *J. Neurosci.* **2016**, *36*, 12129–12143. https://doi.org/10.1523/jneurosci.1849-16.2016.
54. de Wit, J.; Ghosh, A. Control of neural circuit formation by leucine-rich repeat proteins. *Trends Neurosci.* **2014**, *37*, 539–550. https://doi.org/10.1016/j.tins.2014.07.004.
55. Bustos, F.J.; Ampuero, E.; Jury, N.; Aguilar, R.; Falahi, F.; Toledo, J.; Ahumada, J.; Lata, J.; Cubillos, P.; Henríquez, B.; et al. Epigenetic editing of the Dlg4/PSD95 gene improves cognition in aged and Alzheimer's disease mice. *Brain* **2017**, *140*, 3252–3268. https://doi.org/10.1093/brain/awx272.

56. Niftullayev, S.; Lamarche-Vane, N. Regulators of rho GTPases in the nervous system: Molecular implication in axon guidance and neurological disorders. *Int. J. Mol. Sci.* **2019**, *20*, 1497. https://doi.org/10.3390/ijms20061497.
57. Chen, Y.; Fu, W.-Y.; Ip, J.P.K.; Ye, T.; Fu, A.K.Y.; Chao, M.V.; Ip, N.Y. Ankyrin repeat-rich membrane spanning protein (Kidins220) is required for neurotrophin and ephrin receptor-dependent dendrite development. *J. Neurosci.* **2012**, *32*, 8263–8269. https://doi.org/10.1523/jneurosci.1264-12.2012.
58. Shima, Y.; Kawaguchi, S.-Y.; Kosaka, K.; Nakayama, M.; Hoshino, M.; Nabeshima, Y.; Hirano, T.; Uemura, T. Opposing roles in neurite growth control by two seven-pass transmembrane cadherins. *Nat. Neurosci.* **2007**, *10*, 963–969. https://doi.org/10.1038/nn1933.
59. Du, H.; Wang, Z.; Guo, R.; Yang, L.; Liu, G.; Zhang, Z.; Xu, Z.; Tian, Y.; Yang, Z.; Li, X.; et al. Transcription factors *Bcl11a* and *Bcl11b* are required for the production and differentiation of cortical projection neurons. *Cereb. Cortex* **2022**, *32*, 3611–3632. https://doi.org/10.1093/cercor/bhab437.
60. Gutiérrez, H.C.; O'lEary, A.; Freudenberg, F.; Fedele, G.; Wilkinson, R.; Markham, E.; van Eeden, F.; Reif, A.; Norton, W.H. Nitric oxide interacts with monoamine oxidase to modulate aggression and anxiety-like behaviour. *Eur. Neuropsychopharmacol.* **2020**, *30*, 30–43. https://doi.org/10.1016/j.euroneuro.2017.09.004.
61. Pinna, A.; Serra, M.; Marongiu, J.; Morelli, M. Pharmacological interactions between adenosine A2A receptor antagonists and different neurotransmitter systems. *Park. Relat. Disord.* **2020**, *80*, S37–S44. https://doi.org/10.1016/j.parkreldis.2020.10.023.
62. Tan, J.; Xiao, Y.; Kong, F.; Qian, J.; Zhu, A.; Yan, C. Structural insights into thyroid hormone transporter MCT8. *Nat. Commun.* **2025**, *16*, 2958. https://doi.org/10.1038/s41467-025-58131-8.
63. Eriksen, J.; Li, F.; Edwards, R.H. The mechanism and regulation of vesicular glutamate transport: Coordination with the synaptic vesicle cycle. *Biochim. Et Biophys. Acta-Biomembr.* **2020**, *1862*, 183259. https://doi.org/10.1016/j.bbamem.2020.183259.
64. Pizzagalli, M.D.; Bensimon, A.; Superti-Furga, G. A guide to plasma membrane solute carrier proteins. *FEBS J.* **2021**, *288*, 2784–2835. https://doi.org/10.1111/febs.15531.
65. Batten, S.R.; Matveeva, E.A.; Whiteheart, S.W.; Vanaman, T.C.; Gerhardt, G.A.; Slevin, J.T. Linking kindling to increased glutamate release in the dentate gyrus of the hippocampus through the STXBP5/tomosyn-1 gene. *Brain Behav.* **2017**, *7*, e00795. https://doi.org/10.1002/brb3.795.
66. Um, J.W.; Choii, G.; Park, D.; Kim, D.; Jeon, S.; Kang, H.; Mori, T.; Papadopoulos, T.; Yoo, T.; Lee, Y.; et al. IQ Motif and SEC7 domain-containing protein 3 (IQSEC3) interacts with gephyrin to promote inhibitory synapse formation. *J. Biol. Chem.* **2016**, *291*, 10119–10130. https://doi.org/10.1074/jbc.m115.712893.
67. Köster, J.-D.; Leggewie, B.; Blechner, C.; Brandt, N.; Fester, L.; Rune, G.; Schweizer, M.; Kindler, S.; Windhorst, S. Inositol-1,4,5-trisphosphate-3-kinase-A controls morphology of hippocampal dendritic spines. *Cell. Signal.* **2016**, *28*, 83–90. https://doi.org/10.1016/j.cellsig.2015.10.016.
68. Matt, L.; Kim, K.; Chowdhury, D.; Hell, J.W. Role of palmitoylation of postsynaptic proteins in promoting synaptic plasticity. *Front. Mol. Neurosci.* **2019**, *12*, 8. https://doi.org/10.3389/fnmol.2019.00008.
69. Paul, M.S.; Michener, S.L.; Pan, H.; Chan, H.; Pfliger, J.M.; Rosenfeld, J.A.; Lerma, V.C.; Tran, A.; Longley, M.A.; Lewis, R.A.; et al. A syndromic neurodevelopmental disorder caused by rare variants in PPFIA3. *Am. J. Hum. Genet.* **2024**, *111*, 96–118. https://doi.org/10.1016/j.ajhg.2023.12.004.
70. Okerlund, N.D.; Kivimäe, S.; Tong, C.K.; Peng, I.-F.; Ullian, E.M.; Cheyette, B.N.R. Dact1 is a postsynaptic protein required for dendrite, spine, and excitatory synapse development in the mouse forebrain. *J. Neurosci.* **2010**, *30*, 4362–4368. https://doi.org/10.1523/jneurosci.0354-10.2010.
71. Zhang, Y.; Yu, T.; Li, N.; Wang, J.; Wang, J.; Ge, Y.; Yao, R. Psychomotor development and attention problems caused by a splicing variant of CNKSR2. *BMC Med. Genom.* **2020**, *13*, 182. https://doi.org/10.1186/s12920-020-00844-4.
72. Kwon, S.E.; Chapman, E.R. Synaptophysin Regulates the Kinetics of Synaptic Vesicle Endocytosis in Central Neurons. *Neuron* **2011**, *70*, 847–854. https://doi.org/10.1016/j.neuron.2011.04.001.
73. Barman, A.; Assmann, A.; Richter, S.; Soch, J.; Schã¼Tze, H.; Wã¼Stenberg, T.; Deibele, A.; Klein, M.; Richter, A.; Behnisch, G.; et al. Genetic variation of the RASGRF1 regulatory region affects human

hippocampus-dependent memory. *Front. Hum. Neurosci.* **2014**, *8*, 260. https://doi.org/10.3389/fnhum.2014.00260.
74. Ismail, V.; Zachariassen, L.G.; Godwin, A.; Sahakian, M.; Ellard, S.; Stals, K.L.; Baple, E.; Brown, K.T.; Foulds, N.; Wheway, G.; et al. Identification and functional evaluation of GRIA1 missense and truncation variants in individuals with ID: An emerging neurodevelopmental syndrome. *Am. J. Hum. Genet.* **2022**, *109*, 1217–1241. https://doi.org/10.1016/j.ajhg.2022.05.009.
75. Camp, C.R.; Yuan, H. GRIN2D/GluN2D NMDA receptor: Unique features and its contribution to pediatric developmental and epileptic encephalopathy. *Eur. J. Paediatr. Neurol.* **2020**, *24*, 89–99. https://doi.org/10.1016/j.ejpn.2019.12.007.
76. Rigter, P.M.; de Konink, C.; Dunn, M.J.; Onori, M.P.; Humberson, J.B.; Thomas, M.; Barnes, C.; Prada, C.E.; Weaver, K.N.; Ryan, T.D.; et al. Role of CAMK2D in neurodevelopment and associated conditions. *Am. J. Hum. Genet.* **2024**, *111*, 364–382. https://doi.org/10.1016/j.ajhg.2023.12.016.
77. Suthar, S.K.; Alam, M.M.; Lee, J.; Monga, J.; Joseph, A.; Lee, S.-Y. Bioinformatic Analyses of Canonical Pathways of TSPOAP1 and its Roles in Human Diseases. *Front. Mol. Biosci.* **2021**, *8*, 667947. https://doi.org/10.3389/fmolb.2021.667947.
78. Wu, L.-Y.; Song, Y.-J.; Zhang, C.-L.; Liu, J. Kv Channel-Interacting Proteins in the Neurological and Cardiovascular Systems: An Updated Review. *Cells* **2023**, *12*, 1894. https://doi.org/10.3390/cells12141894.
79. Geering, K. FXYD proteins: New regulators of Na-K-ATPase. *Am. J. Physiol.-Physiol.* **2006**, *290*, F241–F250. https://doi.org/10.1152/ajprenal.00126.2005.
80. Murata, K.; Kinoshita, T.; Ishikawa, T.; Kuroda, K.; Hoshi, M.; Fukazawa, Y. Region- and neuronal-subtype-specific expression of Na,K-ATPase alpha and beta subunit isoforms in the mouse brain. *J. Comp. Neurol.* **2020**, *528*, 2654–2678.
81. Strehler, E.E.; Thayer, S.A. Evidence for a role of plasma membrane calcium pumps in neurodegenerative disease: Recent developments. *Neurosci. Lett.* **2018**, *663*, 39–47. https://doi.org/10.1016/j.neulet.2017.08.035.
82. Cusick, J.K.; Alcaide, J.; Shi, Y. The RELT Family of Proteins: An Increasing Awareness of Their Importance for Cancer, the Immune System, and Development. *Biomedicines* **2023**, *11*, 2695. https://doi.org/10.3390/biomedicines11102695.
83. Ilic, K.; Auer, B.; Mlinac-Jerkovic, K.; Herrera-Molina, R. Neuronal signaling by thy-1 in nanodomains with specific ganglioside composition: Shall we open the door to a new complexity? *Front. Cell Dev. Biol.* **2019**, *7*, 27. https://doi.org/10.3389/fcell.2019.00027.
84. Huang, J.Y.; Wang, K.; Vermehren-Schmaedick, A.; Adelman, J.P.; Cohen, M.S. PARP6 is a Regulator of Hippocampal Dendritic Morphogenesis. *Sci. Rep.* **2016**, *6*, 18512. https://doi.org/10.1038/srep18512.
85. Tsou, J.-H.; Yang, Y.-C.; Pao, P.-C.; Lin, H.-C.; Huang, N.-K.; Lin, S.-T.; Hsu, K.-S.; Yeh, C.-M.; Lee, K.-H.; Kuo, C.-J.; et al. Important Roles of Ring Finger Protein 112 in Embryonic Vascular Development and Brain Functions. *Mol. Neurobiol.* **2017**, *54*, 2286–2300. https://doi.org/10.1007/s12035-016-9812-7.
86. Lin, T.-Y.; Wong, L.-C.; Hou, P.-S.; Wu, C.-K.; Cheng, H.-Y.; Zhao, H.-J.; Tung, C.-Y.; Lee, M.-H.; Lee, W.-T.; Tsai, J.-W. Functional defects in FOXG1 variants predict the severity of brain anomalies in FOXG1 syndrome. *Mol. Psychiatry* **2025**, *30*, 1–12. https://doi.org/10.1038/s41380-025-03077-y.
87. Funa, K.; Sasahara, M. The roles of PDGF in development and during neurogenesis in the normal and diseased nervous system. *J. Neuroimmune Pharmacol.* **2014**, *9*, 168–181. https://doi.org/10.1007/s11481-013-9479-z.
88. Brawley, C.M.; Uysal, S.; A Kossiakoff, A.; Rock, R.S. Characterization of engineered actin binding proteins that control filament assembly and structure. *PLoS ONE* **2010**, *5*, e13960. https://doi.org/10.1371/journal.pone.0013960.
89. Stark, B.C.; Lanier, M.H.; Cooper, J.A. CARMIL family proteins as multidomain regulators of actin-based motility. *Mol. Biol. Cell* **2017**, *28*, 1713–1723. https://doi.org/10.1091/mbc.e17-01-0019.
90. Altas, B.; Romanowski, A.J.; Bunce, G.W.; Poulopoulos, A. Neuronal mTOR Outposts: Implications for Translation, Signaling, and Plasticity. *Front. Cell. Neurosci.* **2022**, *16*, 853634. https://doi.org/10.3389/fncel.2022.853634.

91. Chouinard, F.C.; Davis, L.; Gilbert, C.; Bourgoin, S.G. Functional Role of AGAP2/PIKE-A in Fcγ Receptor-Mediated Phag-ocytosis. *Cells* **2023**, *12*, 72.
92. Zhang, L.; Cao, Y.; Guo, X.; Wang, X.; Han, X.; Kanwore, K.; Hong, X.; Zhou, H.; Gao, D. Hypoxia-induced ROS aggravate tumor progression through HIF-1α-SERPINE1 signaling in glioblastoma. *J. Zhejiang Univ. Sci. B* **2023**, *24*, 32–49. https://doi.org/10.1631/jzus.b2200269.
93. Chen, H.; Ma, D.; Yue, F.; Qi, Y.; Dou, M.; Cui, L.; Xing, Y. The Potential Role of Hypoxia-Inducible Factor-1 in the Progression and Therapy of Central Nervous System Diseases. *Curr. Neuropharmacol.* **2021**, *20*, 1651–1666. https://doi.org/10.2174/1570159x19666210729123137.
94. Foxler, D.E.; Bridge, K.S.; Foster, J.G.; Grevitt, P.; Curry, S.; Shah, K.M.; Davidson, K.M.; Nagano, A.; Gadaleta, E.; Rhys, H.I.; Kennedy, P.T.; et al. A HIF–LIMD 1 negative feedback mechanism mitigates the pro-tumorigenic effects of hypoxia. *EMBO Mol. Med.* **2018**, *10*, e8304.
95. Salma, J.; McDermott, J.C. Suppression of a MEF2-KLF6 survival pathway by PKA signaling promotes apoptosis in embryonic hippocampal neurons. *J. Neurosci.* **2012**, *32*, 2790–2803. https://doi.org/10.1523/jneurosci.3609-11.2012.
96. Bryan, L.; Kordula, T.; Spiegel, S.; Milstien, S. Regulation and functions of sphingosine kinases in the brain. *Biochim. Et Biophys. Acta-Mol. Cell Biol. Lipids* **2008**, *1781*, 459–466. https://doi.org/10.1016/j.bbalip.2008.04.008.
97. Li, M.; Yue, W. VRK2, a Candidate Gene for Psychiatric and Neurological Disorders. *Complex Psychiatry* **2018**, *4*, 119–133. https://doi.org/10.1159/000493941.
98. Takata, S.; Sakata-Haga, H.; Shimada, H.; Tsukada, T.; Sakai, D.; Shoji, H.; Tomosugi, M.; Nakamura, Y.; Ishigaki, Y.; Iizuka, H.; et al. LIF–IGF Axis Contributes to the Proliferation of Neural Progenitor Cells in Developing Rat Cerebrum. *Int. J. Mol. Sci.* **2022**, *23*, 13199. https://doi.org/10.3390/ijms232113199.
99. Joo, J.-Y.; Schaukowitch, K.; Farbiak, L.; Kilaru, G.; Kim, T.-K. Stimulus-specific combinatorial functionality of neuronal c-fos enhancers. *Nat. Neurosci.* **2015**, *19*, 75–83. https://doi.org/10.1038/nn.4170.
100. Lu, C.; Jiang, Y.; Xu, W.; Bao, X. Sestrin2: Multifaceted functions, molecular basis, and its implications in liver diseases. *Cell Death Dis.* **2023**, *14*, 160. https://doi.org/10.1038/s41419-023-05669-4.
101. Baur, K.; Carrillo-García, C.; Şan, Ş.; von Hahn, M.; Strelau, J.; Hölzl-Wenig, G.; Mandl, C.; Ciccolini, F. Growth/differentiation factor 15 controls ependymal and stem cell number in the V-SVZ. *Stem Cell Rep.* **2024**, *19*, 351–365. https://doi.org/10.1016/j.stemcr.2024.01.008.
102. Li, L.; Mo, H.; Zhang, J.; Zhou, Y.; Peng, X.; Luo, X. The role of heat shock protein 90B1 in patients with polycystic ovary syndrome. *PLoS ONE* **2016**, *11*, e0152837. https://doi.org/10.1371/journal.pone.0152837.
103. Moon, B.-S.; Yoon, J.-Y.; Kim, M.-Y.; Lee, S.-H.; Choi, T.; Choi, K.-Y. Bone morphogenetic protein 4 stimulates neuronal differentiation of neuronal stem cells through the ERK pathway. *Exp. Mol. Med.* **2009**, *41*, 116–125. https://doi.org/10.3858/emm.2009.41.2.014.
104. Hwang, J.-Y.; Zukin, R.S. REST, a master transcriptional regulator in neurodegenerative disease. *Curr. Opin. Neurobiol.* **2018**, *48*, 193–200. https://doi.org/10.1016/j.conb.2017.12.008.
105. Peiris, M.; Hockley, J.R.; E Reed, D.; Smith, E.S.J.; Bulmer, D.C.; Blackshaw, L.A. Peripheral Kv7 channels regulate visceral sensory function in mouse and human colon. *Mol. Pain* **2017**, *13*, 1744806917709371. https://doi.org/10.1177/1744806917709371.
106. Zhong, L.; Fang, S.; Wang, A.-Q.; Zhang, Z.-H.; Wang, T.; Huang, W.; Zhou, H.-X.; Zhang, H.; Yin, Z.-S. Identification of the Fosl1/AMPK/autophagy axis involved in apoptotic and inflammatory effects following spinal cord injury. *Int. Immunopharmacol.* **2022**, *103*, 108492. https://doi.org/10.1016/j.intimp.2021.108492.
107. Singh, K.; Han, C.; Fleming, J.L.; Becker, A.P.; McElroy, J.; Cui, T.; Johnson, B.; Kumar, A.; Sebastian, E.; Showalter, C.A.; et al. TRIB1 confers therapeutic resistance in GBM cells by activating the ERK and Akt pathways. *Sci. Rep.* **2023**, *13*, 12424. https://doi.org/10.1038/s41598-023-32983-w.
108. Navarro, E.; Esteras, N. Multitarget Effects of Nrf2 Signalling in the Brain: Common and Specific Functions in Different Cell Types. *Antioxidants* **2024**, *13*, 1502. https://doi.org/10.3390/antiox13121502.

109. Kim, S.; Lee, W.; Jo, H.; Sonn, S.-K.; Jeong, S.-J.; Seo, S.; Suh, J.; Jin, J.; Kweon, H.Y.; Kim, T.K.; et al. The antioxidant enzyme Peroxiredoxin-1 controls stroke-associated microglia against acute ischemic stroke. *Redox Biol.* **2022**, *54*, 102347. https://doi.org/10.1016/j.redox.2022.102347.
110. Biermanns, M.; Gärtner, J. Genomic organization and characterization of human PEX2 encoding a 35-kDa peroxisomal membrane protein. *Biochem. Biophys. Res. Commun.* **2000**, *273*, 985–990. https://doi.org/10.1006/bbrc.2000.3039.
111. Winship, A.; Sorby, K.; Correia, J.; Rainczuk, A.; Yap, J.; Dimitriadis, E. Interleukin-11 up-regulates endoplasmic reticulum stress induced target, PDIA4 in human first trimester placenta and in vivo in mice. *Placenta* **2017**, *53*, 92–100. https://doi.org/10.1016/j.placenta.2017.04.007.
112. Petrova, K.; Oyadomari, S.; Hendershot, L.M.; Ron, D. Regulated association of misfolded endoplasmic reticulum lumenal proteins with P58/DNAJc3. *EMBO J.* **2008**, *27*, 2862–2872. https://doi.org/10.1038/emboj.2008.199.
113. Ferreira, L.P.d.S.; da Silva, R.A.; Borges, P.P.; Xavier, L.F.; Scharf, P.; Sandri, S.; Oliani, S.M.; Farsky, S.H.; Gil, C.D. Annexin A1 in neurological disorders: Neuroprotection and glial modulation. *Pharmacol. Ther.* **2025**, *267*, 108809. https://doi.org/10.1016/j.pharmthera.2025.108809.
114. Kollárovič, G.; E Topping, C.; Shaw, E.P.; Chambers, A.L. The human HELLS chromatin remodelling protein promotes end resection to facilitate homologous recombination and contributes to DSB repair within heterochromatin. *Nucleic Acids Res.* **2020**, *48*, 1872–1885. https://doi.org/10.1093/nar/gkz1146.
115. Jin, H.; Suh, D.-S.; Kim, T.-H.; Yeom, J.-H.; Lee, K.; Bae, J. IER3 is a crucial mediator of TAp73β-induced apoptosis in cervical cancer and confers etoposide sensitivity. *Sci. Rep.* **2015**, *5*, 8367. https://doi.org/10.1038/srep08367.
116. Raoul, C.; Estévez, A.G.; Nishimune, H.; Cleveland, D.W.; deLapeyrière, O.; Henderson, C.E.; Haase, G.; Pettmann, B. Motoneuron death triggered by a specific pathway downstream of fas: Potentiation by ALS-linked SOD1 mutations. *Neuron* **2002**, *35*, 1067–1083.
117. Chen, W.; Wang, H.; Tao, S.; Zheng, Y.; Wu, W.; Lian, F.; Jaramillo, M.; Fang, D.; Zhang, D.D. Tumor protein translationally controlled 1 is a p53 target gene that promotes cell survival. *Cell Cycle* **2013**, *12*, 2321–2328. https://doi.org/10.4161/cc.25404.
118. Lozinski, B.M.; Ta, K.; Dong, Y. Emerging role of galectin 3 in neuroinflammation and neurodegeneration. *Neural Regen. Res.* **2024**, *19*, 2004–2009. https://doi.org/10.4103/1673-5374.391181.
119. Hausott, B.; Klimaschewski, L. Sprouty2—A Novel Therapeutic Target in the Nervous System? *Mol. Neurobiol.* **2019**, *56*, 3897–3903. https://doi.org/10.1007/s12035-018-1338-8.
120. Cheng, Z.; Zou, X.; Jin, Y.; Gao, S.; Lv, J.; Li, B.; Cui, R. The role of KLF4 in Alzheimer's disease. *Front. Cell. Neurosci.* **2018**, *12*, 325. https://doi.org/10.3389/fncel.2018.00325.
121. Hao, Q.; Liu, Y.; Liu, Y.; Shi, L.; Chen, Y.; Yang, L.; Jiang, Z.; Liu, Y.; Wang, C.; Wang, S.; et al. Cysteine- and glycine-rich protein 1 predicts prognosis and therapy response in patients with acute myeloid leukemia. *Clin. Exp. Med.* **2024**, *24*, 57. https://doi.org/10.1007/s10238-023-01269-w.
122. Jiang, D.; de Vries, J.C.; Muschhammer, J.; Schatz, S.; Ye, H.; Hein, T.; Fidan, M.; Romanov, V.S.; Rinkevich, Y.; Scharffetter-Kochanek, K. Local and transient inhibition of p21 expression ameliorates age-related delayed wound healing. *Wound Repair Regen.* **2020**, *28*, 49–60. https://doi.org/10.1111/wrr.12763.
123. Gambarotta, G.; El Soury, M. Soluble neuregulin-1 (NRG1): A factor promoting peripheral nerve regeneration by affecting Schwann cell activity immediately after injury. *Neural Regen. Res.* **2019**, *14*, 1374–1375. https://doi.org/10.4103/1673-5374.253516.
124. Srivastava, S.; Ramdass, B.; Nagarajan, S.; Rehman, M.; Mukherjee, G.; Krishna, S. Notch1 regulates the functional contribution of RhoC to cervical carcinoma progression. *Br. J. Cancer* **2010**, *102*, 196–205. https://doi.org/10.1038/sj.bjc.6605451.
125. Yuan, J.; Yu, J. Human tissue factor pathway inhibitor-2 suppresses the wound-healing activities of human Tenon's capsule fibroblasts in vitro. *Mol. Vis.* **2009**, *15*, 2306–2312.
126. Farooq, M.; Khan, A.W.; Kim, M.S.; Choi, S. The role of fibroblast growth factor (FGF) signaling in tissue repair and regeneration. *Cells* **2021**, *10*, 3242. https://doi.org/10.3390/cells10113242.

127. Takouda, J.; Katada, S.; Imamura, T.; Sanosaka, T.; Nakashima, K. SoxE group transcription factor Sox8 promotes astrocytic differentiation of neural stem/precursor cells downstream of Nfia. *Pharmacol. Res. Perspect.* **2021**, *9*, e00749. https://doi.org/10.1002/prp2.749.
128. Kim, J.; Lo, L.; Dormand, E.; Anderson, D.J. SOX10 maintains multipotency and inhibits neuronal differentiation of neural crest stem cells. *Neuron* **2003**, *38*, 17–31. https://doi.org/10.1016/s0896-6273(03)00163-6.
129. Warner, L.E.; Mancias, P.; Butler, I.J.; McDonald, C.M.; Keppen, L.; Koob, K.G.; Lupski, J.R. Mutations in the early growth response 2 (EGR2) gene are associated with hereditary myelinopathies. *Nat. Genet.* **1998**, *18*, 382–384. https://doi.org/10.1038/ng0498-382.
130. Greene, L.A.; Lee, H.Y.; Angelastro, J.M. The transcription factor ATF5: Role in neurodevelopment and neural tumors. *J. Neurochem.* **2009**, *108*, 11–22. https://doi.org/10.1111/j.1471-4159.2008.05749.x.
131. Briscoe, J.; Sussel, L.; Serup, P.; Hartigan-O'Connor, D.; Jessell, T.M.; Rubenstein, J.L.R.; Ericson, J. Homeobox gene Nkx2.2 and specification of neuronal identity by graded Sonic hedgehog signalling. *Nature* **1999**, *398*, 622–627. https://doi.org/10.1038/19315.
132. Tu, M.; Zhu, P.; Hu, S.; Wang, W.; Su, Z.; Guan, J.; Sun, C.; Zheng, W. Notch1 signaling activation contributes to adult hippocampal neurogenesis following traumatic brain injury. *Med. Sci. Monit.* **2017**, *23*, 5480–5487. https://doi.org/10.12659/msm.907160.
133. Escobedo, N.; Contreras, O.; Muñoz, R.; Farías, M.; Carrasco, H.; Hill, C.; Tran, U.; Pryor, S.E.; Wessely, O.; Copp, A.J.; et al. Syndecan 4 interacts genetically with Vangl2 to regulate neural tube closure and planar cell polarity. *Development* **2013**, *140*, 3008–3017. https://doi.org/10.1242/dev.091173.
134. Wu, Y.; Peng, H.; Cui, M.; Whitney, N.P.; Huang, Y.; Zheng, J.C. CXCL12 increases human neural progenitor cell proliferation through Akt-1/FOXO3a signaling pathway. *J. Neurochem.* **2009**, *109*, 1157–1167. https://doi.org/10.1111/j.1471-4159.2009.06043.x.
135. Chi, S.; Cui, Y.; Wang, H.; Jiang, J.; Zhang, T.; Sun, S.; Zhou, Z.; Zhong, Y.; Xiao, B. Astrocytic Piezo1-mediated mechanotransduction determines adult neurogenesis and cognitive functions. *Neuron* **2022**, *110*, 2984–2999.e8. https://doi.org/10.1016/j.neuron.2022.07.010.
136. Tamura, R.E.; de Vasconcellos, J.F.; Sarkar, D.; Libermann, T.A.; Fisher, P.B.; Zerbini, L.F. GADD45 Proteins: Central Players in Tumorigenesis. *Curr. Mol. Med.* **2012**, *12*, 634–651.
137. Liu, C.; Chen, L.; Cong, Y.; Cheng, L.; Shuai, Y.; Lv, F.; Chen, K.; Song, Y.; Xing, Y. Protein phosphatase 1 regulatory subunit 15 A promotes translation initiation and induces G2M phase arrest during cuproptosis in cancers. *Cell Death Dis.* **2024**, *15*, 149. https://doi.org/10.1038/s41419-024-06489-w.
138. Grubbs, E.G.; Williams, M.D.; Scheet, P.; Vattathil, S.; Perrier, N.D.; Lee, J.E.; Gagel, R.F.; Hai, T.; Feng, L.; Cabanillas, M.E.; et al. Role of *CDKN2C* copy number in sporadic medullary thyroid carcinoma. *Thyroid*® **2016**, *26*, 1553–1562. https://doi.org/10.1089/thy.2016.0224.
139. Rogers, C.D.; Phillips, J.L.; Bronner, M.E. Elk3 is essential for the progression from progenitor to definitive neural crest cell. *Dev. Biol.* **2013**, *374*, 255–263. https://doi.org/10.1016/j.ydbio.2012.12.009.
140. Rudenok, M.M.; Shadrina, M.I.; Filatova, E.V.; Rybolovlev, I.N.; Nesterov, M.S.; Abaimov, D.A.; Ageldinov, R.A.; Kolacheva, A.A.; Ugrumov, M.V.; Slominsky, P.A.; et al. Expression Analysis of Genes Involved in Transport Processes in Mice with MPTP-Induced Model of Parkinson's Disease. *Life* **2022**, *12*, 751. https://doi.org/10.3390/life12050751.
141. Ould-Yahoui, A.; Tremblay, E.; Sbai, O.; Ferhat, L.; Bernard, A.; Charrat, E.; Gueye, Y.; Lim, N.H.; Brew, K.; Risso, J.-J.; et al. A new role for TIMP-1 in modulating neurite outgrowth and morphology of cortical neurons. *PLoS ONE* **2009**, *4*, e8289. https://doi.org/10.1371/journal.pone.0008289.
142. Huang, T.; Fu, G.; Gao, J.; Zhang, Y.; Cai, W.; Wu, S.; Jia, S.; Xia, S.; Bachmann, T.; Bekker, A.; et al. Fgr contributes to hemorrhage-induced thalamic pain by activating NF-κB/ERK1/2 pathways. *J. Clin. Investig.* **2020**, *5*, e139987. https://doi.org/10.1172/jci.insight.139987.
143. Islam, M.I.; Sultana, S.; Padmanabhan, N.; Rashid, M.-U.; Siddiqui, T.J.; Coombs, K.M.; Vitiello, P.F.; Karimi-Abdolrezaee, S.; Eftekharpour, E. Thioredoxin-1 protein interactions in neuronal survival and neurodegeneration. *Biochim. Et Biophys. Acta-Mol. Basis Dis.* 2025, 1871, 167548.
144. Mohammadzadeh, P.; Amberg, G.C. AXL/Gas6 signaling mechanisms in the hypothalamic-pituitary-gonadal axis. *Front. Endocrinol.* **2023**, *14*, 1212104. https://doi.org/10.3389/fendo.2023.1212104.

145. Zheng, S.-L.; Li, Z.-Y.; Song, J.; Liu, J.-M.; Miao, C.-Y. Metrnl: A secreted protein with new emerging functions. *Acta Pharmacol. Sin.* **2016**, *37*, 571–579. https://doi.org/10.1038/aps.2016.9.
146. Fischer-Huchzermeyer, S.; Dombrowski, A.; Hagel, C.; Mautner, V.F.; Schittenhelm, J.; Harder, A. The Cellular Retinoic Acid Binding Protein 2 Promotes Survival of Malignant Peripheral Nerve Sheath Tumor Cells. *Am. J. Pathol.* **2017**, *187*, 1623–1632. https://doi.org/10.1016/j.ajpath.2017.02.021.
147. Schaeffer, J.; Tannahill, D.; Cioni, J.-M.; Rowlands, D.; Keynes, R. Identification of the extracellular matrix protein Fibulin-2 as a regulator of spinal nerve organization. *Dev. Biol.* **2018**, *442*, 101–114. https://doi.org/10.1016/j.ydbio.2018.06.014.
148. Nonaka, M.; Fukuda, M. Galectin-1 for Neuroprotection? *Immunity* **2012**, *37*, 187–189. https://doi.org/10.1016/j.immuni.2012.08.006.
149. Chen, M.X.; Oh, Y.-S.; Kim, Y. S100A10 and its binding partners in depression and antidepressant actions. *Front. Mol. Neurosci.* **2022**, *15*, 953066. https://doi.org/10.3389/fnmol.2022.953066.
150. Iwanicka, J.; Balcerzyk-Matić, A.; Iwanicki, T.; Mizia-Stec, K.; Bańka, P.; Filipecki, A.; Gawron, K.; Jarosz, A.; Nowak, T.; Krauze, J.; et al. The Association of *ADAMTS7* Gene Polymorphisms with the Risk of Coronary Artery Disease Occurrence and Cardiovascular Survival in the Polish Population: A Case-Control and a Prospective Cohort Study. *Int. J. Mol. Sci.* **2024**, *25*, 2274. https://doi.org/10.3390/ijms25042274.
151. Pagnamenta, A.T.; Kaiyrzhanov, R.; Zou, Y.; Da'As, S.I.; Maroofian, R.; Donkervoort, S.; Dominik, N.; Lauffer, M.; Ferla, M.P.; Orioli, A.; et al. An ancestral 10-bp repeat expansion in *VWA1* causes recessive hereditary motor neuropathy. *Brain* **2021**, *144*, 584–600. https://doi.org/10.1093/brain/awaa420.
152. Konietzny, A.; Bär, J.; Mikhaylova, M. Dendritic actin cytoskeleton: Structure, functions, and regulations. *Front. Cell. Neurosci.* **2017**, *11*, 147. https://doi.org/10.3389/fncel.2017.00147.
153. Kim, N.C.; Andrews, P.C.; Asselbergs, F.W.; Frost, H.R.; Williams, S.M.; Harris, B.T.; Read, C.; Askland, K.D.; Moore, J.H. Gene ontology analysis of pairwise genetic associations in two genome-wide studies of sporadic ALS. *BioData Min.* **2012**, *5*, 9. https://doi.org/10.1186/1756-0381-5-9.
154. Notter, T.; Schalbetter, S.M.; Clifton, N.E.; Mattei, D.; Richetto, J.; Thomas, K.; Meyer, U.; Hall, J. Neuronal activity increases translocator protein (TSPO) levels. *Mol. Psychiatry* **2021**, *26*, 2025–2037. https://doi.org/10.1038/s41380-020-0745-1.
155. Silbereis, J.C.; Nobuta, H.; Tsai, H.-H.; Heine, V.M.; McKinsey, G.L.; Meijer, D.H.; Howard, M.A.; Petryniak, M.A.; Potter, G.B.; Alberta, J.A.; et al. Olig1 Function Is Required to Repress Dlx1/2 and Interneuron Production in Mammalian Brain. *Neuron* **2014**, *81*, 574–587. https://doi.org/10.1016/j.neuron.2013.11.024.
156. José, N.G.d.S.; Massa, F.; Halbgebauer, S.; Oeckl, P.; Steinacker, P.; Otto, M. Neuronal pentraxins as biomarkers of synaptic activity: From physiological functions to pathological changes in neurodegeneration. *J. Neural Transm.* **2022**, *129*, 207–230. https://doi.org/10.1007/s00702-021-02411-2.
157. Uzor, N.-E.; Scheihing, D.M.; Kim, G.S.; Moruno-Manchon, J.F.; Zhu, L.; Reynolds, C.R.; Stephenson, J.M.; Holmes, A.; McCullough, L.D.; Tsvetkov, A.S. Aging lowers PEX5 levels in cortical neurons in male and female mouse brains. *Mol. Cell. Neurosci.* **2020**, *107*, 103536. https://doi.org/10.1016/j.mcn.2020.103536.
158. Chapleau, A.; Boucher, R.-M.; Pastinen, T.; Thiffault, I.; Gould, P.V.; Bernard, G. Neuropathological characterization of the cavitating leukoencephalopathy caused by COA8 cytochrome c oxidase deficiency: A case report. *Front. Cell. Neurosci.* **2023**, *17*, 1216487. https://doi.org/10.3389/fncel.2023.1216487.
159. Mucha, M.; Skrzypiec, A.E.; Schiavon, E.; Attwood, B.K.; Kucerova, E.; Pawlak, R. Lipocalin-2 controls neuronal excitability and anxiety by regulating dendritic spine formation and maturation. *Proc. Natl. Acad. Sci. USA* **2011**, *108*, 18436–18441. https://doi.org/10.1073/pnas.1107936108.
160. Kolobynina, K.G.; Solovyova, V.V.; Levay, K.; Rizvanov, A.A.; Slepak, V.Z. Emerging roles of the single EF-hand Ca2+ sensor tescalcin in the regulation of gene expression, cell growth and differentiation. *J. Cell Sci.* **2016**, *129*, 3533–3540. https://doi.org/10.1242/jcs.191486.
161. Nixon, B.; Bromfield, E.G.; Dun, M.D.; Redgrove, K.; McLaughlin, E.; Aitken, R.J. The role of the molecular chaperone heat shock protein A2 (HSPA2) in regulating human sperm-egg recognition. *Asian J. Androl.* **2015**, *17*, 568–573,. https://doi.org/10.4103/1008-682x.151395.

162. Wang, J.; Owji, A.P.; Kittredge, A.; Clark, Z.; Zhang, Y.; Yang, T. GAD65 tunes the functions of Best1 as a GABA receptor and a neurotransmitter conducting channel. *Nat. Commun.* **2024**, *15*, 8051. https://doi.org/10.1038/s41467-024-52039-5.
163. Gotliv, I.L. FXYD5: Na+/K+-ATPase regulator in health and disease. *Front. Cell Dev. Biol.* **2016**, *4*, 26. https://doi.org/10.3389/fcell.2016.00026.
164. Shen, J.; Shi, D.; Suzuki, T.; Xia, Z.; Zhang, H.; Araki, K.; Wakana, S.; Takeda, N.; Yamamura, K.-I.; Jin, S.; et al. Severe ocular phenotypes in Rbp4-deficient mice in the C57BL/6 genetic background. *Mod. Pathol.* **2016**, *96*, 680–691. https://doi.org/10.1038/labinvest.2016.39.
165. Reichmann, F.; Holzer, P. Neuropeptide Y: A stressful review. *Neuropeptides* **2016**, *55*, 99–109. https://doi.org/10.1016/j.npep.2015.09.008.
166. Zhu, Z.; Bolt, E.; Newmaster, K.; Osei-Bonsu, W.; Cohen, S.; Cuddapah, V.A.; Gupta, S.; Paudel, S.; Samanta, D.; Dang, L.T.; et al. SCN1B Genetic Variants: A Review of the Spectrum of Clinical Phenotypes and a Report of Early Myoclonic Encephalopathy. *Children* **2022**, *9*, 1507. https://doi.org/10.3390/children9101507.
167. Pang, W.; Yi, X.; Li, L.; Liu, L.; Xiang, W.; Xiao, L. Untangle the Multi-Facet Functions of Auts2 as an Entry Point to Understand Neurodevelopmental Disorders. *Front. Psychiatry* **2021**, *12*, 580433. https://doi.org/10.3389/fpsyt.2021.580433.
168. Wagner, M.; Lévy, J.; Jung-Klawitter, S.; Bakhtiari, S.; Monteiro, F.; Maroofian, R.; Bierhals, T.; Hempel, M.; Elmaleh-Bergès, M.; Kitajima, J.P.; et al. Loss of TNR causes a nonprogressive neurodevelopmental disorder with spasticity and transient opisthotonus. *Anesthesia Analg.* **2020**, *22*, 1061–1068. https://doi.org/10.1038/s41436-020-0768-7.
169. Ageta-Ishihara, N.; Kinoshita, M. Developmental and postdevelopmental roles of septins in the brain. *Neurosci. Res.* **2021**, *170*, 6–12. https://doi.org/10.1016/j.neures.2020.08.006.
170. Cukier, H.N.; Duarte, C.L.; Laverde-Paz, M.J.; Simon, S.A.; Van Booven, D.J.; Miyares, A.T.; Whitehead, P.L.; Hamilton-Nelson, K.L.; Adams, L.D.; Carney, R.M.; et al. An Alzheimer's disease risk variant in TTC3 modifies the actin cytoskeleton organization and the PI3K-Akt signaling pathway in iPSC-derived forebrain neurons. *Neurobiol. Aging* **2023**, *131*, 182–195. https://doi.org/10.1016/j.neurobiolaging.2023.07.007.
171. Carvalho, S.D.-S.; Moreau, M.M.; Hien, Y.E.; Garcia, M.; Aubailly, N.; Henderson, D.J.; Studer, V.; Sans, N.; Thoumine, O.; Montcouquiol, M. Vangl2 acts at the interface between actin and N-cadherin to modulate mammalian neuronal outgrowth. *eLife* **2020**, *9*, e51822. https://doi.org/10.7554/elife.51822.
172. Rolando, C.; Erni, A.; Grison, A.; Beattie, R.; Engler, A.; Gokhale, P.J.; Milo, M.; Wegleiter, T.; Jessberger, S.; Taylor, V. Multipotency of Adult Hippocampal NSCs In Vivo Is Restricted by Drosha/NFIB. *Cell Stem Cell* **2016**, *19*, 653–662. https://doi.org/10.1016/j.stem.2016.07.003.
173. Ruiz-Gabarre, D.; Carnero-Espejo, A.; Ávila, J.; García-Escudero, V. What's in a Gene? The Outstanding Diversity of MAPT. *Cells* **2022**, *11*, 840. https://doi.org/10.3390/cells11050840.
174. Li, W.; Cheng, T.; Dong, X.; Chen, H.; Yang, L.; Qiu, Z.; Zhou, W. KIF5C deficiency causes abnormal cortical neuronal migration, dendritic branching, and spine morphology in mice. *Pediatr. Res.* **2022**, *92*, 995–1002. https://doi.org/10.1038/s41390-021-01922-8.
175. Sakabe, I.; Hu, R.; Jin, L.; Clarke, R.; Kasid, U.N. TMEM33: A new stress-inducible endoplasmic reticulum transmembrane protein and modulator of the unfolded protein response signaling. *Breast Cancer Res. Treat.* **2015**, *153*, 285–297. https://doi.org/10.1007/s10549-015-3536-7.
176. Martínez, J.C.; Randolph, L.K.; Iascone, D.M.; Pernice, H.F.; Polleux, F.; Hengst, U. Pum2 Shapes the Transcriptome in Developing Axons through Retention of Target mRNAs in the Cell Body. *Neuron* **2019**, *104*, 931–946.e5. https://doi.org/10.1016/j.neuron.2019.08.035.
177. Desole, C.; Gallo, S.; Vitacolonna, A.; Montarolo, F.; Bertolotto, A.; Vivien, D.; Comoglio, P.; Crepaldi, T. HGF and MET: From Brain Development to Neurological Disorders. *Front. Cell Dev. Biol.* **2021**, *9*, 683609. https://doi.org/10.3389/fcell.2021.683609.
178. Łuczyńska, K.; Zhang, Z.; Pietras, T.; Zhang, Y.; Taniguchi, H. NFE2L1/Nrf1 serves as a potential therapeutical target for neurodegenerative diseases. *Redox Biol.* **2024**, *69*, 103003. https://doi.org/10.1016/j.redox.2023.103003.

179. Higelin, J.; Catanese, A.; Semelink-Sedlacek, L.L.; Oeztuerk, S.; Lutz, A.-K.; Bausinger, J.; Barbi, G.; Speit, G.; Andersen, P.M.; Ludolph, A.C.; et al. NEK1 loss-of-function mutation induces DNA damage accumulation in ALS patient-derived motoneurons. *Stem Cell Res.* **2018**, *30*, 150–162. https://doi.org/10.1016/j.scr.2018.06.005.
180. Santo, E.E.; Paik, J. FOXO in Neural Cells and Diseases of the Nervous System. In *Current Topics in Developmental Biology*; Elsevier BV: Amsterdam, The Netherlands, 2018; Volume 127, pp. 105–118.
181. Klopf, E.; Schmidt, H.A.; Clauder-Münster, S.; Steinmetz, L.M.; Schüller, C. INO80 represses osmostress induced gene expression by resetting promoter proximal nucleosomes. *Nucleic Acids Res.* **2017**, *45*, 3752–3766. https://doi.org/10.1093/nar/gkw1292.
182. Yue, W.; Zhang, K.; Jiang, M.; Long, W.; Cui, J.; Li, Y.; Zhang, Y.; Li, A.; Fang, Y. Deubiquitination of SARM1 by USP13 regulates SARM1 activation and axon degeneration. *Life Med.* **2023**, *2*, lnad040. https://doi.org/10.1093/lifemedi/lnad040.
183. Zhang, Z.; Zhao, Y. Progress on the roles of MEF2C in neuropsychiatric diseases. *Mol. Brain* **2022**, *15*, 8. https://doi.org/10.1186/s13041-021-00892-6.
184. Renkilaraj, M.R.L.M.; Baudouin, L.; Wells, C.M.; Doulazmi, M.; Wehrlé, R.; Cannaya, V.; Bachelin, C.; Barnier, J.-V.; Jia, Z.; Oumesmar, B.N.; et al. The intellectual disability protein PAK3 regulates oligodendrocyte precursor cell differentiation. *Neurobiol. Dis.* **2017**, *98*, 137–148. https://doi.org/10.1016/j.nbd.2016.12.004.
185. Flores, C.E.; Méndez, P. Shaping inhibition: Activity dependent structural plasticity of GABAergic synapses. *Front. Cell. Neurosci.* **2014**, *8*, 327. https://doi.org/10.3389/fncel.2014.00327.
186. Hussain, N.K.; Hsin, H.; Huganir, R.L.; Sheng, M. MINK and TNIK differentially act on Rap2-mediated signal transduction to regulate neuronal structure and AMPA receptor function. *J. Neurosci.* **2010**, *30*, 14786–14794. https://doi.org/10.1523/jneurosci.4124-10.2010.
187. Aabdien, A.; Sichlinger, L.; Borgel, Z.; Jones, M.R.; Waston, I.A.; Gatford, N.J.F.; Raval, P.; Tanangonan, L.; Powell, T.R.; Duarte, R.R.R.; et al. Schizophrenia risk proteins ZNF804A and NT5C2 interact in cortical neurons. *Eur. J. Neurosci.* **2024**, *59*, 2102–2117. https://doi.org/10.1111/ejn.16254.
188. Guo, H.; Bettella, E.; Marcogliese, P.C.; Zhao, R.; Andrews, J.C.; Nowakowski, T.J.; Gillentine, M.A.; Hoekzema, K.; Wang, T.; Wu, H.; et al. Disruptive mutations in TANC2 define a neurodevelopmental syndrome associated with psychiatric disorders. *Nat. Commun.* **2019**, *10*, 4679. https://doi.org/10.1038/s41467-019-12435-8.
189. Assali, A.; Chenaux, G.; Cho, J.Y.; Berto, S.; Ehrlich, N.A.; Cowan, C.W. EphB1 controls long-range cortical axon guidance through a cell non-autonomous role in GABAergic cells. *Development* **2024**, *151*, dev201439. https://doi.org/10.1242/dev.201439.
190. Nagy, G.N.; Zhao, X.-F.; Karlsson, R.; Wang, K.; Duman, R.; Harlos, K.; El Omari, K.; Wagner, A.; Clausen, H.; Miller, R.L.; et al. Structure and function of Semaphorin-5A glycosaminoglycan interactions. *Nat. Commun.* **2024**, *15*, 2723. https://doi.org/10.1038/s41467-024-46725-7.
191. Le, V.-H.; Orniacki, C.; Murcia-Belmonte, V.; Denti, L.; Schütz, D.; Stumm, R.; Ruhrberg, C.; Erskine, L. CXCL12 promotes the crossing of retinal ganglion cell axons at the optic chiasm. *Development* **2024**, *151*, dev202446. https://doi.org/10.1242/dev.202446.
192. Chang, C.; Banerjee, S.L.; Park, S.S.; Zhang, X.L.; Cotnoir-White, D.; Opperman, K.J.; Desbois, M.; Grill, B.; Kania, A. Ubiquitin ligase and signalling hub MYCBP2 is required for efficient EPHB2 tyrosine kinase receptor function. *eLife* **2024**, *12*, RP89176.
193. Koga, M.; Ishiguro, H.; Yazaki, S.; Horiuchi, Y.; Arai, M.; Niizato, K.; Iritani, S.; Itokawa, M.; Inada, T.; Iwata, N.; et al. Involvement of SMARCA2/BRM in the SWI/SNF chromatin-remodeling complex in schizophrenia. *Hum. Mol. Genet.* **2009**, *18*, 2483–2494. https://doi.org/10.1093/hmg/ddp166.
194. El Hayek, L.; Tuncay, I.O.; Nijem, N.; Russell, J.; Ludwig, S.; Kaur, K.; Li, X.; Anderton, P.; Tang, M.; Gerard, A.; et al. Kdm5a mutations identified in autism spectrum disorder using forward genetics. *eLife* **2020**, *9*, e56883. https://doi.org/10.7554/elife.56883.
195. Condylis, C.; Ghanbari, A.; Manjrekar, N.; Bistrong, K.; Yao, S.; Yao, Z.; Nguyen, T.N.; Zeng, H.; Tasic, B.; Chen, J.L. Dense functional and molecular readout of a circuit hub in sensory cortex. *Science* **2022**, *375*, eabl5981. https://doi.org/10.1126/science.abl5981.

196. Sollazzo, R.; Puma, D.D.L.; Aceto, G.; Paciello, F.; Colussi, C.; Vita, M.G.; Giuffrè, G.M.; Pastore, F.; Casamassa, A.; Rosati, J.; et al. Structural and functional alterations of neurons derived from sporadic Alzheimer's disease hiPSCs are associated with downregulation of the LIMK1-cofilin axis. *Alzheimer's Res. Ther.* **2024**, *16*, 267. https://doi.org/10.1186/s13195-024-01632-3.
197. Baumgärtel, K.; Green, A.; Hornberger, D.; Lapira, J.; Rex, C.; Wheeler, D.G.; Peters, M. PDE4D regulates Spine Plasticity and Memory in the Retrosplenial Cortex. *Sci. Rep.* **2018**, *8*, 3895. https://doi.org/10.1038/s41598-018-22193-0.
198. Nishioka, M.; Shimada, T.; Bundo, M.; Ukai, W.; Hashimoto, E.; Saito, T.; Kano, Y.; Sasaki, T.; Kasai, K.; Kato, T.; et al. Neuronal cell-type specific DNA methylation patterns of the *Cacna1c* gene. *Int. J. Dev. Neurosci.* **2013**, *31*, 89–95. https://doi.org/10.1016/j.ijdevneu.2012.11.007.
199. Muresan, V.; Abramson, T.; Lyass, A.; Winter, D.; Porro, E.; Hong, F.; Chamberlin, N.L.; Schnapp, B.J. KIF3C and KIF3A form a novel neuronal heteromeric kinesin that associates with membrane vesicles. *Mol. Biol. Cell* **1998**, *9*, 637–652. https://doi.org/10.1091/mbc.9.3.637.
200. Kanai, Y.; Okada, Y.; Tanaka, Y.; Harada, A.; Terada, S.; Hirokawa, N. KIF5C, a novel neuronal kinesin enriched in motor neurons. *J. Neurosci.* **2000**, *20*, 6374–6384. https://doi.org/10.1523/jneurosci.20-17-06374.2000.
