# Supplementary figures and images for: Extracellular Vesicles from iPSC-Derived Glial Progenitor Cells Prevent Glutamate-Induced Excitotoxicity by Stabilising Calcium Oscillations and Mitochondrial Depolarisation
Source: Cells. 2025 Dec 2;14(23):1915. doi: 10.3390/cells14231915 (PMC12691032; doi:10.3390/cells14231915)

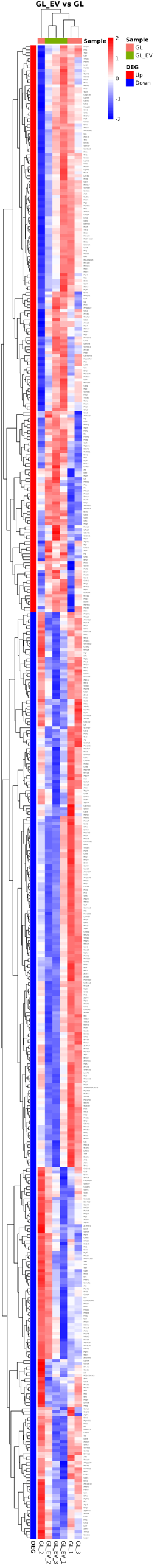

Supplement: Supplementary file 1 [file cells-14-01915-s001.zip › heatmap_big_GL_EV_vs_GL.tif]

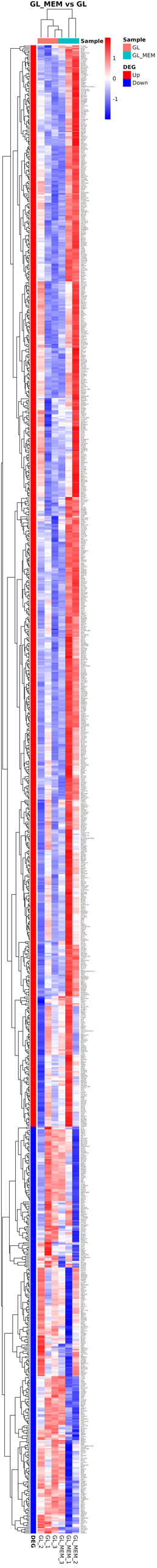

Supplement: Supplementary file 1 [file cells-14-01915-s001.zip › heatmap_big_GL_MEM_vs_GL.tif]

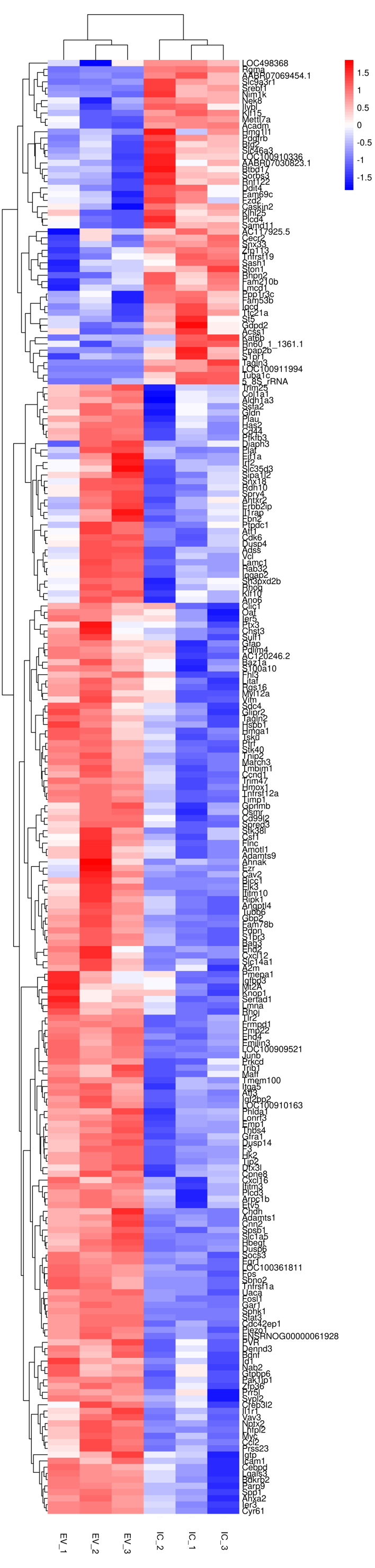

Supplement: Supplementary file 1 [file cells-14-01915-s001.zip › heatmap_big_IC_vs_EV.tif]

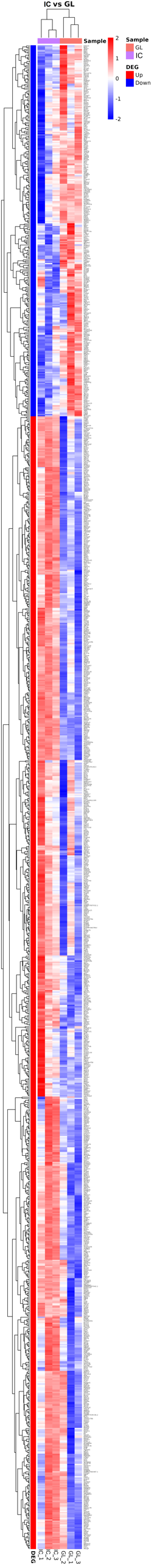

Supplement: Supplementary file 1 [file cells-14-01915-s001.zip › heatmap_big_IC_vs_GL.tif]

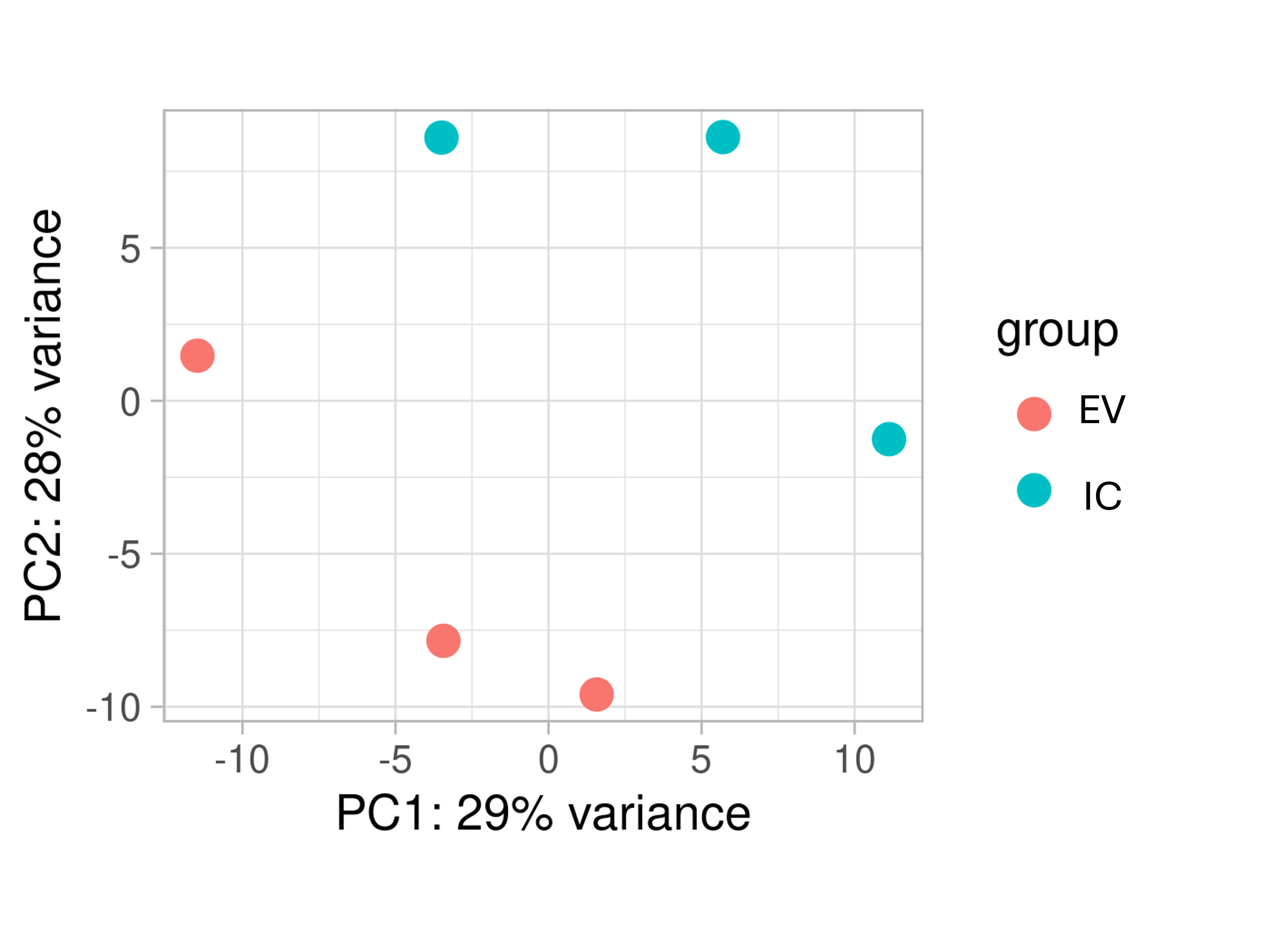

Supplement: Supplementary file 1 [file cells-14-01915-s001.zip › pca_plot_IC_vs_EV.tif]

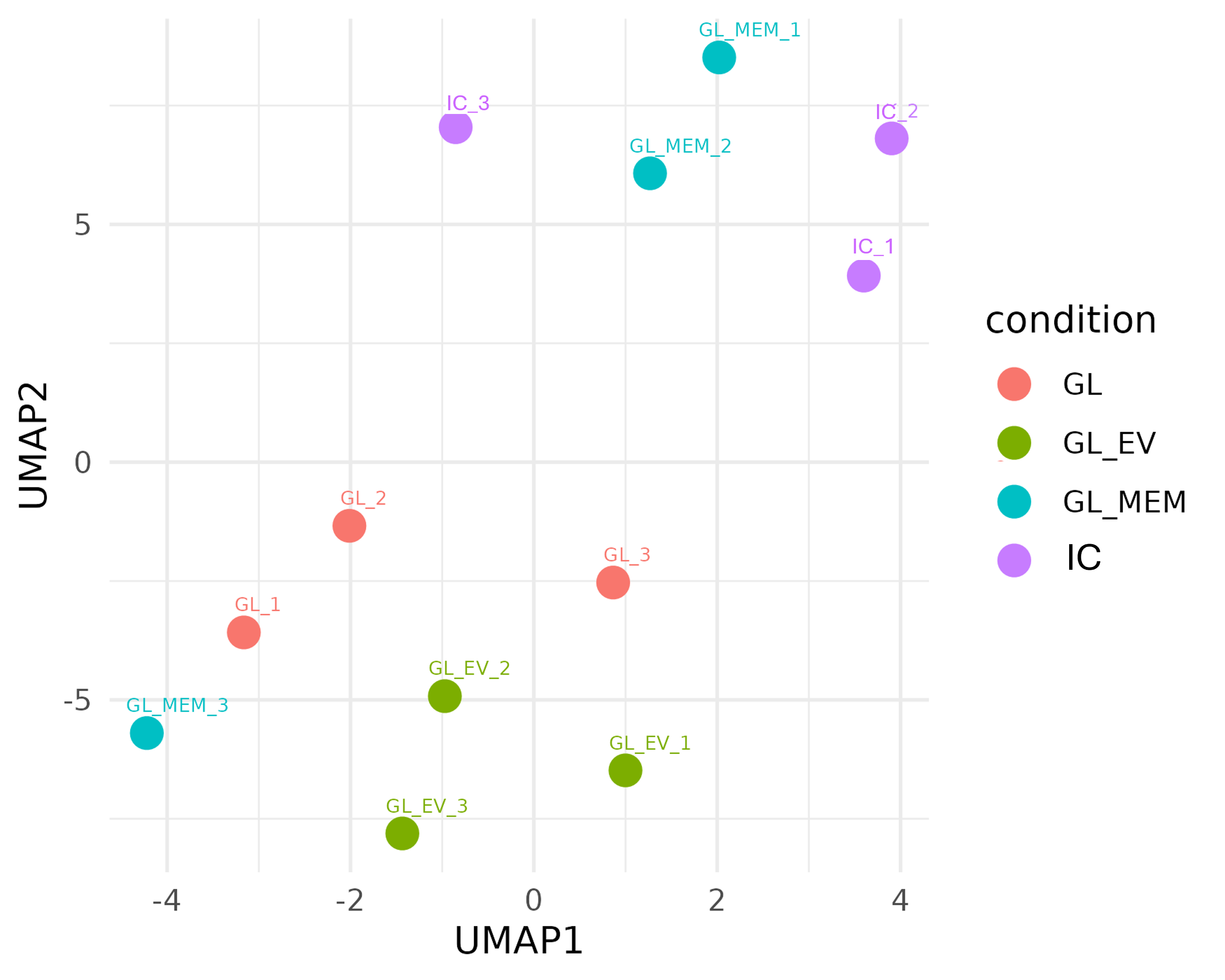

Supplement: Supplementary file 1 [file cells-14-01915-s001.zip › umap_plot_IC_GL_GL_EV_GL_Mem.tif]
